# Betulin Alleviates the Inflammatory Response in Mouse Chondrocytes and Ameliorates Osteoarthritis via AKT/Nrf2/HO-1/NF-κB Axis

**DOI:** 10.3389/fphar.2021.754038

**Published:** 2021-10-13

**Authors:** Chenghao Ren, Jie Jin, Wei Hu, Qi Chen, Jian Yang, Yaosen Wu, Yifei Zhou, Liaojun Sun, Weiyang Gao, Xiaolei Zhang, Naifeng Tian

**Affiliations:** ^1^ Department of Orthopaedics, the Second Affiliated Hospital and Yuying Children’s Hospital of Wenzhou Medical University, Wenzhou, China; ^2^ The Second School of Medicine, Wenzhou Medical University, Wenzhou, China; ^3^ Zhejiang Provincial Key Laboratory of Orthopaedics, Wenzhou, China; ^4^ Chinese Orthopaedic Regenerative Medicine Society, Hangzhou, China

**Keywords:** betulin, osteoarthritis (OA), AKT, NRF, NF-κB, inflammation

## Abstract

Osteoarthritis (OA) is a common degenerative joint disease featuring the degeneration, destruction, and ossification of cartilage. Inflammation which may facilitate OA occurrence and development is considered as the main pathological factor. Betulin, a natural product extracted from birch bark, has been commonly used for inflammation treatment; however, its role in OA remains unclear. This study is aimed to explore whether betulin can suppress IL-1β–induced inflammation in chondrocytes and alleviate OA *in vitro* and *in vivo*. In *in vitro* studies, the generation of pro-inflammatory factors, such as interleukin-6 (IL-6), tumor necrosis factor alpha (TNF-α), prostaglandin E2 (PGE2), and nitric oxide (NO), was assessed using the enzyme-linked immunosorbent assay (ELISA) and Griess reaction. As revealed by results, betulin inhibited the expression of pro-inflammatory mediators. In addition, the protein expressions of inducible nitric oxide synthase (iNOS), cyclooxygenase-2 (COX-2), matrix metalloproteinase (MMP-13), thrombospondin motifs 5 (ADAMTS5), Collagen II, and Aggrecan were quantified using Western blot analysis. We found that betulin could inhibit the generation of COX-2 and iNOS induced by IL-1β, indicating that betulin has anti-inflammatory effects in chondrocytes. Furthermore, betulin downregulates the expression of MMP-13 and ADAMTS-5 and upregulates the expression of Collagen II and Aggrecan, indicating that it can inhibit the degradation of the extracellular matrix. In mechanism, betulin activated the AKT/Nrf2 pathway and inhibited the phosphorylation of p65. In *in vivo* studies, administration of betulin *in vivo* could inhibit cartilage destruction and inflammatory progression. Therefore, these findings suggest that betulin may alleviate IL-1β–induced OA via the AKT/Nrf2/HO-1/NF-κB signal axis, and betulin may be a potential drug for the treatment of OA.

## Introduction

Osteoarthritis (OA) is a long-lasting, chronic, and progressive joint disease characterized by the loss or destruction of joint function and morphological integrity. It is mainly manifested as joint pain with serious impact on daily life (Kean et al., 2004). However, the specific pathogenesis of OA remains unclear (Sun et al., 2017). In addition, due to the insufficient efficacy of current drug therapy, clinical symptoms can be temporarily relieved, urgently requiring drugs with good efficacy and less side effects to reverse the progress of OA (Krustev et al., 2015). According to the increasing evidence, inflammatory mechanisms play a key role in the progression of OA (Bonnet and Walsh, 2005; Scanzello, 2017). A large number of inflammatory cytokines, such as interleukin-6 (IL-6), interleukin-1β (IL-1β), and tumor necrosis factor-α (TNF-α) are involved in OA occurrence and development (Fernandes et al., 2002), among which IL-1β is the main pro-inflammatory cytokine. Previous studies have revealed that IL-1β can induce the activation of nuclear transcription factor B (NF-κB) and promote the secretion of pro-inflammatory mediators (Abramson et al., 2001; Vincenti and Brinckerhoff, 2002; Chabane et al., 2008; Zhang et al., 2018), resulting in the decomposition of the intra-articular matrix above the synthetic level (Dodge and Poole, 1989; Nummenmaa et al., 2015), and finally accelerating the deterioration of OA. Therefore, inhibiting the IL-1β–mediated inflammatory response is an effective therapeutic strategy to suppress the progression of OA.

There is evidence that the NF-κB signal pathway can regulate the expression of inflammatory mediators and participate in the inflammatory process, such as IL-1β–induced inflammation and apoptosis (Wang et al., 2016). The stimulation of IL-1β will lead to the degradation of IκBα, release of the bound NF-κB dimer, and activation of the NF-κB signal pathway, thereby causing inflammation (Kumar et al., 2004; Hayden and Ghosh, 2011). Studies have shown that the nuclear factor–erythroid 2–related factor (Nrf2) and heme oxygenase-1 (HO-1) are important signal mediators in anti-inflammatory defense mechanisms, and the activation of the Nrf2/HO-1 signal can inhibit inflammatory effects (Jiang et al., 2014). In addition, we also found that by activating AKT, the expression of HO-1 can be regulated via the Nrf2 pathway (Lu et al., 2014; Huang et al., 2015; Yeh et al., 2015). According to reports, many natural products with anti-inflammatory activity exert anti-inflammatory protection by inducing HO-1 (Parada et al., 2015; Chan et al., 2017). Therefore, the NF-κB or HO-1 pathway may be a potential therapeutic target.

Betulin is a triterpene natural product extracted from birch bark, with anti-inflammatory activity that can be used to control various inflammatory diseases in folk medicine (Bai et al., 2016). For example, betulin attenuates renal injury in septicemic rats by inhibiting TLR4/NF-κB signal pathway (Zhao et al., 2016). In addition, it has been found that betulin inhibits IL-1β–induced matrix metalloproteinase gene expression and production (Ra et al., 2017), suggesting that betulin may delay the progression of OA, but the mechanism on how exactly it slows down the development of inflammation is unclear; this study specifically elaborated the specific mechanism of betulin in the progress of OA. It is worth noting that the role of the AKT/Nrf2/HO-1/NF-κB signal axis in the progression of OA has not been clarified yet. This study aims to explore whether betulin can reduce the inflammation level of OA by regulating the signal axis of AKT/Nrf2/HO-1/NF-κB, thus alleviating the progression of OA.

## Materials and Methods

### Reagents

Betulin (purity ≥98%) was dissolved in dimethylsulfoxide (DMSO), diluted in PBS, and administered in a culture medium (final concentrations of DMSO were 0.5%), as 0.5% DMSO alone did not affect the proliferation of chondrocytes. Type II collagenase was purchased from Solarbio (Beijing, China). Recombinant IL-1β was obtained from Novoprotein (China). The primary antibodies against Aggrecan (ab3778), Collagen II (ab34712), MMP-13 (ab1010), ADAMTS5 (ab41037), HO-1 (ab13248), Nrf2 (ab92946), AKT (ab8805), Lamin B (ab16048), GAPDH (ab8245), P65 (ab16502), IκBα (ab32518), P-IκBα (ab133462), and P-P65 (ab76302) were purchased from Cambridge ABCAM Company in England. Cell count kit-8 (CCK-8) was purchased from Dojindo (Kumamoto, Japan). 6-diamino-2-phenylindole (DAPI) nuclear staining was obtained from Beyotime, Shanghai, China. Griess reagent is provided by Biomass Life Sciences Co., Ltd. (Shanghai, China). ELISA kits for tumor necrosis factor-α (TNF-α), interleukin-6 (IL-6), and prostaglandin E2 (PGE-2) were provided by the Minneapolis R & D system.

### Isolation and Culture of Primary Chondrocytes

10-week-old C57BL/6 male wildtype (WT) mice were purchased from the Animal Center of the Chinese Academy of Sciences (Shanghai). After euthanasia with 10% chloral hydrate, the knee joint and hip cartilage of the mice were washed in phosphate-buffered saline (PBS) and minced into pieces measuring approximately 2 mm. The cartilage tissue was digested for 4 h with 0.2% Type II collagenase at 37°C. The digested cartilage samples were centrifuged at 37°C for 3 min. The supernatant was inoculated in a culture dish (seeding density: 10^5^ cells/cm^2^). 10% fetal bovine serum (FBS) and 1% antibiotic (penicillin**/**streptomycin) DMEM/F-12 were incubated at 37 °C, 5% CO_2_, and the culture medium was replaced every day. 80 and 90% were mixed and separated with 0.25% trypsin-EDTA solution and then cultured. In this study, only 0–2 generation cells were used to avoid phenotypic loss (Wu et al., 2018) (Zheng et al., 2018).

### CCK-8 Assay

To evaluate the potential toxicity of betulin to mouse chondrocytes, the CCK-8 (Dojindo Co, Kumamoto, Japan) test was performed in accordance with the manufacturer’s instructions. In short, cartilage cells were seeded at a density of 2 × 10^5^/mL (0.1 mL/well) in a 96-well plate and allowed to attach for 24 h to keep the log phase growth at the time of drug treatment, then treated with betulin under a concentration gradient of (0, 25, 50, 100, and 200 μM) for 24 h or 48 h, 10 μL CCK-8 solution was added to each well and incubated at 37°C for 4 h. Then the optical density was measured by spectrophotometer (Thermo Fisher) at 450 nm. All the experiments were carried out for three times.

### Determination of NO, PGE2, TNF-α, and IL-6

Chondrocytes cultured in culture dishes were exposed to betulin (0, 25, 50, and 100 μM) of different concentrations, incubated at 37°C for 2°h, then incubated with IL-1β (10 ng/ml) for 24 h. The concentration of NO was determined by Griess reaction. The concentrations of PGE2, TNF-α, and IL-6 in each culture were determined by the ELISA method (Au et al., 2007). All the tests were carried out for three times.

### Western Blotting

Chondrocytes were incubated for 2 h in growth medium with 25, 50, and 100 μM of betulin followed by incubation in the presence or absence of IL-1β (10 ng/mL) for 24 h. Cells were collected and lysed with Radioimmunoprecipitation Assay (RIPA) lysis buffer [with 1% phenylmethanesulfonyl fluoride (PMSF)], the nucleoproteins of cells were obtained using a nuclear extraction kit. The protein concentration was quantified using a bicinchoninic acid (BCA) protein assay kit. Sodium dodecyl sulfate–polyacrylamide gel electrophoresis (SDS-PAGE) and polyvinylidene fluoride (PVDF) membrane were used to separate and transfer 40 ng proteins, and then sealed with 5% skim milk for 2 h. Then, the primary antibody was used overnight at 4°C. After the primary antibody was incubated, the membrane was washed with Tris-buffered Saline with 0.1% Tween-20 (TBST) for three times for 5 min, and then incubated at room temperature in 5% TBST for 2 h with the corresponding secondary antibody. After being rinsed by TBST, the film was developed with the enhanced chemiluminescence kit, and the gray value of the film was quantitatively determined by Image Lab 3.0 software (Bio-Rad).

### Real-Time PCR

Chondrocytes were inoculated into 6-well plates. After 24 h of culture, the cultured chondrocytes were stimulated with different concentrations (25, 50, and 100 μM) of betulin followed by incubation for 24 h with or without IL-1β (10 ng/mL). The total RNA of monolayer chondrocytes was extracted with TRIzol reagent. The concentration of RNA was determined by spectrophotometry at 260 nm, and the quality and purity of RNA were determined by the A260/A280 ratio. The first-strand cDNA was synthesized with 10 ng total RNA and the QuantiTect reverse transcription kit. The CFX96 real-time PCR (RT-PCR) system was used for 10-min reaction at 95°C, 15-min reaction at 95 °C, and 1-min reaction at 60 °C, with a circulation of 40 times, and the total reaction volume was 10 μL (4.5 μL diluted cDNA, 0.25 μL forward primer, 0.25 μL reverse primer, and 5 μL SYBR Green Master Mix). The target mRNA level was normalized to the GAPDH level and compared with the control group. The primers for iNOS, IL-6, COX-2, and TNF-α were designed as follows (Pfaffl, 2001):

iNOS (F)5‘GACGAGACGGATAGGCAGAG3’ (R)5‘CATGCAAGGAAGGAACT3’

IL-6 (F)5‘CCGGAGAGGAGACTTCAG3’ (R)5‘TCCACGATTTCCCAGAGAAC3′

COX-2 (F)5‘TCCTCACATCCCTGAGAACC3’ (R)5‘GTCGCACACTCTGTTGTGCT3′

TNF-α (F)5‘ACGGCATGGATCTCAAAGAC3’ (R)5‘GTGGGTGAGGAGCACGTAGT3′

### Immunofluorescence Analysis

Chondrocytes incubated with or without betulin (50 μM) for 2 h, then co-incubated with IL-1β (10 ng/ml) for 24 h, fixed with 4% paraformaldehyde solution at room temperature for 15 min, rinsed using PBS for three times, and then treated with 0.1%TritonX-100 at room temperature for 5 min. The cover slides were placed in a wet box, sealed at 37°C with 5% bovine serum albumin for 1 h, then diluted with primary antibodies at 4°C for 12°h, rinsed by phosphate buffer, incubated with goat anti-rabbit IgG antibody coupled with fluorescein at room temperature for 1 h, and then stained with DAPI. The fluorescence microscope was used for imaging.

### Animal Model

10-weeks-old C57BL/6 male wild-type (WT) mice (n = 18) were selected from the Shanghai Animal Center of Chinese Academy of Sciences. All the experiments were performed in accordance with the regulations of the Animal Protection and Utilization Committee of Wenzhou Medical University (wyde2021-0297). The mouse OA model was established by surgical resection of medial meniscus (DMM). Mice were anesthetized with 1% pentobarbital sodium, then the right knee joint was cut open with microsurgical scissors and the medial meniscus tibial ligament of the right knee joint was transected to protect the lateral meniscus ligament during the operation. As a control, the medial meniscus tibial ligament was not transected, while the left knee arthrotomy was performed as the sham operation group. The mice were randomly divided into three groups: the first group was the Sham group, the second group was the DMM group, and the third group was the DMM+Betulin group (n = 6 mice in each group). After operation, the mice in the third group were injected intraperitoneally with betulin (20 mg/kg/d) (Ma and Long, 2016), and the mice in the other groups were injected with the same amount of inert carrier (DMSO) for 8 weeks under the temperature of 20 ± 2°C, with a relative humidity of 50 ± 10%, and the light/dark cycle of 12 h. All animals were killed 8 weeks after the operation, and the cartilage samples were taken for immunological and histological analyses.

### Histopathological Analysis

Cartilage destruction was evaluated using SO and HE staining, and the slides of each joint were stained. Then, the morphological changes of the cartilage and its surrounding tissue were observed using a microscope, and articular cartilage destruction was evaluated using the Osteoarthritis Research Society International (OARSI) medial femoral condyle and medial tibial plateau scoring systems.

### Immunohistochemical Analysis

Knee joint tissue was fixed with 4% paraformaldehyde, decalcified, and embedded in paraffin, then it was sliced, dewaxed, and rehydrated, treated with 3% (v/w) hydrogen peroxide and 0.25% trypsin-EDTA solution at 37°C for 30 min. Then, the slices were incubated in 10% bovine serum albumin for 60 min (37°C). The first antibody was treated at 4°C for 24 h. On the second day, the secondary antibody was incubated at 4°C for 1 h. The images were analyzed using Image-ProPlus6.0 version software. Five slices were taken from each group for quantitative analyses.

### X-Ray Imaging

X-ray examination was performed on the mice in the three groups at the eighth week after operation. X-ray imaging was performed on all animals using digital X-ray system. The degree of articular cartilage degeneration was evaluated by joint space, cartilage surface calcification, and osteophyte formation.

### Molecular Docking Model

The molecular structure of betulin was derived from PubChem database and imported into Chem3D for molecular energy minimization and geometric optimization. The protein structure of AKT (PDBID:4GV1) was from the Protein Data Bank database (http://www.rcsb.org/). The structure of the protein was treated on the Maestro11.9 platform. The Protein Preparation Wizard of Schrodinger was used to treat the protein, remove the crystal water, add the missing hydrogen atom, repair the missing bond information, repair the missing peptide, and finally minimize the energy and optimize the geometric structure of the protein (Rajeswari et al., 2014; Fazi et al., 2015). All molecules were prepared according to the default settings of the Lig Prep module. When screening in the Glide module, the prepared receptor was introduced to specify the appropriate location in the receptor grid generation, the predicted active site of the protein was selected as the centroid of the 12 Å box. Finally, molecular docking and screening were carried out by the standard method.

### Statistical Analysis

All experiments were performed at least three times. All data were presented as the mean ± SD and carried out by SPSS 20.0 software. The differences between groups were analyzed by one-way ANOVA with Tukey's multiple comparison test. *p* < 0.05 and *p* < 0.01 were considered to be significant.

## Results

### The Effect of Betulin on the Viability of Chondrocytes

**FIGURE 1:**
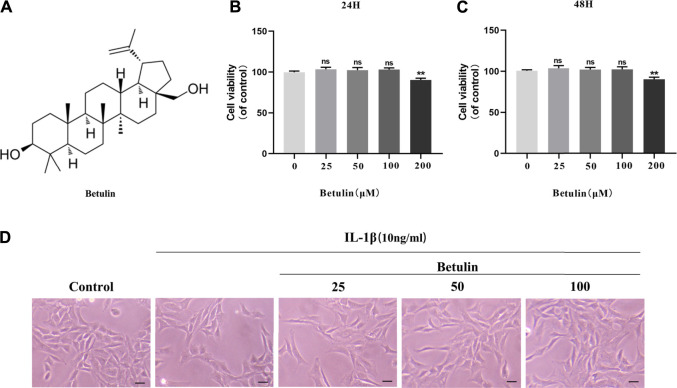
Effect of betulin on the viability of OA chondrocytes. **(A)** The chemical structure of betulin. **(B**–**C)** The cell was cultured with betulin (0, 25, 50, 100, and 200 μM) different concentrations for 24 and 48 h, and the cell viability was measured using the CCK-8 kit. **(D)** After the chondrocytes were pretreated with different concentrations of betulin, the chondrocytes were imaged with a phase contrast microscope (scale bar: 50 μm). The data in the figure represent the average ± SD, **p < 0.01 compared to the control group, n = 3.

The chemical structure of betulin is shown in Figure 1A. The CCK-8 experiment was performed to determine the cytotoxic effect of betulin on chondrocytes. Chondrocytes were incubated with betulin of different concentrations (0, 25, 50, 100, and 200 μM) for 24 and 48 h. As shown by Figures 1B–C, the cell viability of chondrocytes were not significantly affected at the concentration of 0–100 μM but decreased slightly at the concentration of 200 μM (*p* < 0.01). The results of treatment for 48 h were similar (*p* < 0.01). Besides, under IL-1β treatment, the number of chondrocytes decreased and the cells shrank. After betulin treatment, the survival of the chondrocytes increased in a dose-dependent manner. Therefore, betulin with the concentrations of (0, 25, 50, and 100 μM) was used in all subsequent experiments.

### Betulin Inhibits the Generation of Pro-inflammatory Mediators

**FIGURE 2:**
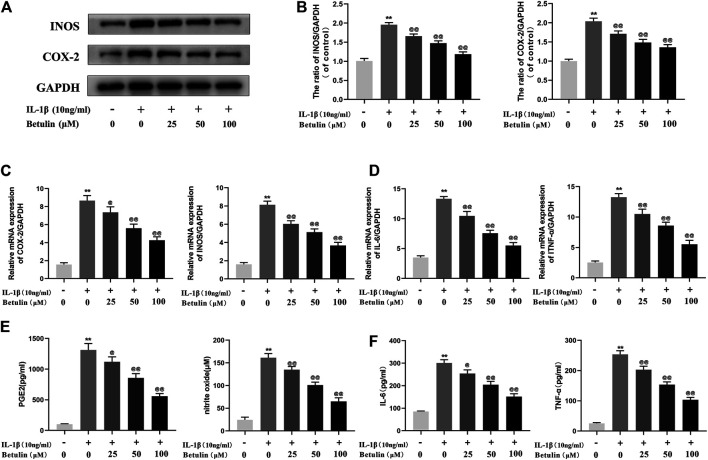
Betulin treatment can inhibit the generation of pro-inflammatory mediators. OA chondrocytes were pretreated with betulin (0, 25, 50, and 100 μM) of different concentrations for 2 h, then stimulated by IL-1β (10 ng/ml) for 24 h. **(A**–**B)** The protein expression of iNOS and COX-2 was detected by Western blot and quantitatively analyzed. **(C**–**D)** The mRNA expression levels of iNOS, COX-2, IL-6, and TNF-α were detected by RT-PCR. **(E**–**F)** The generation of NO, PGE2, TNF-α, and IL-6 was detected by Griess reaction and the ELISA method. The data in the figure are represented as the average ±SD, *p < 0.05, **p < 0.01 compared with the control group, ^@@^ *p* < 0.01 compared with the IL-1β group, n = 3.

It was speculated that betulin has an anti-inflammatory effect in the OA cell model. To validate this hypothesis, it was first investigated whether betulin inhibits the production of pro-inflammatory mediators. The expression of iNOS and COX-2 was detected by Western blot (Figures 2A,B). The generation of iNOS and COX-2 stimulated by IL-1β was significantly higher than that in the control group (*p* < 0.01). Betulin treatment could reduce the expression of iNOS and COX-2 in the chondrocytes in a dose-dependent manner (*p* < 0.01). The cell suspension was collected using TRIzol reagent to analyze the mRNA levels of the pro-inflammatory mediators (IL-6 and TNF-α) and enzymes (iNOS and COX-2) by quantitative RT-PCR. The results further verify this conclusion (Figures 2C,D). In addition, released enzymes (iNOS and COX-2) generated NO and PGE2 inflammatory mediators in cells, respectively. Griess reaction was used to detect the concentration of endogenous NO in cell suspensions, and the ELISA kit was used to detect the levels of PGE2, TNF-α, and IL-6. As shown in Figures 2E,F, the expression of NO, IL-6, TNF-α, and PGE-2 increased significantly compared with that in the blank control group (*p* < 0.01), while betulin reversed this phenomenon in a dose-dependent manner. This verified that betulin could inhibit the generation of pro-inflammatory mediators.

### Betulin Inhibits the Degradation of Extracellular Matrix Induced by IL-1β

**FIGURE 3:**
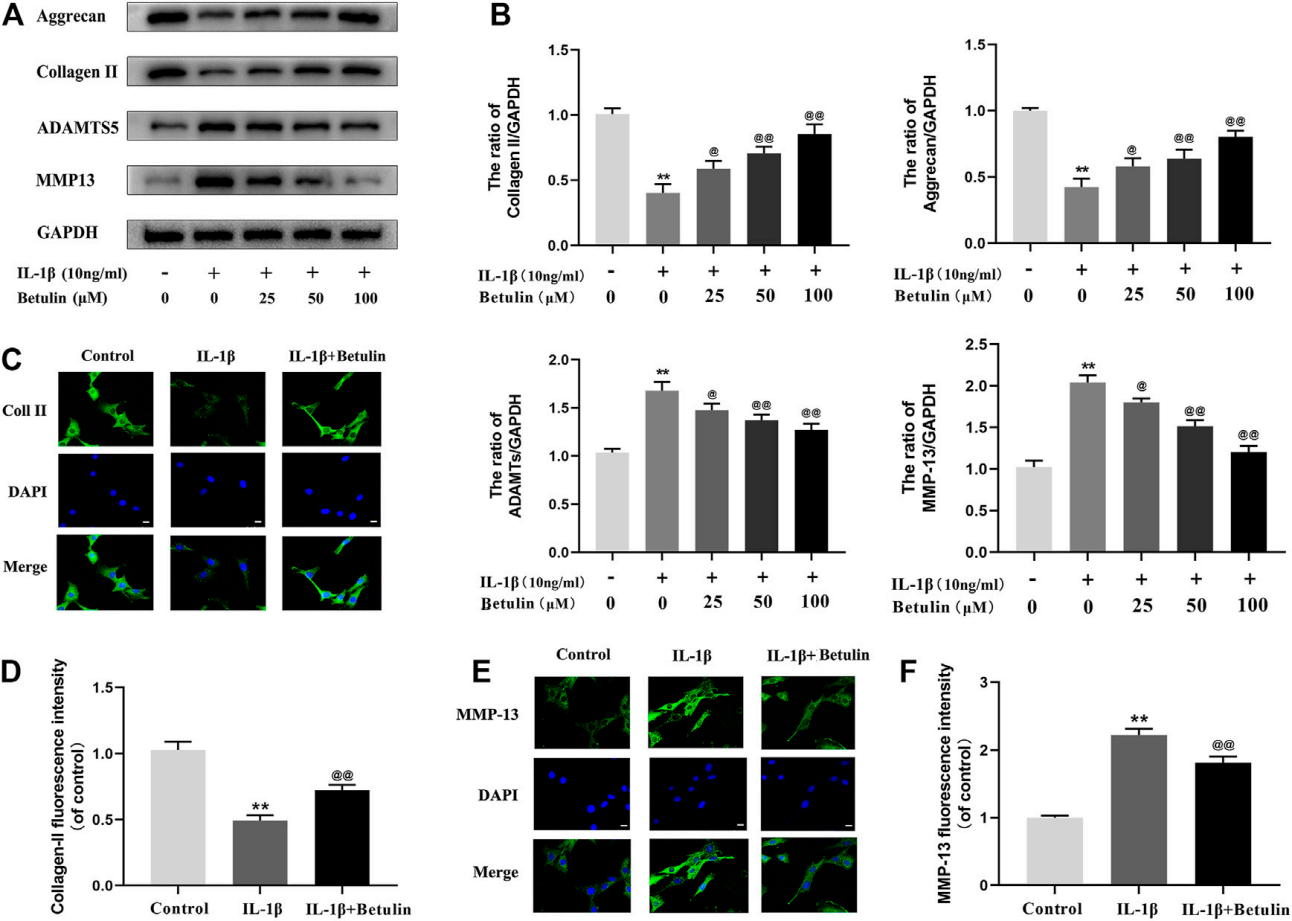
Betulin treatment can inhibit the degradation of extracellular matrix. OA chondrocytes were cultured with betulin (0, 25, 50, and 100 μM) of different concentrations for 2 h, followed by stimulation with or without IL-1β (10 ng/ml) for 24 h. **(A**–**B)** The protein expression levels of Collagen II, Aggrecan, MMP13, and ADAMTS-5 were detected by Western blot and quantitatively analyzed. **(C**–**F)** Immunofluorescence staining combined with DAPI staining (scale: 25 μm) was used to detect the fluorescent expression of MMP13.The data in the figure are represented as the mean ± SD, **p < 0.01 compared with the control group, ^@@^ *p* < 0.01 compared with the IL-1β group, n = 3.

In the progress of OA, the extracellular matrix of cartilage cells is continuously degraded, to explore the protective effect of betulin on cartilage extracellular matrix degradation induced by IL-1β. The expression of Aggrecan, Collagen II, ADAMTS-5, and MMP13 in chondrocytes was detected by Western blot with betulin of different concentrations (Figures 3A,B). The results showed that the expression of Collagen II and Aggrecan decreased significantly, while the expression of ADAMTS-5 and MMP13 increased significantly after the stimulation of IL-1β (*p* < 0.01). However, betulin reversed the expression of Collagen II and reduced the protein levels of MMP13 and ADAMTS5 in a dose-dependent manner. In addition, we also used the immunofluorescence method. After the treatment of IL-1β, the fluorescence intensity of Collagen II decreased while that of MMP-13 increased (*p* < 0.01). The treatment of betulin reversed this trend (Figures 3C–F) (*p* < 0.01), which conformed to that of Western blot, and further verified that betulin could prevent ECM degradation and suppress the development of OA.

### Betulin Inhibits NF-κB Signal Activation in OA Chondrocytes

**FIGURE 4:**
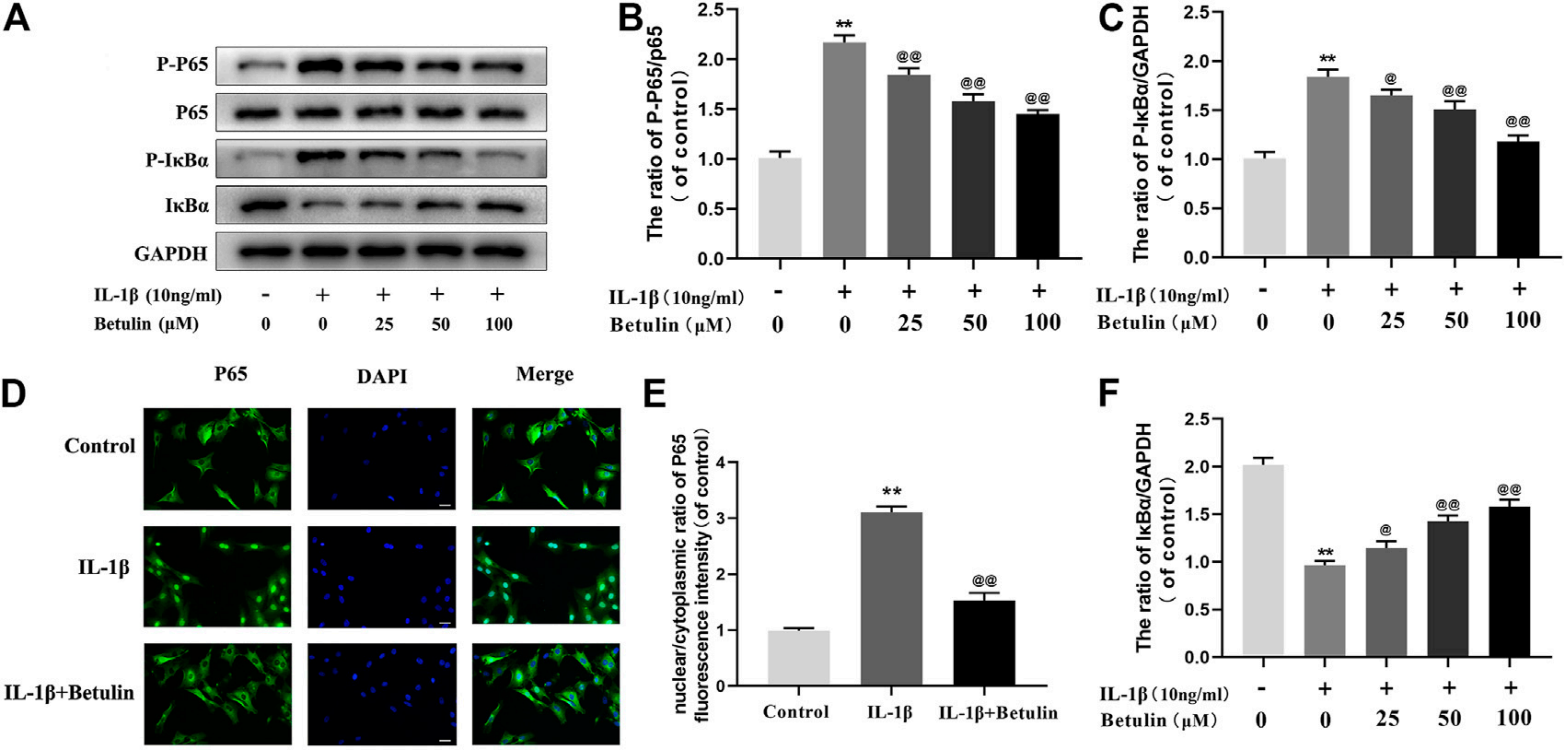
Betulin treatment inhibited the activation of NF-κB signal. OA chondrocytes were cultured with betulin (0, 25, 50, and 100 μM) of different concentrations for 2 h, and then stimulated by IL-1β (10 ng/ml) for 24 h. **(A**–**C**,**F**) The protein expression levels of p-p65, p65, IκBα, and p-IκBα were detected by Western blot and quantitatively analyzed. **(D**,**E)** Immunofluorescence staining combined with DAPI staining (scale: 25 μm) were used to detect the entry of characteristic index p65 into the nucleus and quantitative analysis of fluorescence intensity. The data in the figure are represented as the mean ± SD, **p < 0.01 compared with the control group, ^@@^ *p* < 0.01 compared with the IL-1β group, n = 3.

Many molecules involved in inflammatory reaction are regulated by the NF-κB pathway. To further explore the effect of betulin on the NF-κB pathway in chondrocytes, the expression of the target proteins in the NF-κB pathway induced by betulin was observed using Western blot (Figure 4A,B,C,F). The phosphorylation of IκBα and p65 increased significantly after IL-1β stimulation (*p* < 0.01). Betulin treatment could inhibit the degradation of IκBα in the cytoplasm and inhibit the phosphorylation of p65. Then, we further observed the effect of betulin on the translocation of p65 to IL-1β induced into the nucleus. As expected, in the control group, the p65 active protein was mainly located in the cytoplasm, while in the IL-1β group, most of the p65 active protein was transferred to the nucleus. The betulin pretreatment significantly inhibited p65 translocation to the nucleus (Figures 4D,E) (*p* < 0.01). These results suggest that betulin inhibits the activation of the NF-κB signal pathway in the chondrocytes and plays an anti-inflammatory role.

### Molecular Docking Between Betulin and AKT Protein

**FIGURE 5:**
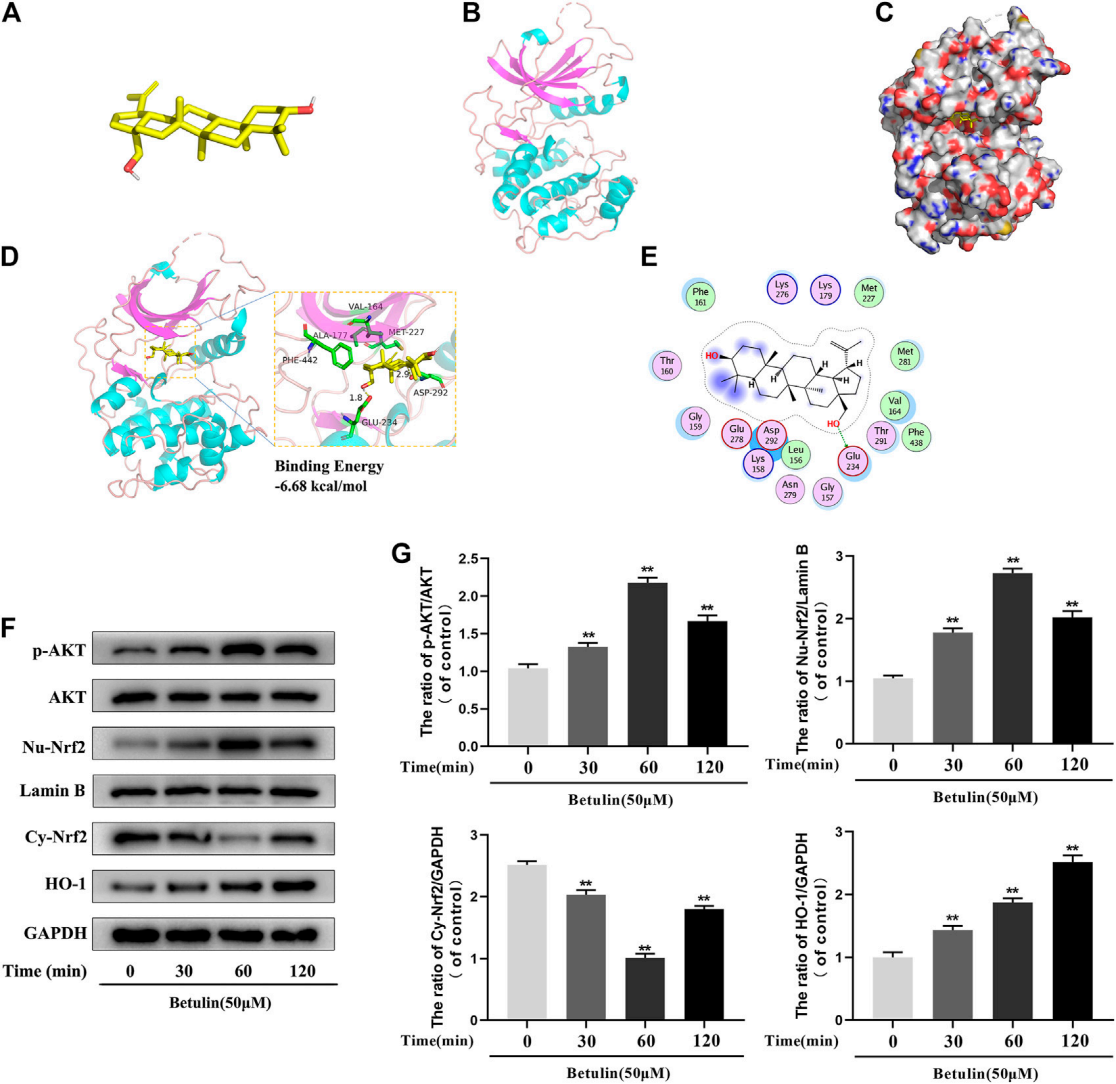
The molecular docking model shows the affinity between Betulin and AKT. Betulin promotes the phosphorylation of AKT, enhances the nuclear expression of Nrf2, and upregulates the expression of HO-1. **(A)** The model of Betulin. **(B)** The ribbon model of AKT. **(C)** The space filling model of the binding of Betulin and AKT protein. **(D)** Three-dimensional (3D) binding model between Betulin and AKT protein. The backbone of the protein was rendered in tube and appears bright blue; Betulin is rendered yellow; the yellow dash represents the hydrogen bond distance, and the binding energy of the two is −6.68 kcal/mol. **(E)** Two-dimensional binding model between Betulin and AKT protein. The chondrocytes were treated with betulin (50 μM) for 0, 30, 60, and 120 min. **(F**–**G)** The protein levels of nuclear Nrf2 (Nu-Nrf2), p-AKT, HO-1, and cytoplasmic Nrf2 (Cy-Nrf2) were detected by Western blotting and quantitatively analyzed. The data in the figure are represented as the mean ± SD, **p < 0.01 compared with the control group, n = 3.

According to the results of molecular docking obtained by the standard method, the results show that the compound has a good binding to the target protein, and the binding energy is −6.68 (kcal/mol). The lower the energy required for binding, the easier it is to bind to the target protein. In addition, the complex formed by the docking compound and protein was visualized by Pymol2.1 software, and the binding mode of the compound and protein was obtained (Figures 5A–C). According to the binding mode, we can clearly see that betulin and AKT protein bind to VAL-164, ALA-177, PHE-442, GLU-234, MET-227, ASP-292, and other amino acid residues. We also found that betulin can form strong hydrogen bonds with amino acid active groups such as GLU-234 and ASP-292, and the distances of 1.8 Å and 2.9 Å are much smaller than 3.5 Å of the traditional hydrogen bonds, which plays an important role in the stability of both. In addition, betulin can also form hydrophobic interactions with hydrophobic residues (Figures 5D,E). These interactions can improve the stability of betulin in AKT protein, indicating that betulin and AKT protein have a high affinity. Betulin is a potentially active small molecule that can be used to activate AKT.

### Betulin Promotes the Phosphorylation of AKT, Enhances the Nuclear Expression of Nrf2, and Upregulates the Expression of HO-1

Studies have shown that AKT, Nrf2, and HO-1 are involved in inflammation. In order to explore the influence of betulin on AKT, Nrf2, and HO-1 signaling pathways, the chondrocytes were treated with betulin at different time gradients. It was found that betulin promoted the phosphorylation of AKT and reached the highest at 60 min but decreased at 120 min. Furthermore, betulin activated Nrf2, and the expression of Nrf2 reached the highest in the nucleus of 60 min. There was a time delay in the activation of HO-1, which reached peak in 120 min (Figures 5F,G) (*p* < 0.01).

### Betulin Promotes Nuclear Translocation of Nrf2 via AKT Signal Pathway

**FIGURE 6:**
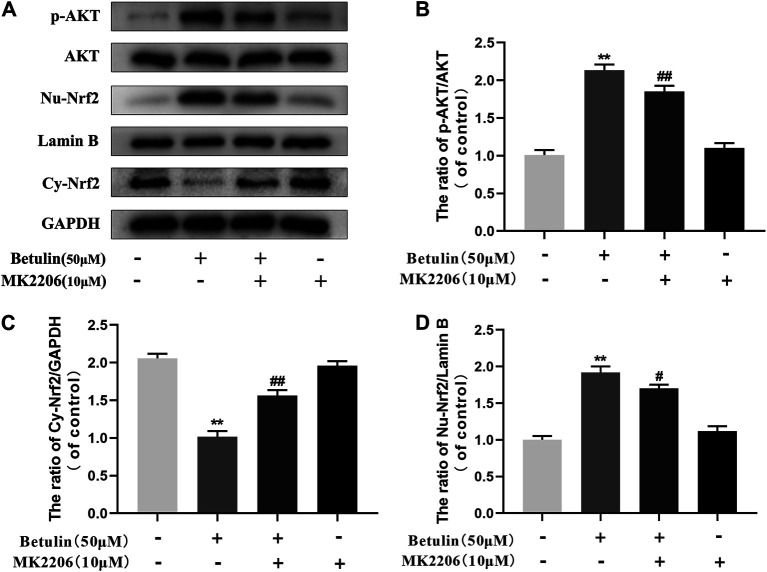
Betulin promotes nuclear translocation of Nrf2 via the AKT signal pathway. The chondrocytes were pretreated with AKT inhibitor MK2206 (10 μM) for 2 h and then treated with betulin (50 μM) for 2 h. **(A**–**D)** The protein levels of Nu-Nrf2, Cy-Nrf2, and p-AKT were detected by Western blotting and quantitatively analyzed. The results showed that the AKT signal participated in the inflammatory process induced by IL-1β. The data in the figure show that the average value is ±SD, **p < 0.01 compared with the control group, ^**##**^ *p* < 0.01 compared with the Betulin-treated group, n = 3.

Previous studies have revealed that the activation of Nrf2 is regulated by upstream kinases. To confirm that betulin regulates Nrf2 nuclear translocation via the AKT pathway, chondrocytes were pretreated with AKT inhibitor (MK2206, 10 μM), and then treated with betulin (50 μM). The expression of phosphorylated AKT, nuclear Nrf2 (Nu-Nrf2), and cytoplasmic Nrf2 (Cy-Nrf2) was detected by Western blot. The results showed that although betulin promoted the nuclear translocation of Nrf2 (*p* < 0.01), MK2206 inhibited the nuclear expression of Nrf2 (*p* < 0.05), indicating that betulin’s promotion of Nrf2 nuclear translocation is mediated by the AKT pathway (Figures 6A–D).

### Betulin Upregulates the Expression of HO-1 via Activating Nrf2 Pathway

**FIGURE 7:**
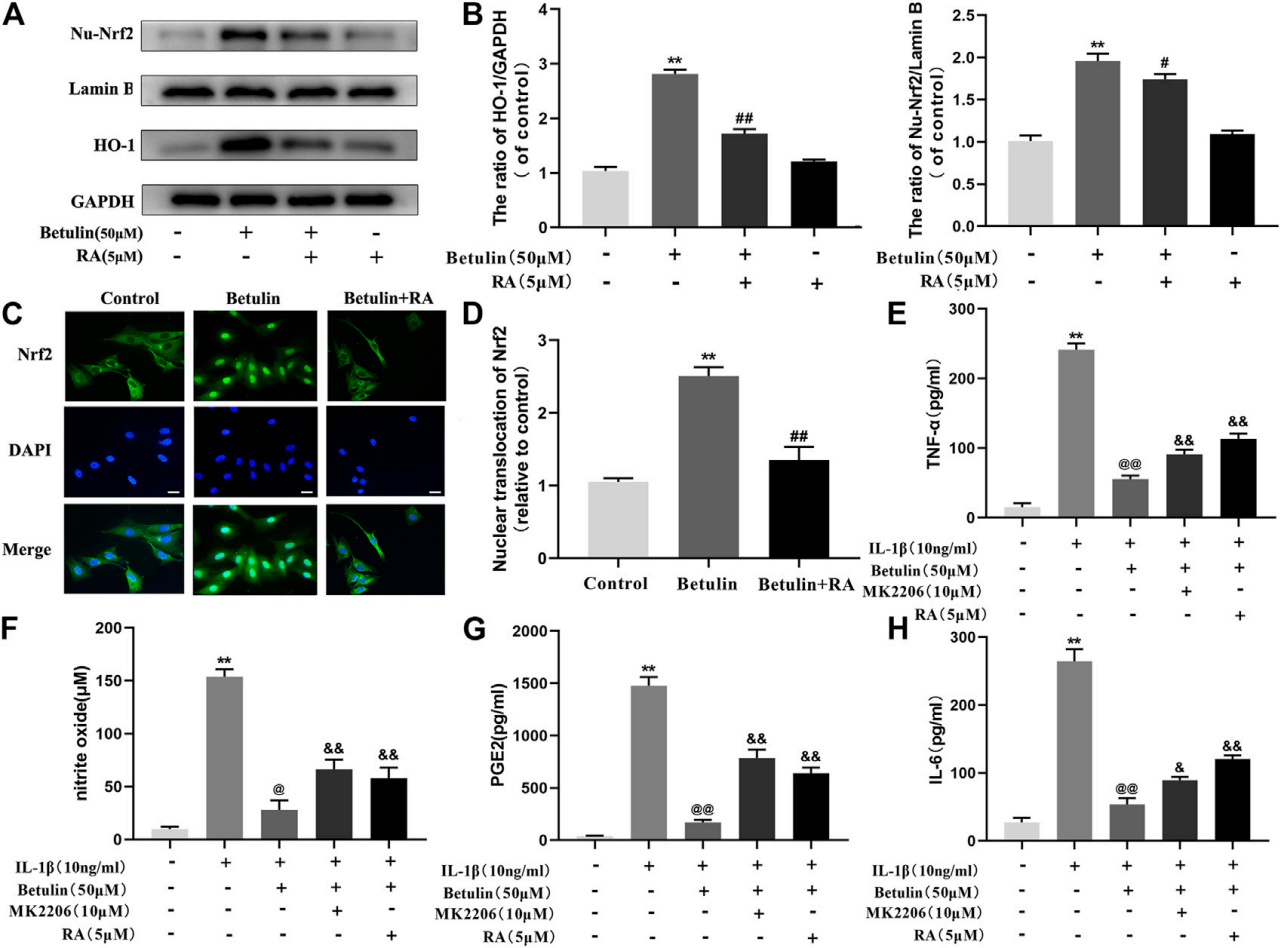
Betulin upregulates the expression of HO-1 via activating the Nrf2 pathway. The chondrocytes were treated with retinoic acid (RA, 5 μM), an inhibitor of Nrf2, for 2 h and then treated with betulin (50 μM) for 2 h. **(A**–**B)** The expression levels of Nu-Nrf2 and HO-1 in chondrocytes were detected by Western blotting and quantitatively analyzed. **(C**–**D)** Immunofluorescence staining combined with DAPI staining (scale: 25 μm) were used to detect the entry of Nrf2 into the nucleus and quantitatively analyzed. **(E**–**H)** The protein levels of TNF-α, IL-6, and PGE2 were measured by the commercial ELISA kits, and the generation of NO was measured by Griess reaction. The data in the figure show that the average value is ±SD, **p < 0.01 compared with the control group, ^**##**^ *p* < 0.01 compared with the Betulin-treated group, ^&&^ *p* < 0.01 compared with the Betulin + IL-1β–treated group, ^@^p < 0.05 compared with the IL-1β group, n = 3.

To illustrate the relationship between Nrf2 and HO-1, Chondrocytes were pretreated with Nrf2 inhibitor (RA, 5 μM), then treated with betulin (50 μM), and the protein levels of HO-1 and Nu-Nrf2 were detected by Western blot. The results showed that the protein levels of HO-1 and Nu-Nrf2 increased significantly after betulin treatment compared to the control group (*p* < 0.01) and Nrf2 transfer to the nucleus (Figures 7A–D) (*p* < 0.01). The inhibition of Nrf2 by RA abolished the upregulation of HO-1 protein induced by betulin and inhibited the nuclear translocation of Nrf2. These results indicate that betulin promotes HO-1 expression by activating the Nrf2 pathway. Furthermore, it was observed whether MK2206 and RA treatment affect the anti-inflammatory effect of betulin. The results showed that treatment with RA and MK2206 partially reversed the inhibitory effect of betulin on pro-inflammatory mediators (Figures 7E–H). These results indicate that betulin exerts anti-inflammatory effects through the AKT/Nrf2/HO-1 pathway.

### Betulin Inhibits the Activation of NF-κB Pathway and the Generation of Pro-inflammatory Mediators Through HO-1 Pathway

**FIGURE 8:**
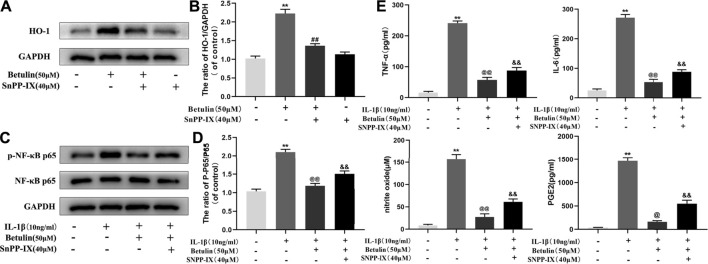
Betulin inhibits the activation of the NF-κB pathway and the generation of pro-inflammatory mediators through the HO-1 pathway. The chondrocytes were pretreated with HO-1 inhibitor SnPP-IX (40 μM) for 2 h and treated with betulin (50 μM) for 2 h. **(A**–**D)** The protein levels of p-p65 and HO-1 were detected by Western blotting and quantitatively analyzed. **(E)** The protein levels of TNF-α, IL-6, and PGE2 were measured by the commercial ELISA kits and the generation of NO was measured by Griess reaction. The data in the figure show that the average value is ±SD, **p < 0.01 compared with the control group, ^**##**^ *p* < 0.01 compared with the Betulin-treated group, ^&&^ *p* < 0.01 compared with the Betulin + IL-1β–treated group, ^@^ *p* < 0.05, ^@@^ *p* < 0.01 compared with the IL-1β group, n = 3.

Studies have shown that HO-1 can regulate the NF-κB pathway. The chondrocytes were pretreated with HO-1 inhibitor SnPP-IX (40 μM) and then treated with betulin (50 μM). The results of Western blot showed that SnPP-IX pretreatment abolished the inhibitory effect of betulin on p65 phosphorylation (Figures 8A–D) (*p* < 0.01). In addition, we also observed whether the HO-1 inhibitor SnPP-IX affected the anti-inflammatory effect of betulin. The results showed that SnPP-IX treatment reversed the inhibitory effect of betulin on pro-inflammatory mediators (Figure 8E) (*p* < 0.01). These results suggest that betulin plays an anti-inflammatory role by inhibiting the activation of the NF-κB pathway in chondrocytes through the HO-1 pathway.

### Betulin Plays a Protective Role in the DMM OA Model

**FIGURE 9:**
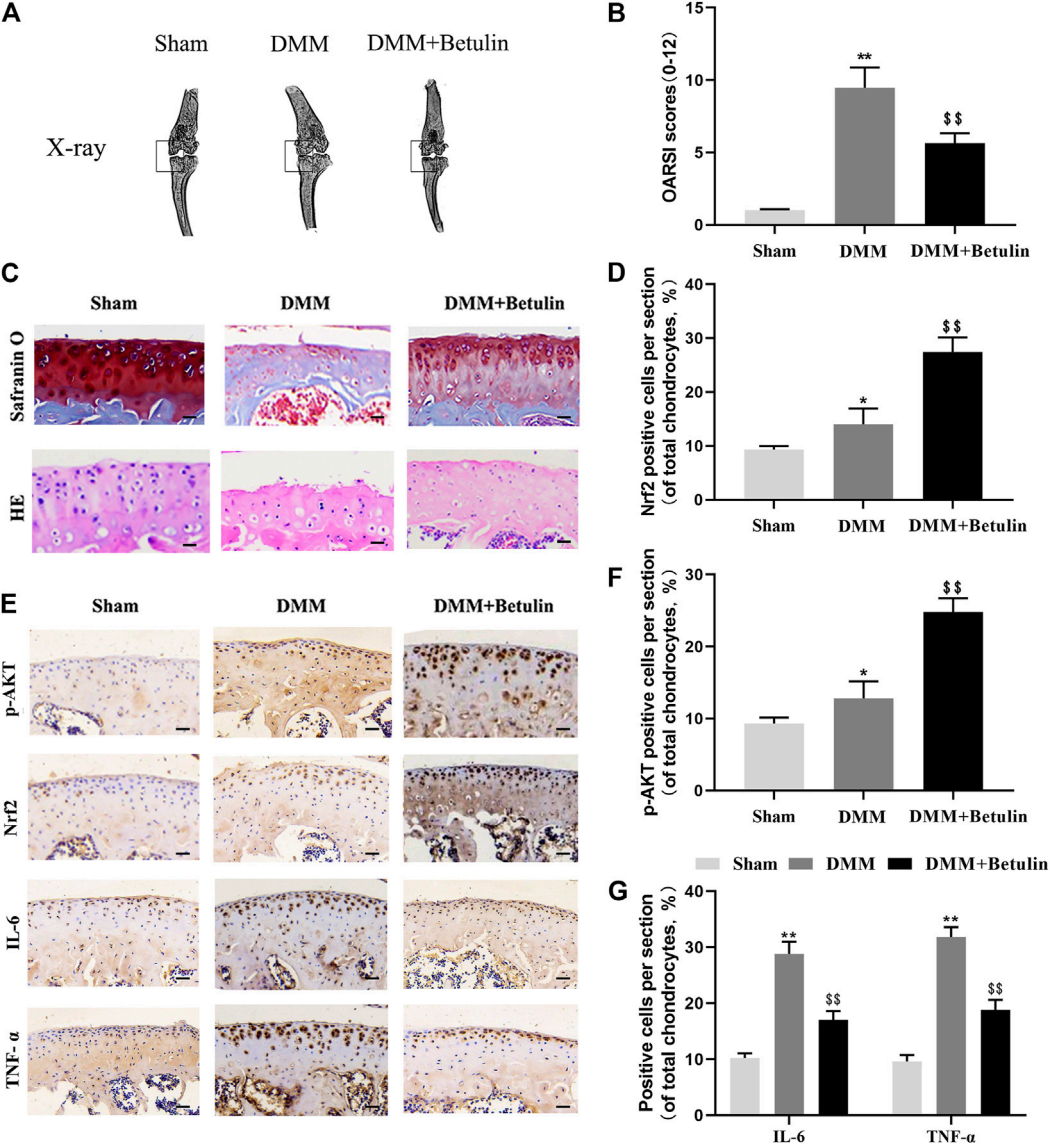
Betulin inhibits the progression of DMM-induced OA in mice. The mice were randomly divided into three groups: the Sham group, DMM group, and DMM + Betulin group. The sham operation was performed in the Sham group, and the OA model was established in the DMM and DMM + Betulin groups. Eight weeks after operation, **(A)** X-ray imaging of the knee joints of the mice in the different experimental groups were performed, and betulin processing alleviated the stenosis of the joint space. **(B**–**C)** The knee joints of the mice in the different experimental groups were stained with SO staining and HE staining (scale: 50 μm), and the histological changes of OA were evaluated by the score of the OARSI. **(E)** Immunohistochemical staining of p-AKT, Nrf2, IL-6, and TNF-α expression in the cartilage specimens (scale: 50 μm). **(D**–**G)** The percentage of p-AKT, Nrf2, IL-6, and TNF-α positive cells in each section was quantitatively analyzed by Image-Pro Plus software. The data in the figure show that the average value is ±SD, **p < 0.01 compared with the control group, ^$$^ *p* < 0.01 compared with the DMM group, n = 6.

We used the mouse DMM model induced by surgery. X-ray analysis, SO staining, HE staining, and the OARSI score were used to evaluate the severity of OA at 8 weeks after surgery. X-ray analysis showed that the joint space became narrower, and there existed hyperosteogeny in the DMM group, but this symptom was alleviated after the treatment of betulin (Figure 9A) (*p* < 0.01). SO staining and HE staining showed that there were more signs of destructive cartilage erosion on the articular cartilage surface in the DMM group than in the sham operation group (*p* < 0.05). The articular cartilage matrix was lost and a large amount of proteoglycan was degraded in the operation group. After betulin treatment, the cartilage matrix and cartilage thickness were alleviated, indicating that betulin thickened the articular cartilage and repaired the destruction of the articular cartilage (Figure 9C). The OARSI score was consistent with the above SO staining results (Figure 9B). Compared with the DMM group, the OARSI score decreased after betulin treatment (*p* < 0.01). The immunohistochemical staining of Nrf2 and p-AKT was used to further verify the effect of betulin on OA. As shown in the figure, the positive expression points of Nrf2 and p-AKT in the chondrocytes increased after betulin treatment (Figures 9D,F) (*p* < 0.01). In addition, we also performed immunohistochemical staining of inflammatory factors IL-6 and TNF-α. The results showed that the positive expression points of IL-6 and TNF-α in the chondrocytes decreased after betulin treatment, indicating that betulin inhibited the progression of inflammation in OA (Figure 9G) (*p* < 0.01). To sum up, Betulin inhibits the progression of OA in mice by activating the AKT/Nrf2/HO-1 signal axis *in vivo*.

## Discussion

The current research results show that betulin plays an anti-inflammatory role in the chondrocytes by activating the AKT/NRF-2/HO-1 pathway and inhibits the generation of pro-inflammatory cytokines (IL-6 and TNF-α) and enzymes (iNOS and COX-2) by inhibiting the NF-κB pathway, indicating that betulin is a potential candidate drug for the treatment of OA.

OA is one of world's most common degenerative diseases, especially among the elderly (Thysen et al., 2015). Existing evidence shows that inflammatory mechanism plays an important role in regulating biomechanical disorders of the joint tissue, resulting in OA occurrence and development (Wojdasiewicz et al., 2014). The subchondral bone as the source of inflammatory mediators participates in the pain process of OA. However, there is no effective drug treatment for OA (da Costa et al., 2017). Despite the wide application of nonsteroidal anti-inflammatory drugs in clinics (Sun et al., 2017), these can only temporarily relieve clinical symptoms, while their overuse will lead to serious adverse reactions. Therefore, a safe and effective drug with few side effects is needed. In recent years, plant-derived compounds have been provided to have good anti-inflammatory effects with less side reactions. They have attracted researchers' interest as drugs for the treatment of OA, among which betulin is one. It has biological activity including anti-inflammation (Ci et al., 2017; Ra et al., 2017). Therefore, this experiment mainly verifies whether betulin has a protective effect on OA. Through this experiment, we have discovered that betulin can inhibit the release of pro-inflammatory factors and inhibit the degradation of extracellular matrix of chondrocytes. In addition, we have also explored the mechanism of betulin in OA. Betulin inhibits the development of inflammation mainly by activating the AKT/Nrf2/HO-1 pathway and inhibiting the NF-κB pathway. To sum up, betulin can improve the injury of the articular cartilage and inhibit the progression of OA.

NF-κB is a nuclear transcription activator under research in recent years, with significant effect on the generation of inflammatory cytokines (Chun et al., 2014; Srinivasan and Lahiri, 2015). As it regulates the expression of inflammatory mediators, it is a target for the treatment of inflammatory diseases. Under physiological conditions, the NF-κB dimer binds to IκBα (inhibitor protein) in a state of inactivation. When stimulated, IκBα is degraded by phosphorylation, and p65 phosphorylation is transferred to the nucleus, inducing the overexpression of various inflammatory factors and aggravating the development of inflammation. It is reported that inhibiting the expression of p65 can block the expression of inflammatory mediators in chondrocytes stimulated by IL-1β (Hou et al., 2017). Therefore, in this experiment, we discussed the relationship between betulin and NF-κB signaling, betulin could inhibit the phosphorylation of NF-κB p65 and its translocation to the nucleus, thereby downregulating the expression and secretion of pro-inflammatory cytokines to play an anti-inflammatory effect.

AKT is a serine/threonine kinase and a downstream signal molecule of PI3K, activated by it (Zhang et al., 2015; Li et al., 2017; Ouyang et al., 2017). It regulates the release of inflammatory cytokines and plays an important role in various disease models as verified by studies (Yan et al., 2018). Thus, we speculate whether betulin also inhibits the progression of OA by activating AKT phosphorylation. As we expected, the phosphorylated expression of AKT was enhanced and the expression of Nrf2 in the nucleus was activated under the effect of betulin, indicating that AKT participated in the anti-inflammatory process of betulin. In addition, a large number of studies have shown that Nrf2 is a redox transcription factor and a major participant in antioxidant response (Motohashi and Yamamoto, 2004). PI3K can also promote neuronal survival by activating Akt phosphorylation and Nrf2 nuclear translocation as reported (de Oliveira et al., 2016). This experiment also proved that betulin-induced Nrf2 nuclear translocation was inhibited after being pretreated with MK2206 (AKT inhibitor), suggesting that the activation of the AKT pathway can increase Nrf2 nuclear translocation and enhance Nrf2 expression of antioxidant and anti-inflammatory genes in the nucleus, thus playing an anti-inflammatory role. To sum up, betulin exerts its anti-inflammatory effect by activating the AKT pathway to promote nuclear transfer of Nrf2.

**FIGURE 10:**
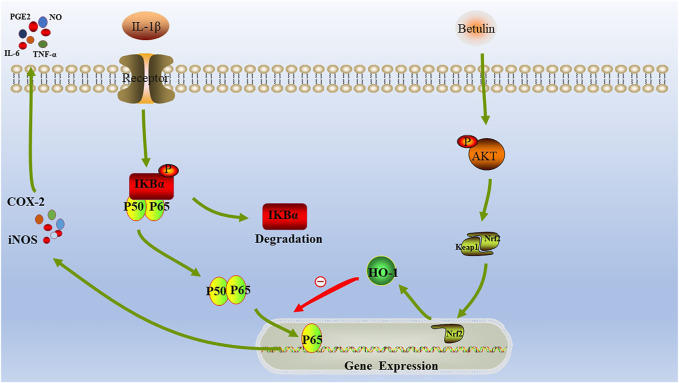
Schematic diagram: The molecular mechanism of betulin involved in the progress of OA.

HO-1 is the most important antioxidant enzyme *in vivo* with anti-inflammatory effects (Poss and Tonegawa, 1997; Yachie et al., 1999). Some studies have revealed that HO-1 is regulated by the Nrf2 signal pathway (Chen et al., 2003; Pittalà et al., 2013). Under physiological conditions, Nrf2 maintains its stability through the combining with Keap1 (Jaiswal, 2004). When stimulated, after dissociation from Keap1, Nrf2 enters the nucleus and binds to antioxidant response elements, resulting in the upregulation of downstream genes, such as HO-1 (Nguyen et al., 2009). In this study, we found that betulin activated the AKT/Nrf2/HO-1 signaling pathway. In addition, we also found that the pretreatment with HO-1 inhibitors (SnPP-IX) could inhibit the activation of NF-κB signal induced by IL-1β and could reverse betulin's anti-inflammatory effect, indicating that betulin plays an anti-inflammatory effect by activating the AKT/Nrf2/HO-1/NF-κB signal pathway. In addition, this conclusion was further confirmed by *in vivo* experiments. In short, Betulin may serve as therapeutic target for OA (Figure 10).

In summary, these results suggest that betulin significantly inhibits the inflammatory reaction of OA induced by IL-1β by activating the AKT/Nrf2/HO-1/NF-κB signal axis, further indicating that betulin, with significant anti-inflammatory effects, can serve as a safe and effective natural medicine for the treatment of OA.

## Data Availability

The original contributions presented in the study are included in the article/Supplementary Files; further inquiries can be directed to the corresponding authors.

## Abbreviations

AKT, protein kinase B; Nrf2, nuclear factor erythroid 2-related factor; HO-1, heme oxygenase-1; NF-κB, nuclear transcription factor-κB; SO, safranin O and fast green; HE, hematoxylin and eosinn; OARSI, Osteoarthritis Research Society International
