# Supplementary figures and images for: Betulin Alleviates the Inflammatory Response in Mouse Chondrocytes and Ameliorates Osteoarthritis via AKT/Nrf2/HO-1/NF-κB Axis
Source: Front Pharmacol. 2021 Oct 13;12:754038. doi: 10.3389/fphar.2021.754038 (PMC8548689; doi:10.3389/fphar.2021.754038)

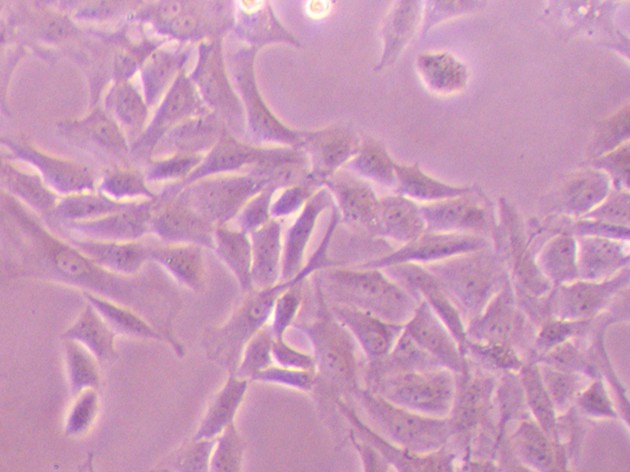

Supplement: Supplementary file 1 [file DataSheet1.zip › figure1/fig1-D cell viability/control.jpg]

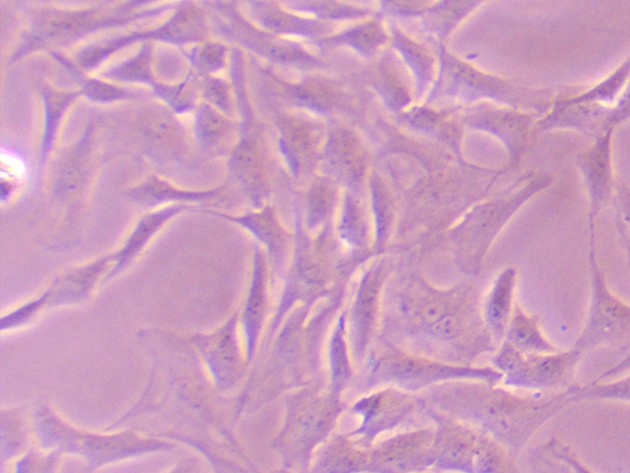

Supplement: Supplementary file 1 [file DataSheet1.zip › figure1/fig1-D cell viability/IL-1β+betulin100μM.jpg]

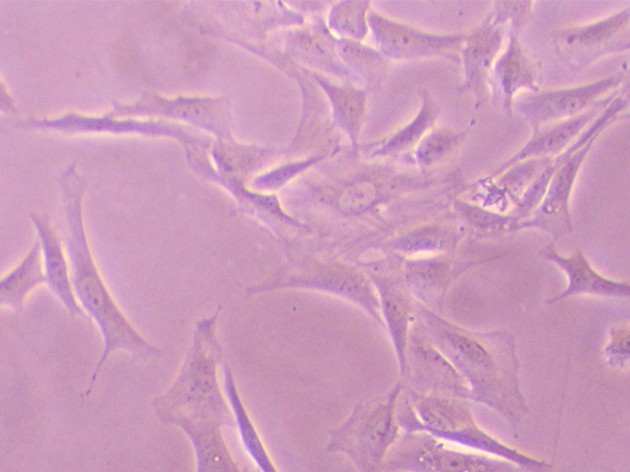

Supplement: Supplementary file 1 [file DataSheet1.zip › figure1/fig1-D cell viability/IL-1β+betulin25μM.jpg]

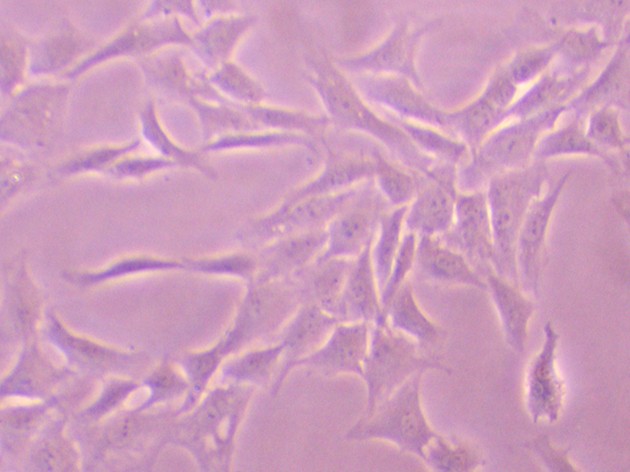

Supplement: Supplementary file 1 [file DataSheet1.zip › figure1/fig1-D cell viability/IL-1β+betulin50μM.jpg]

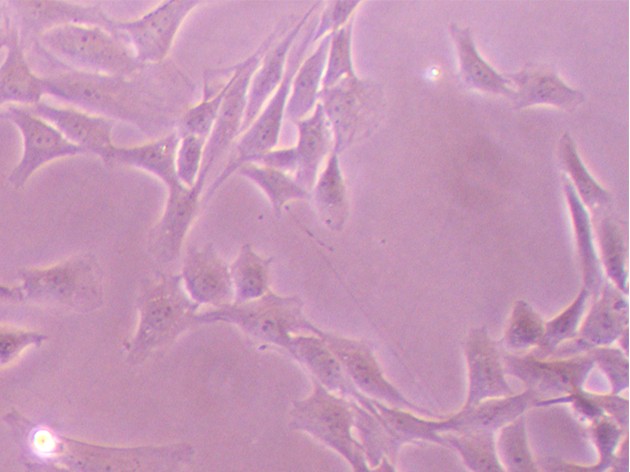

Supplement: Supplementary file 1 [file DataSheet1.zip › figure1/fig1-D cell viability/IL-1β.jpg]

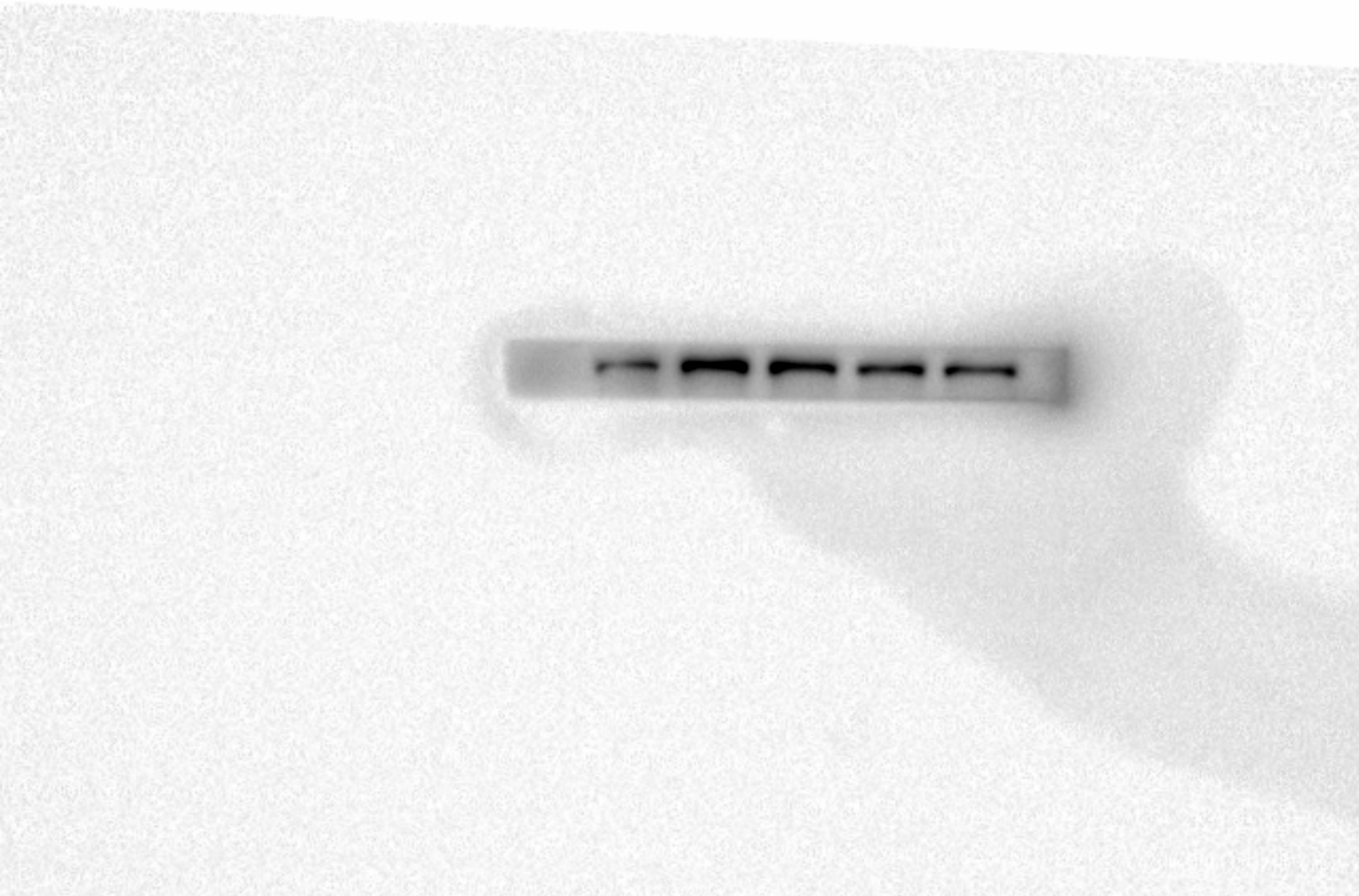

Supplement: Supplementary file 1 [file DataSheet1.zip › figure2/fig2-A western bolt/cox-2.jpg]

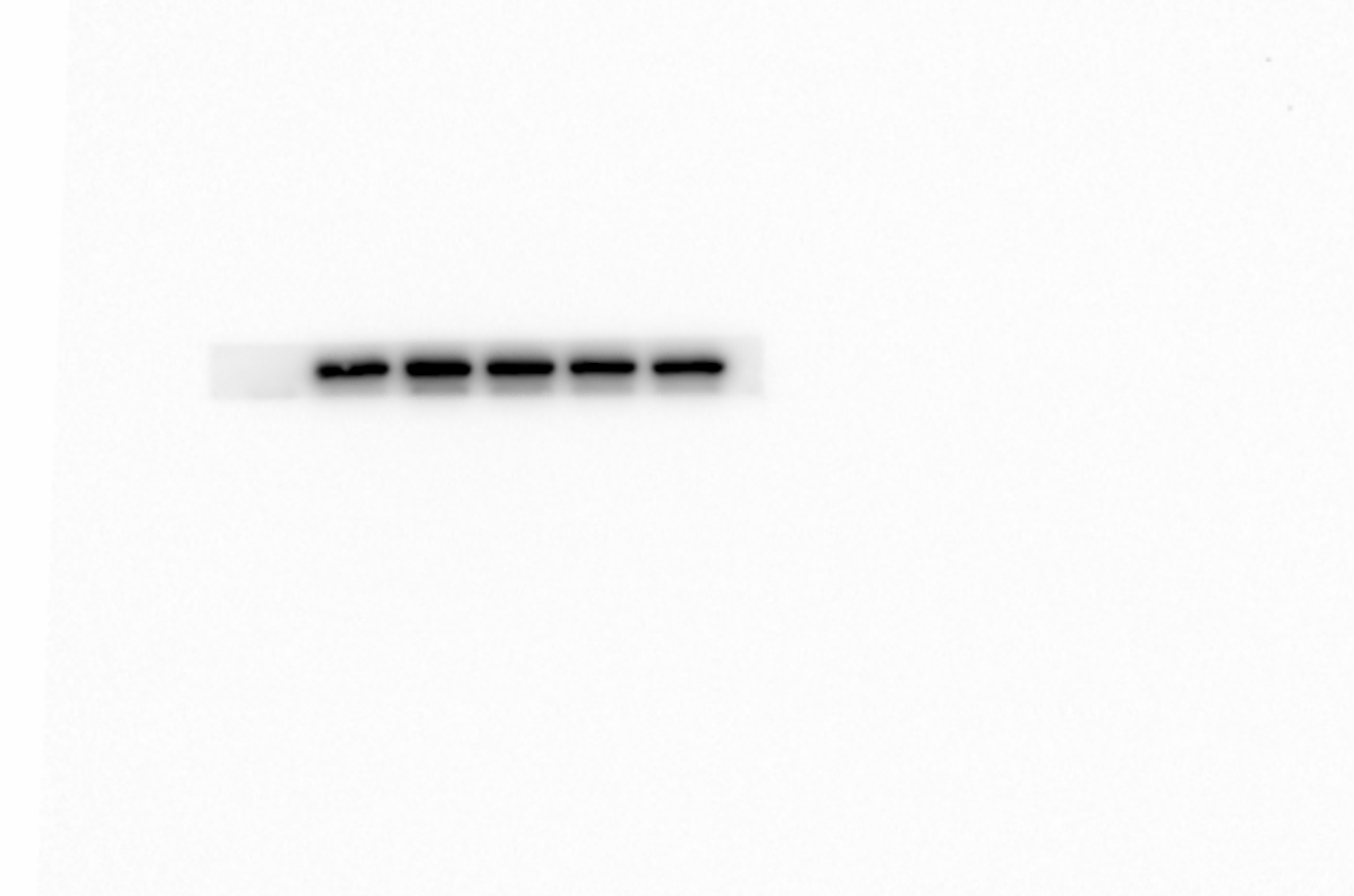

Supplement: Supplementary file 1 [file DataSheet1.zip › figure2/fig2-A western bolt/GAPDH.png]

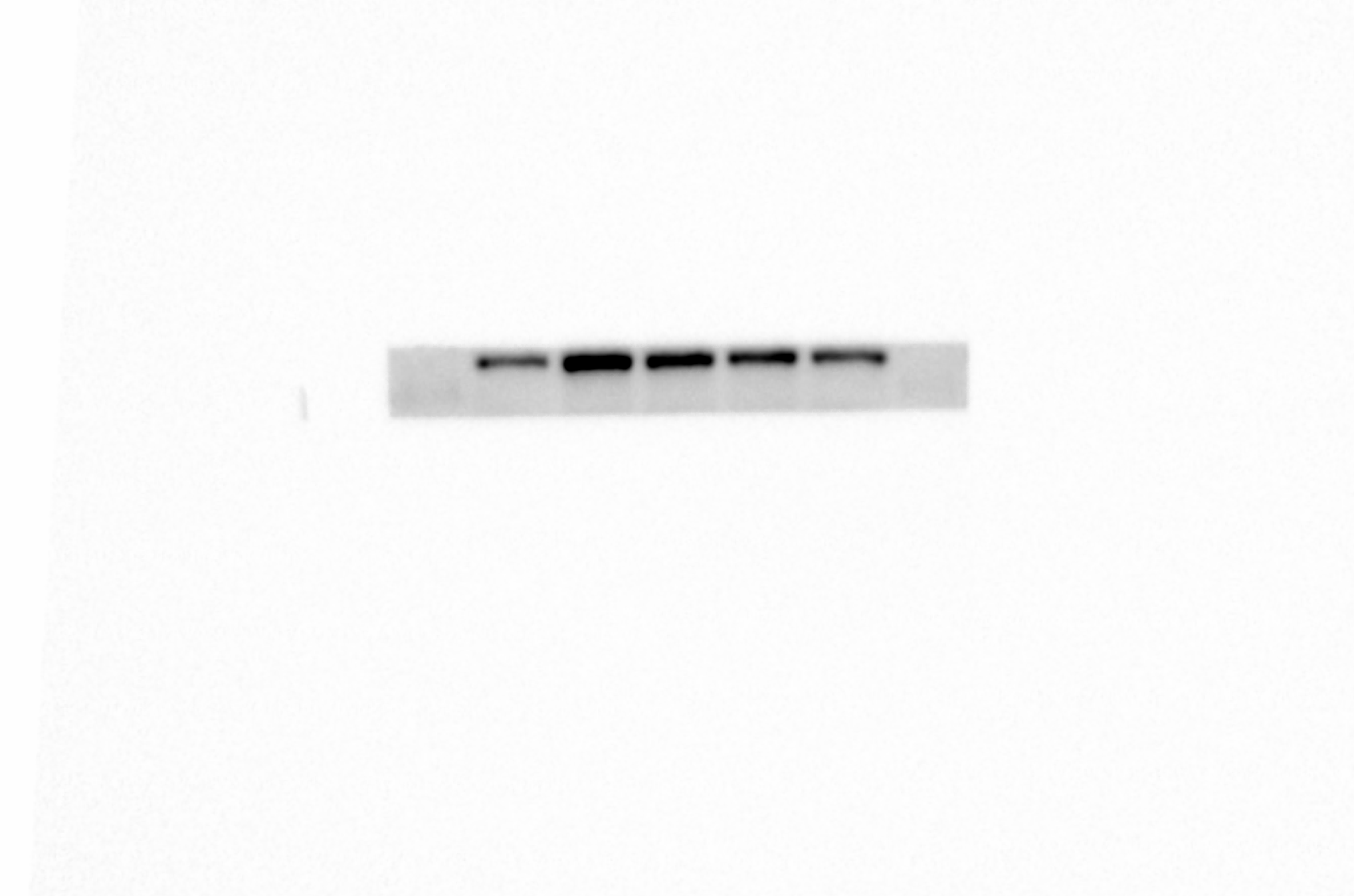

Supplement: Supplementary file 1 [file DataSheet1.zip › figure2/fig2-A western bolt/INOS.png]

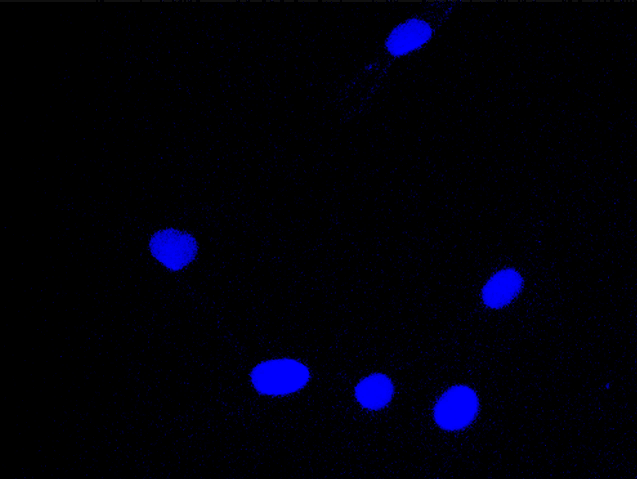

Supplement: Supplementary file 1 [file DataSheet1.zip › figure3/Immunofluorescence/Fig3-C Coll II/Betulin+IL-1β +dapi.png]

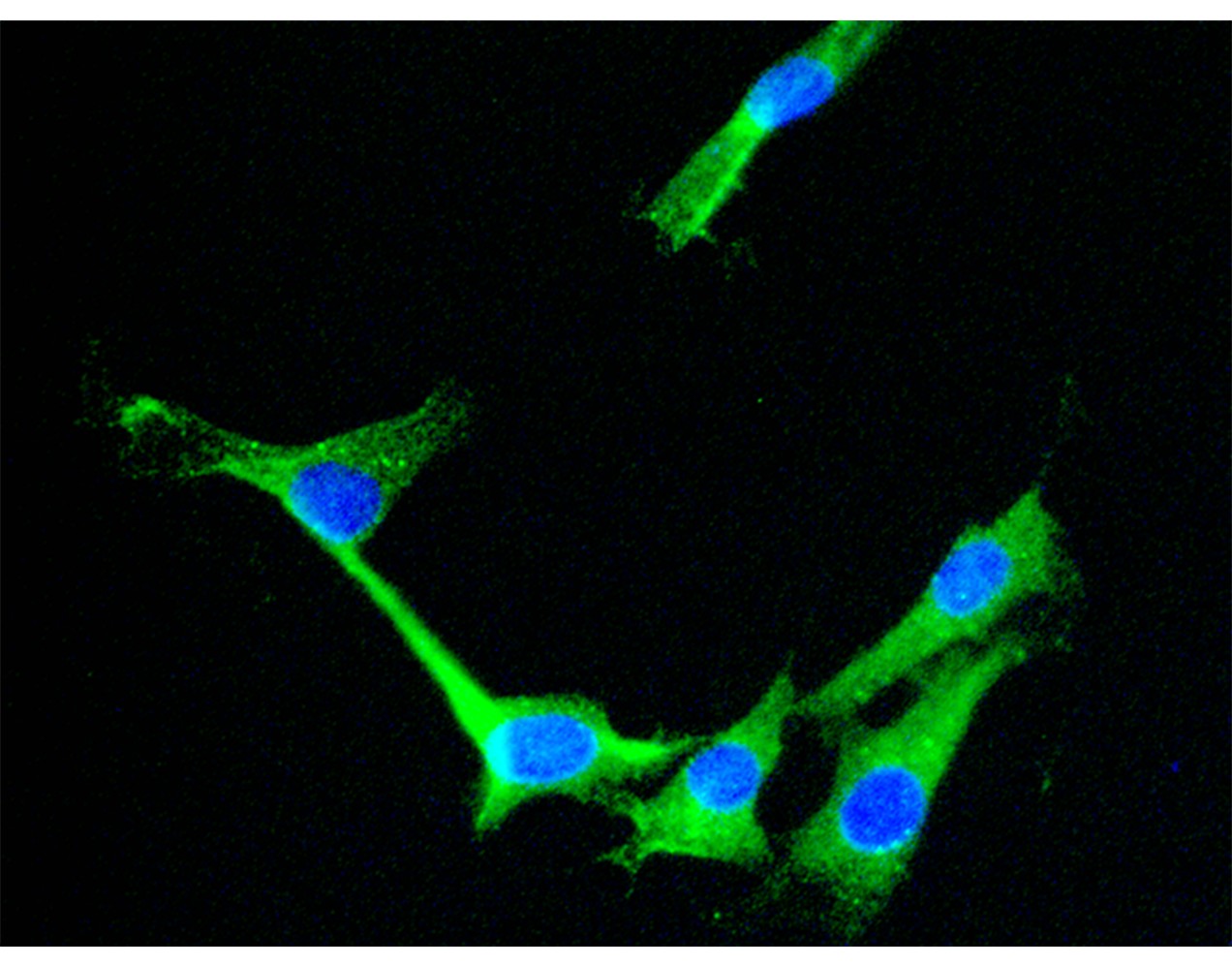

Supplement: Supplementary file 1 [file DataSheet1.zip › figure3/Immunofluorescence/Fig3-C Coll II/Betulin+IL-1β +merge.jpg]

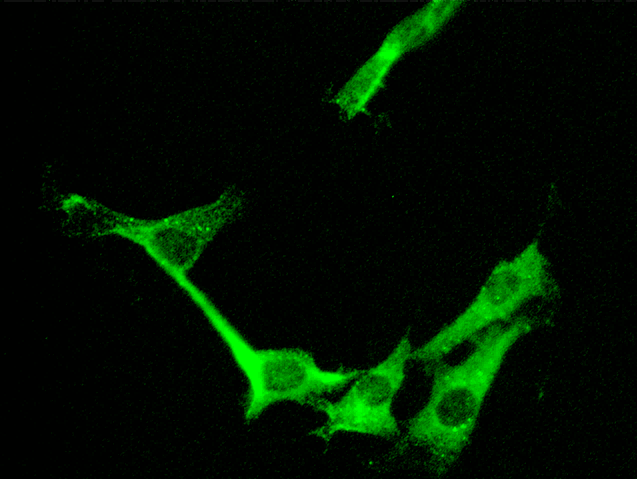

Supplement: Supplementary file 1 [file DataSheet1.zip › figure3/Immunofluorescence/Fig3-C Coll II/Betulin+IL-1β-coll.png]

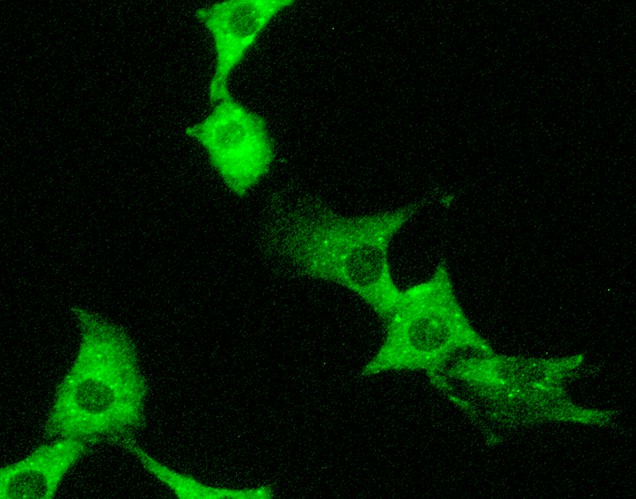

Supplement: Supplementary file 1 [file DataSheet1.zip › figure3/Immunofluorescence/Fig3-C Coll II/control-coll.jpg]

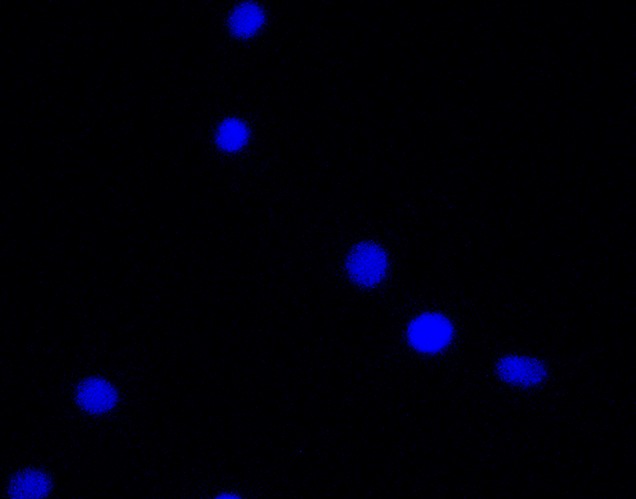

Supplement: Supplementary file 1 [file DataSheet1.zip › figure3/Immunofluorescence/Fig3-C Coll II/control-dapi.jpg]

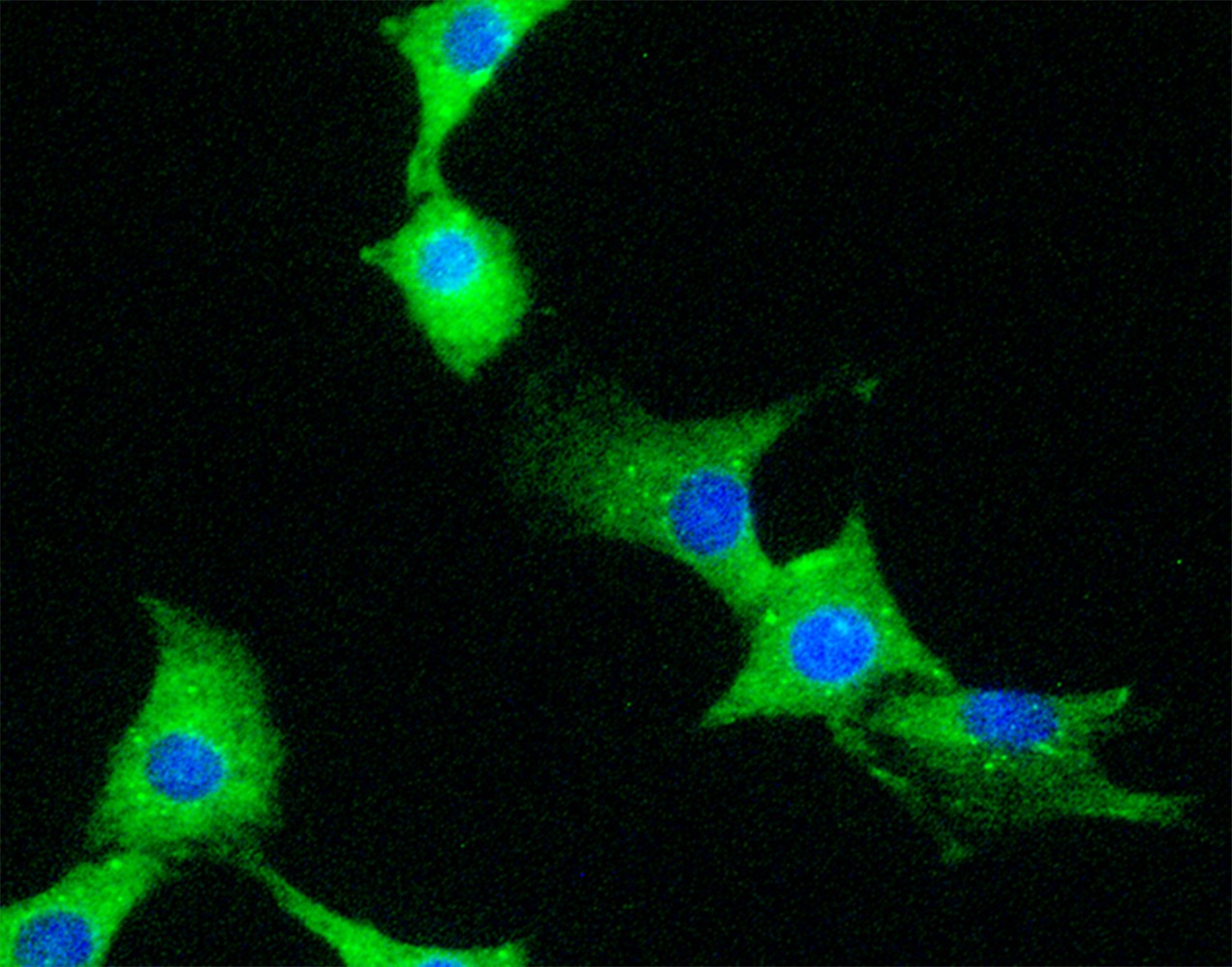

Supplement: Supplementary file 1 [file DataSheet1.zip › figure3/Immunofluorescence/Fig3-C Coll II/control-merge.jpg]

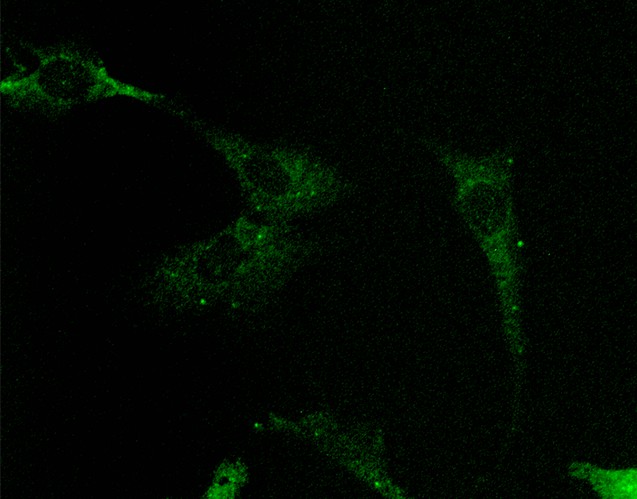

Supplement: Supplementary file 1 [file DataSheet1.zip › figure3/Immunofluorescence/Fig3-C Coll II/IL-1β-coll.jpg]

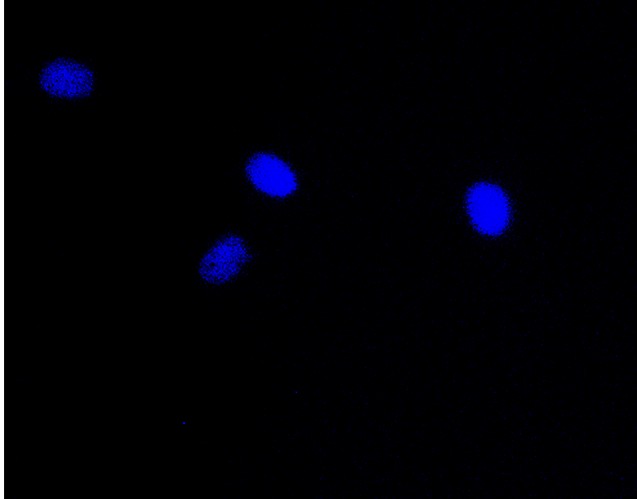

Supplement: Supplementary file 1 [file DataSheet1.zip › figure3/Immunofluorescence/Fig3-C Coll II/IL-1β-dapi.jpg]

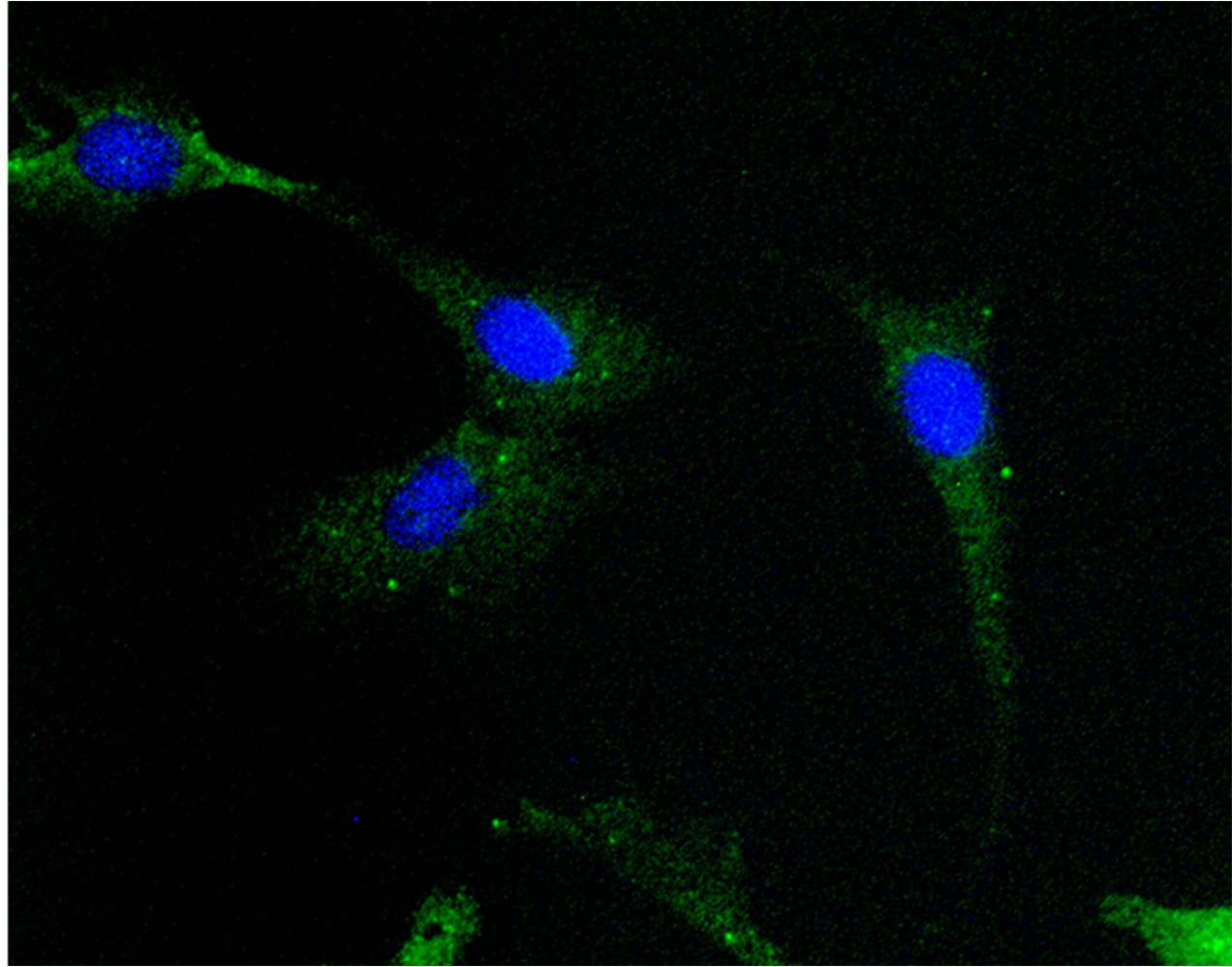

Supplement: Supplementary file 1 [file DataSheet1.zip › figure3/Immunofluorescence/Fig3-C Coll II/IL-1β-merge.jpg]

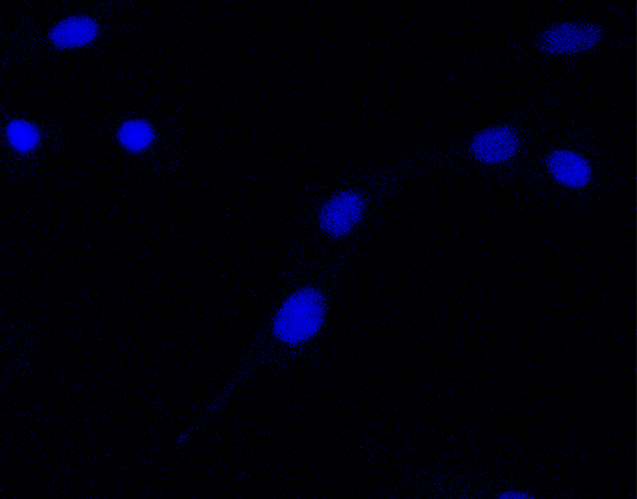

Supplement: Supplementary file 1 [file DataSheet1.zip › figure3/Immunofluorescence/Fig3-E MMP-13/Betulin+IL-1β-DAPI.png]

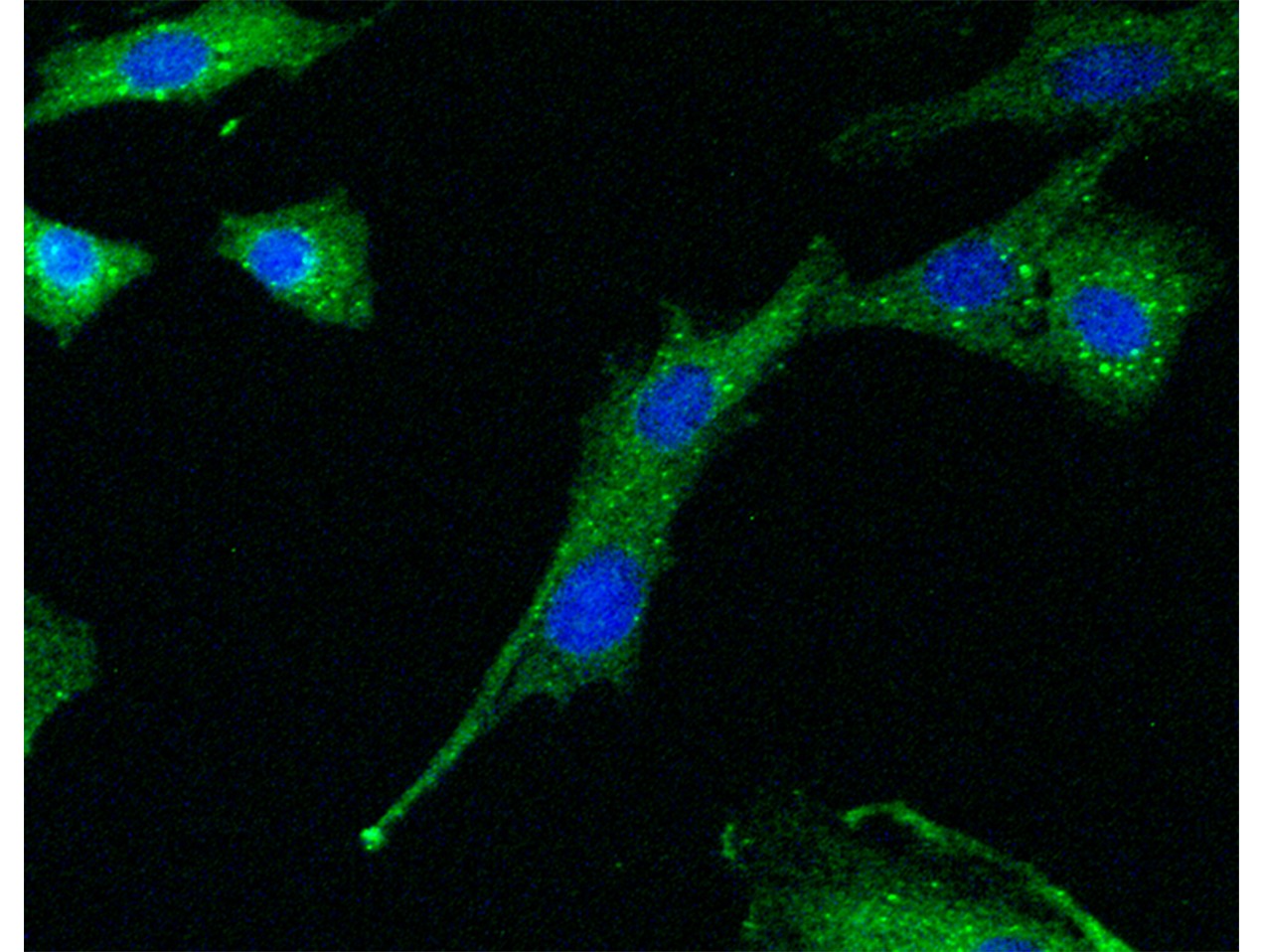

Supplement: Supplementary file 1 [file DataSheet1.zip › figure3/Immunofluorescence/Fig3-E MMP-13/Betulin+IL-1β-Merge.jpg]

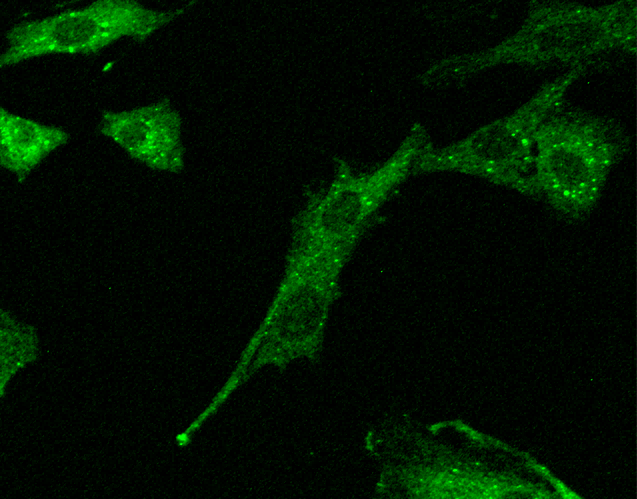

Supplement: Supplementary file 1 [file DataSheet1.zip › figure3/Immunofluorescence/Fig3-E MMP-13/Betulin+IL-1β-MMP13.png]

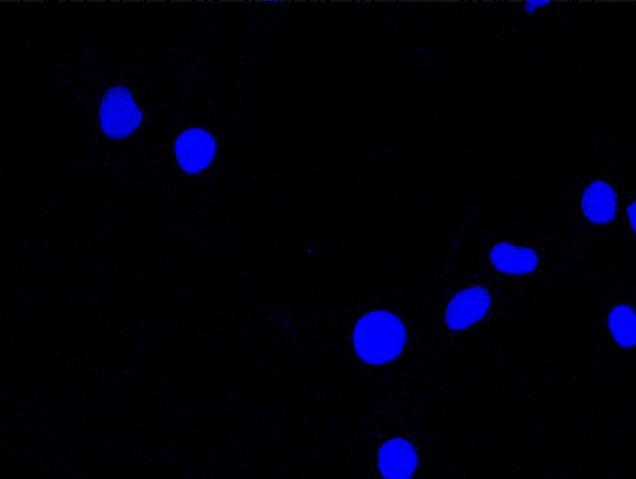

Supplement: Supplementary file 1 [file DataSheet1.zip › figure3/Immunofluorescence/Fig3-E MMP-13/control-DAPI.png]

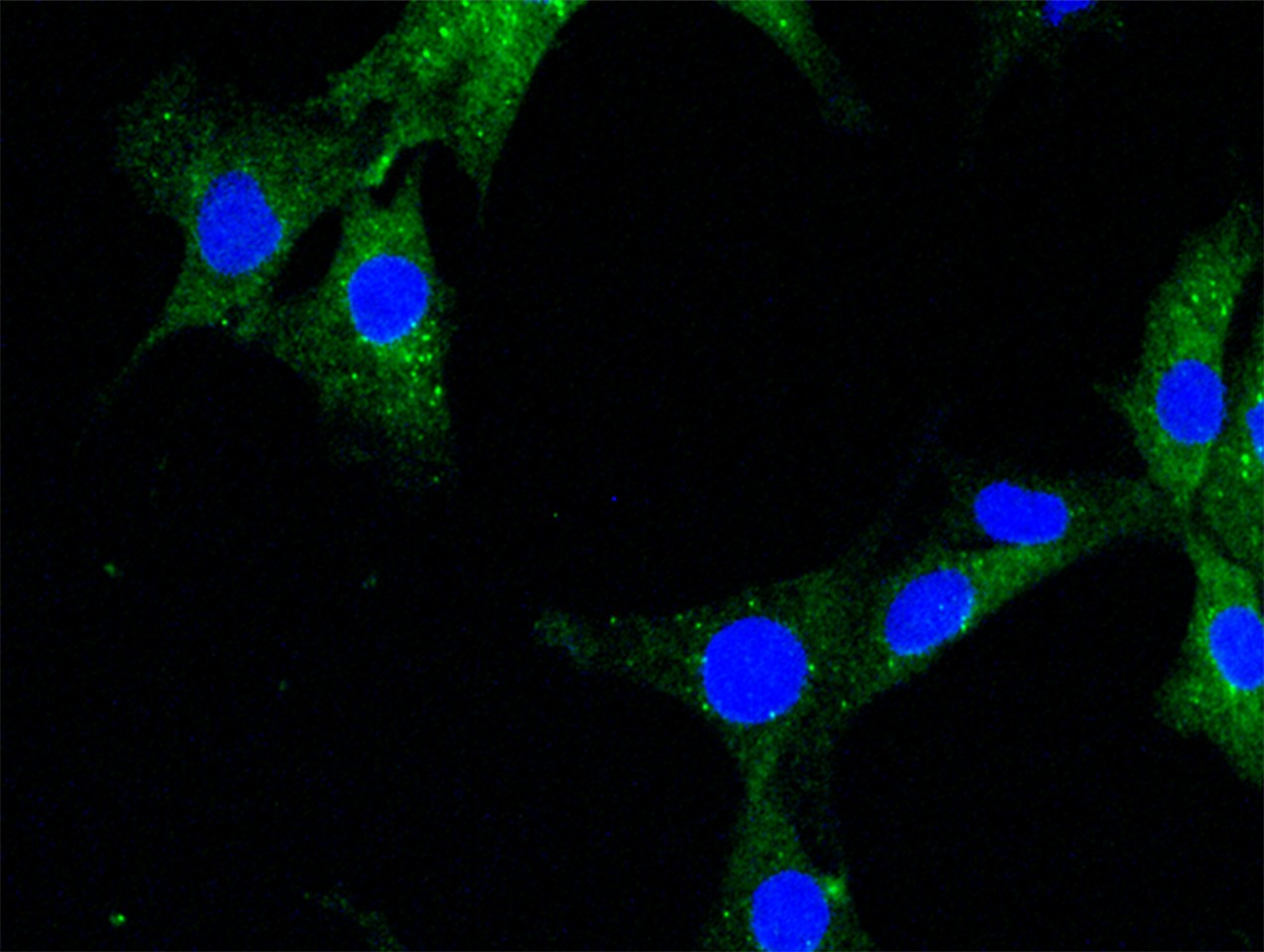

Supplement: Supplementary file 1 [file DataSheet1.zip › figure3/Immunofluorescence/Fig3-E MMP-13/control-Merge.jpg]

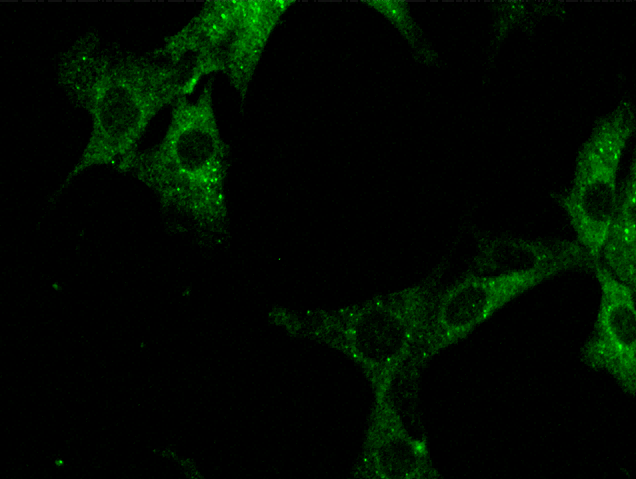

Supplement: Supplementary file 1 [file DataSheet1.zip › figure3/Immunofluorescence/Fig3-E MMP-13/control-MMP-13.png]

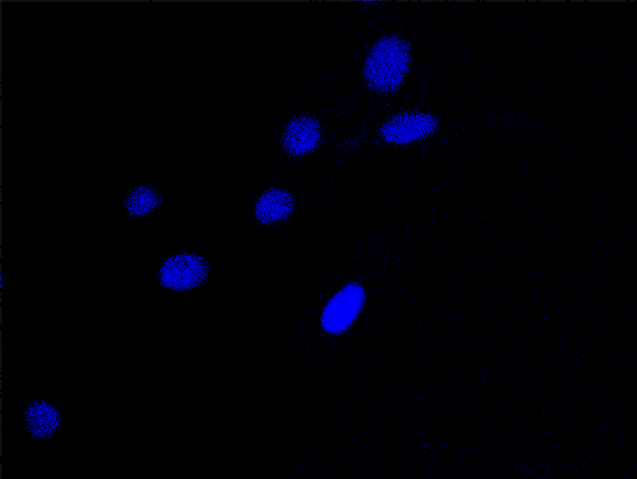

Supplement: Supplementary file 1 [file DataSheet1.zip › figure3/Immunofluorescence/Fig3-E MMP-13/IL-1β -DAPI.png]

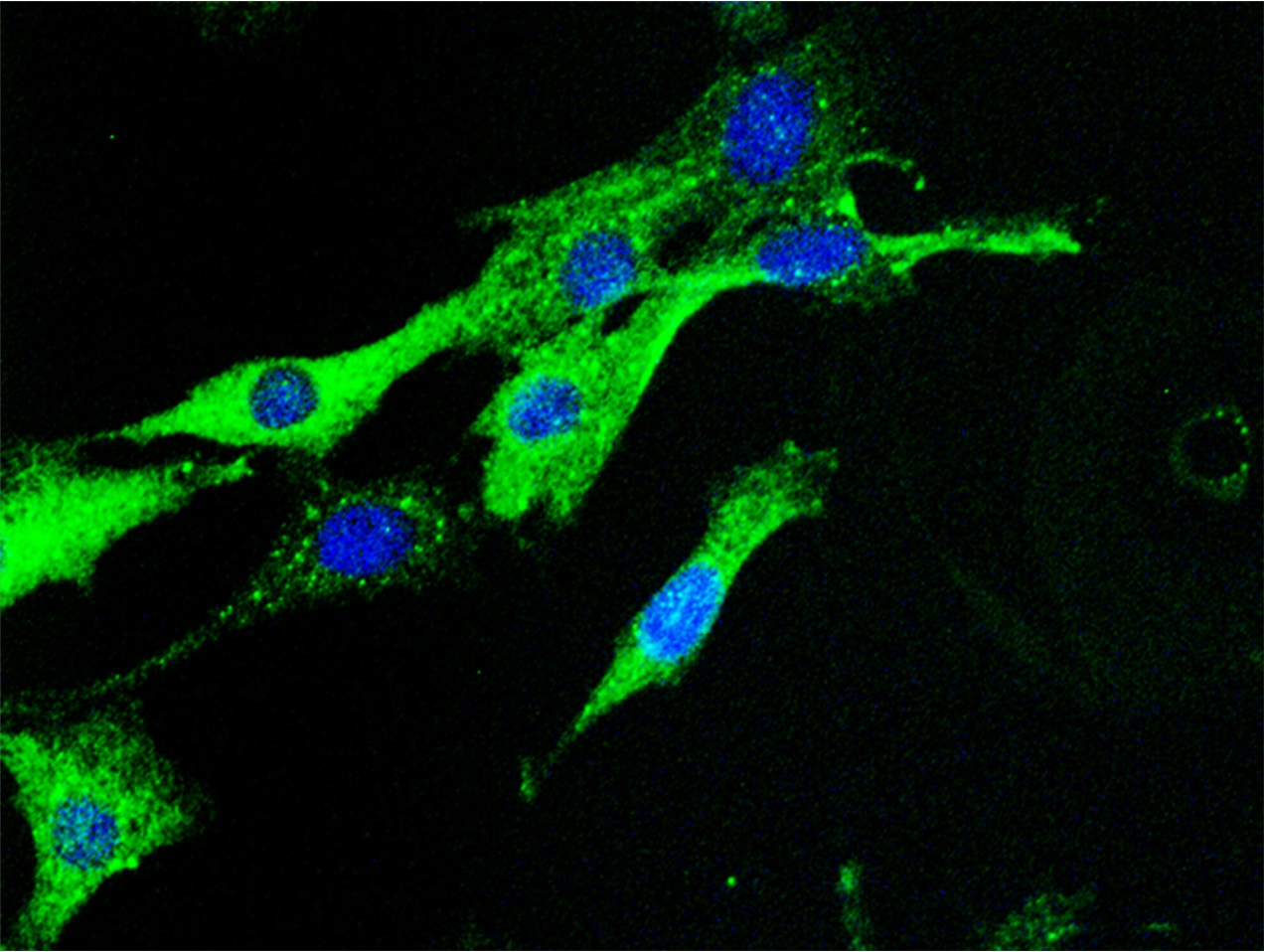

Supplement: Supplementary file 1 [file DataSheet1.zip › figure3/Immunofluorescence/Fig3-E MMP-13/IL-1β -MMP-13.jpg]

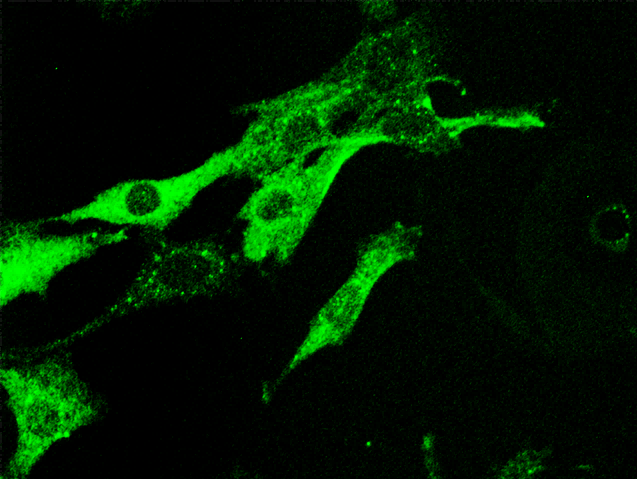

Supplement: Supplementary file 1 [file DataSheet1.zip › figure3/Immunofluorescence/Fig3-E MMP-13/IL-1β-Merge.png]

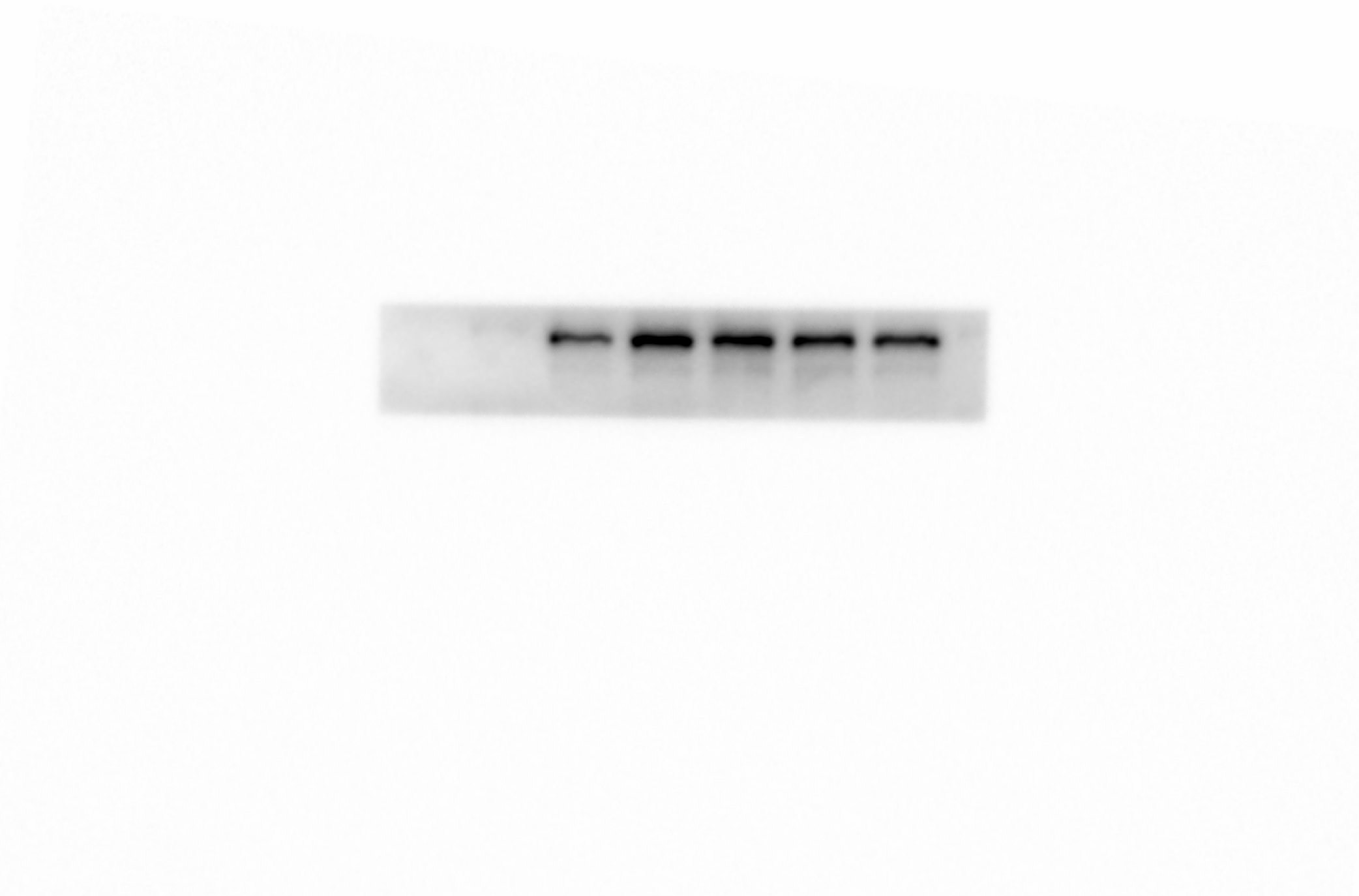

Supplement: Supplementary file 1 [file DataSheet1.zip › figure3/western bolt/ADAMTS.png]

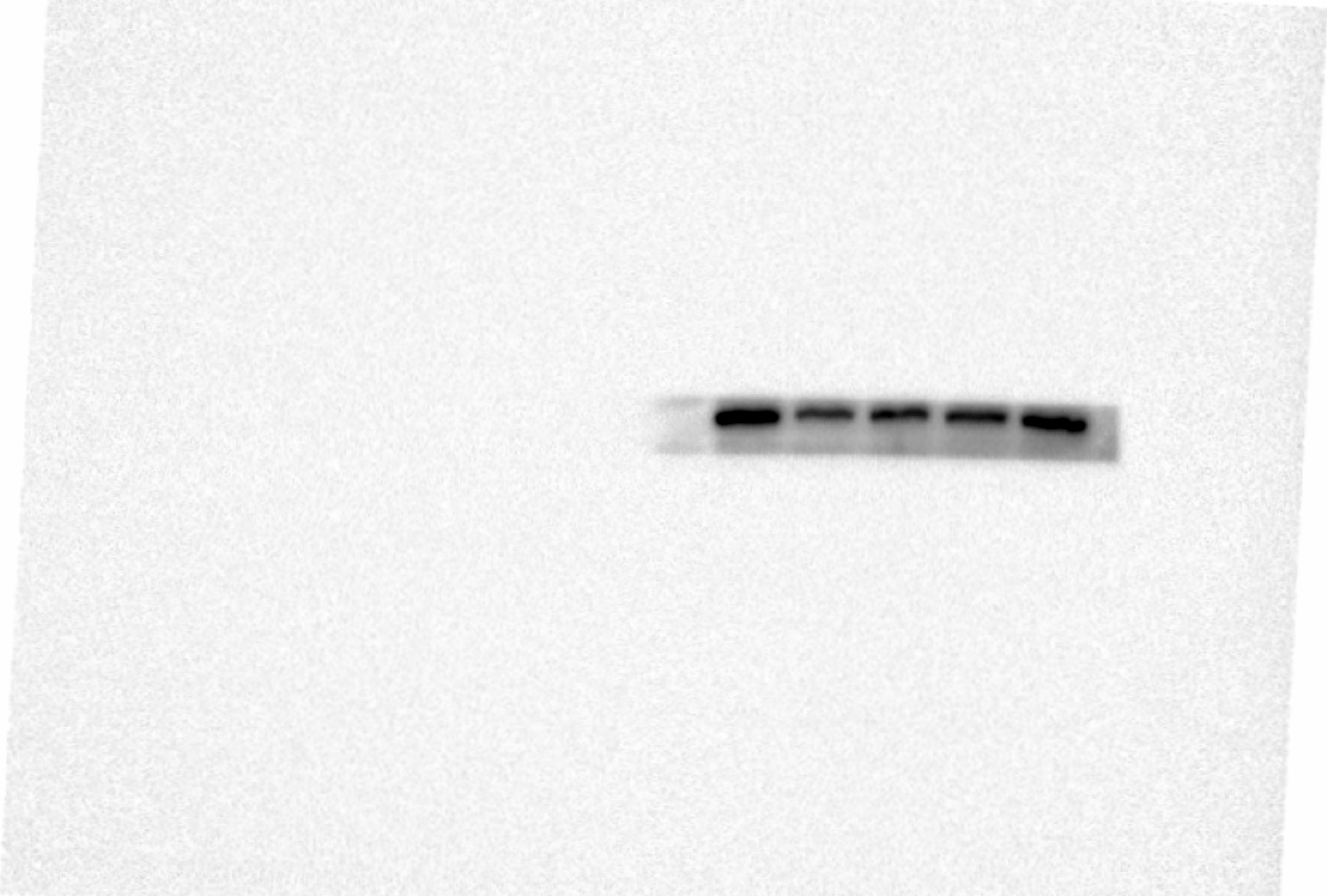

Supplement: Supplementary file 1 [file DataSheet1.zip › figure3/western bolt/Aggrecan.png]

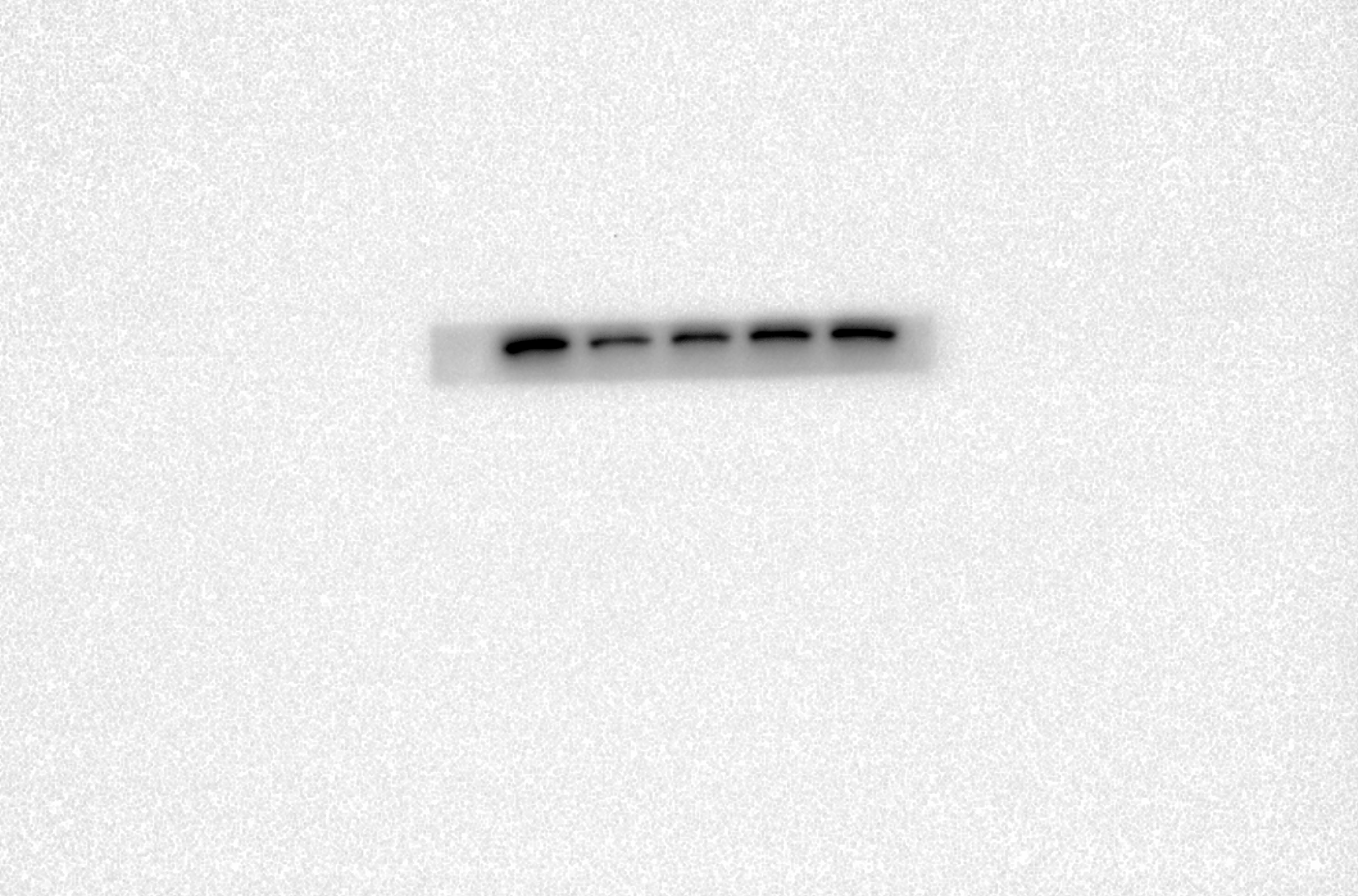

Supplement: Supplementary file 1 [file DataSheet1.zip › figure3/western bolt/Collagen.png]

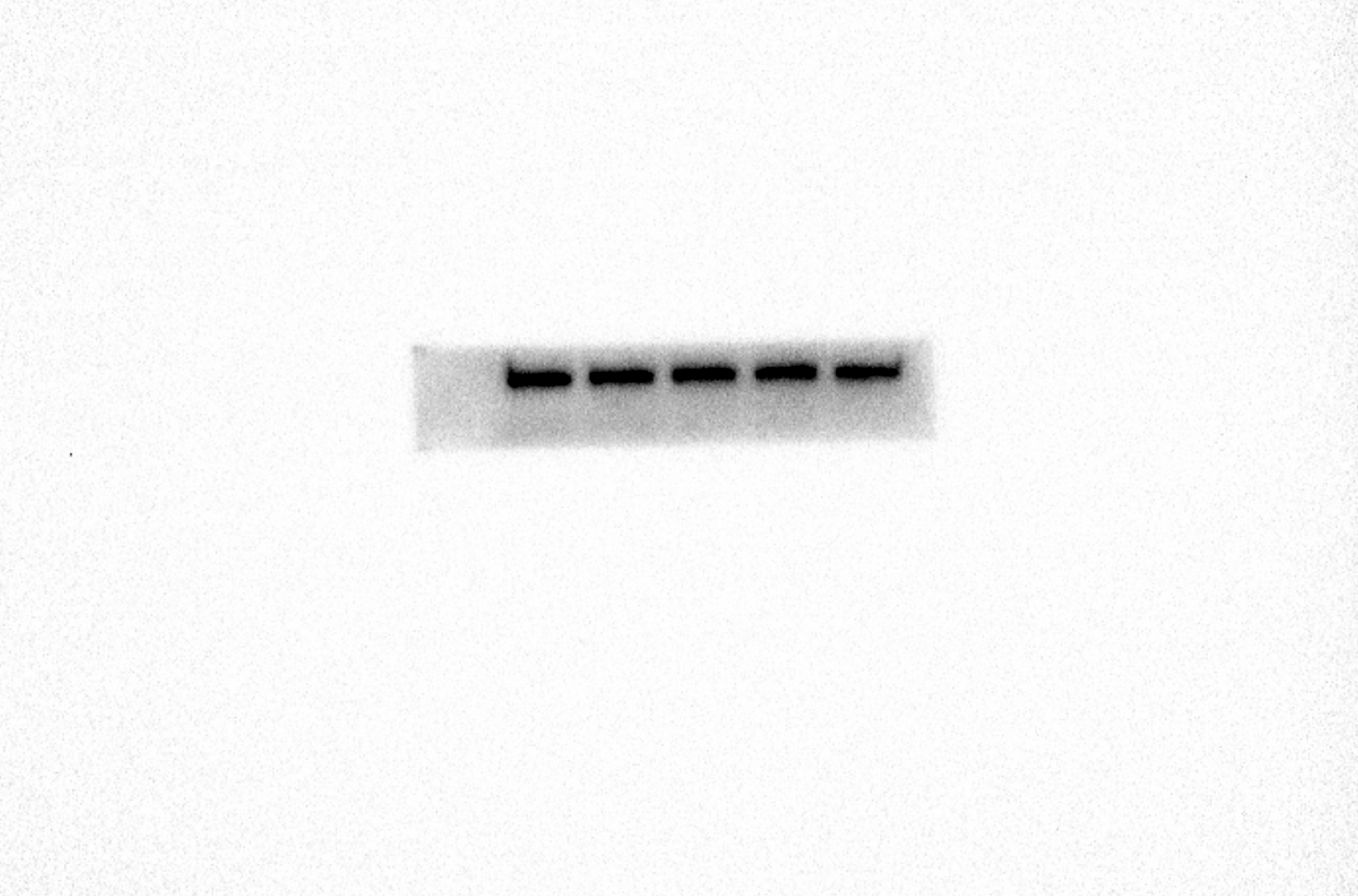

Supplement: Supplementary file 1 [file DataSheet1.zip › figure3/western bolt/GAPDH(1).png]

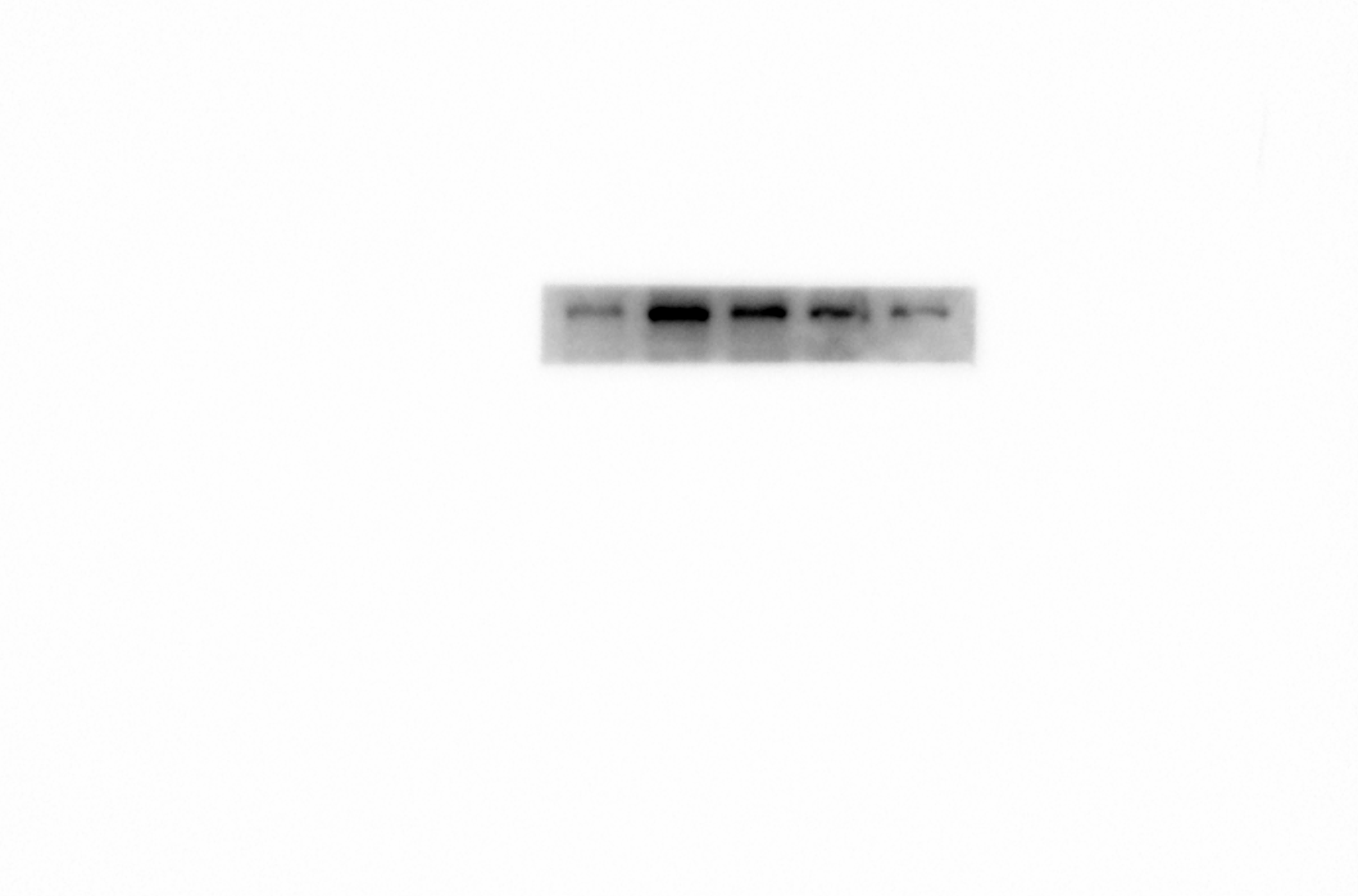

Supplement: Supplementary file 1 [file DataSheet1.zip › figure3/western bolt/MMP13.png]

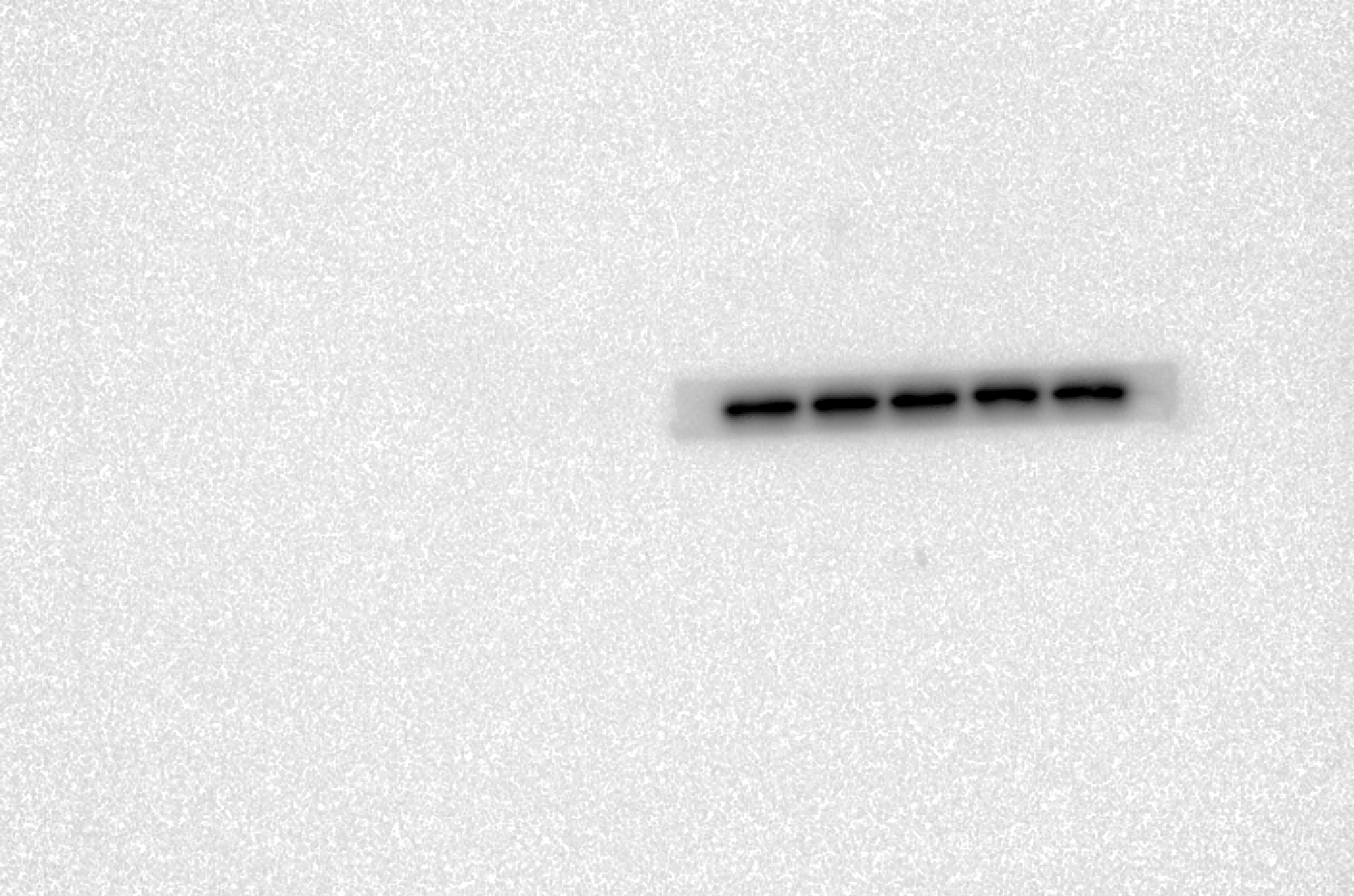

Supplement: Supplementary file 1 [file DataSheet1.zip › figure4/Fig4-A western bolt/GAPDH.png]

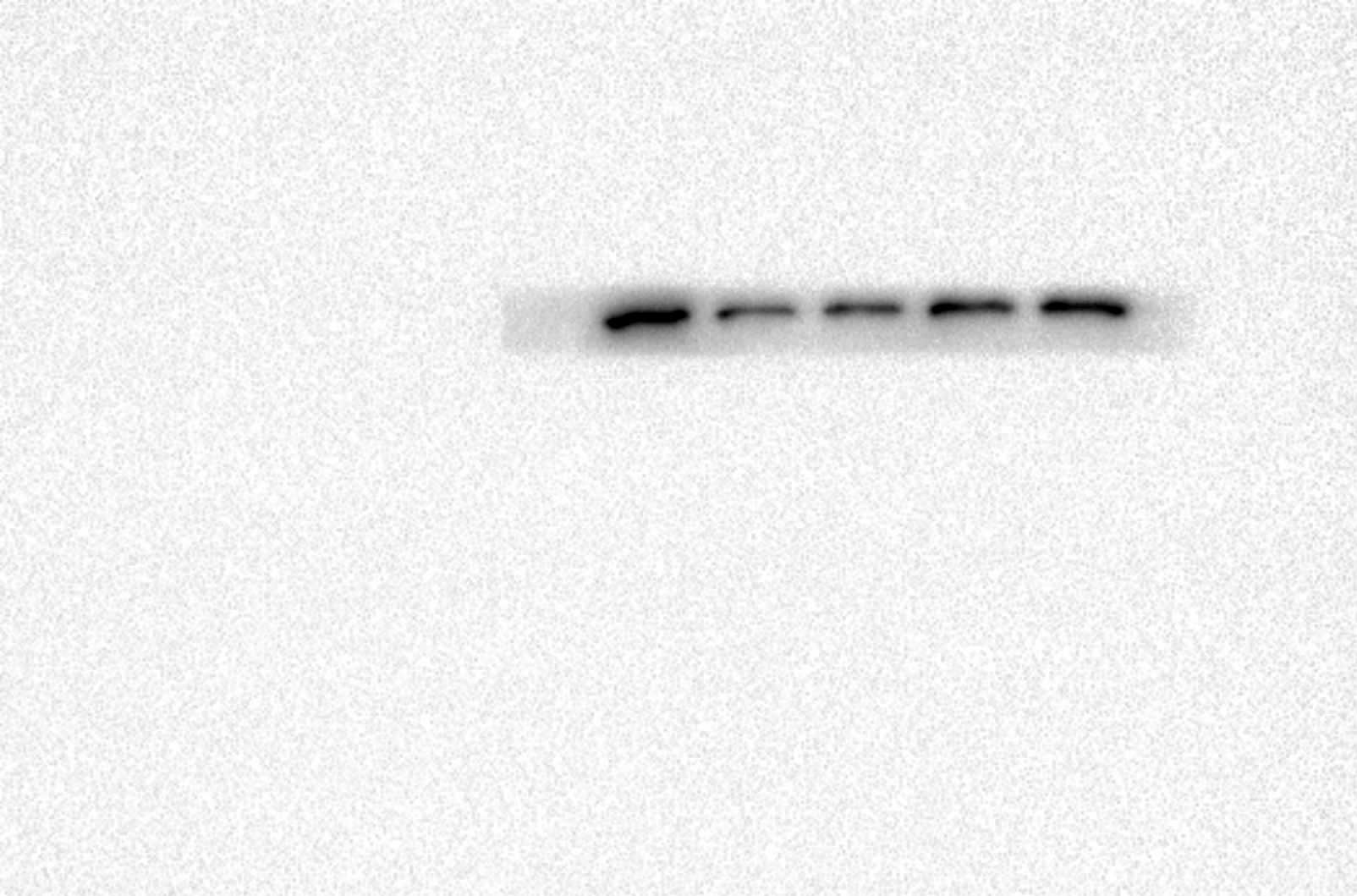

Supplement: Supplementary file 1 [file DataSheet1.zip › figure4/Fig4-A western bolt/IKBa.png]

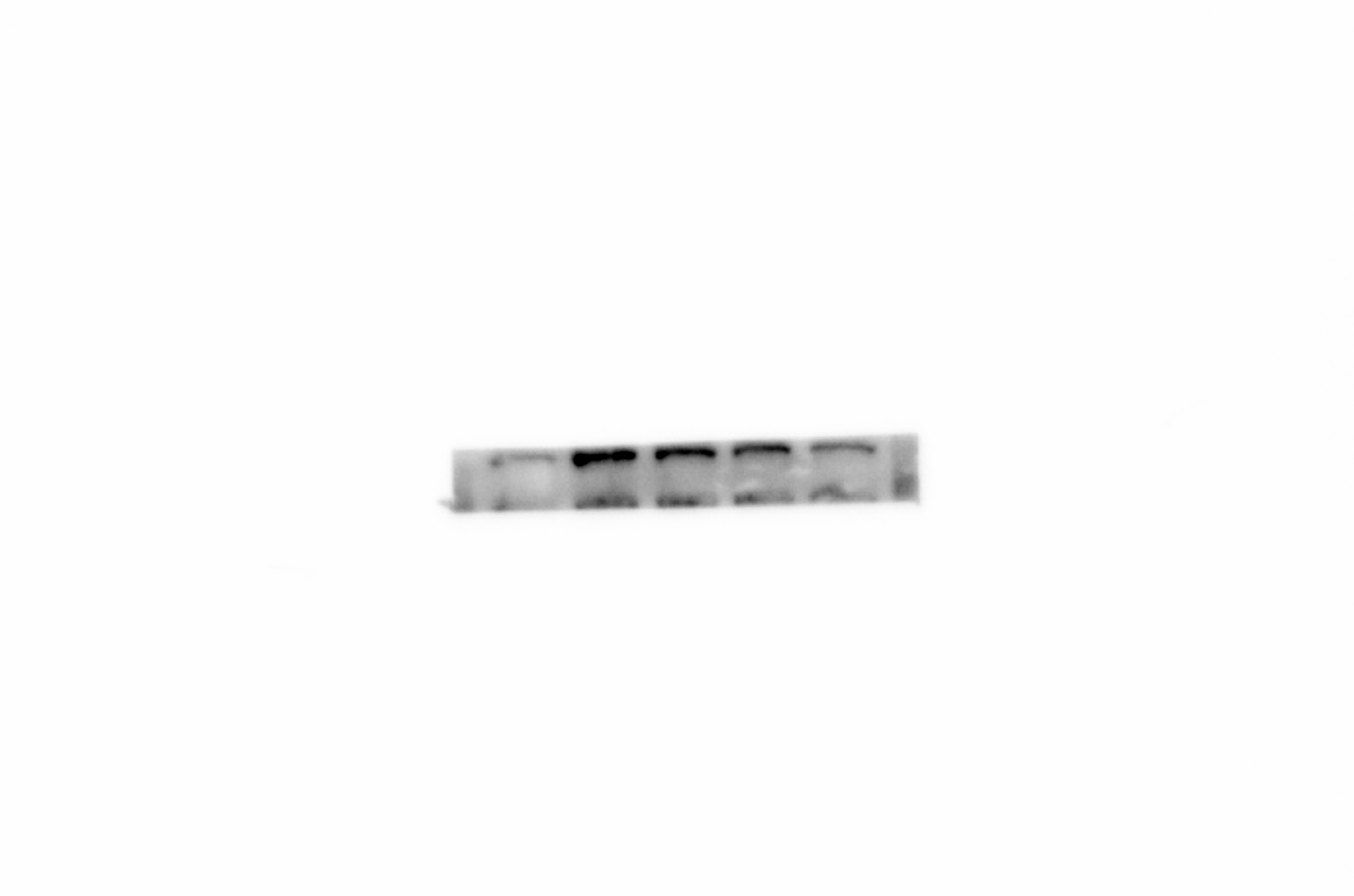

Supplement: Supplementary file 1 [file DataSheet1.zip › figure4/Fig4-A western bolt/P-IkBa.png]

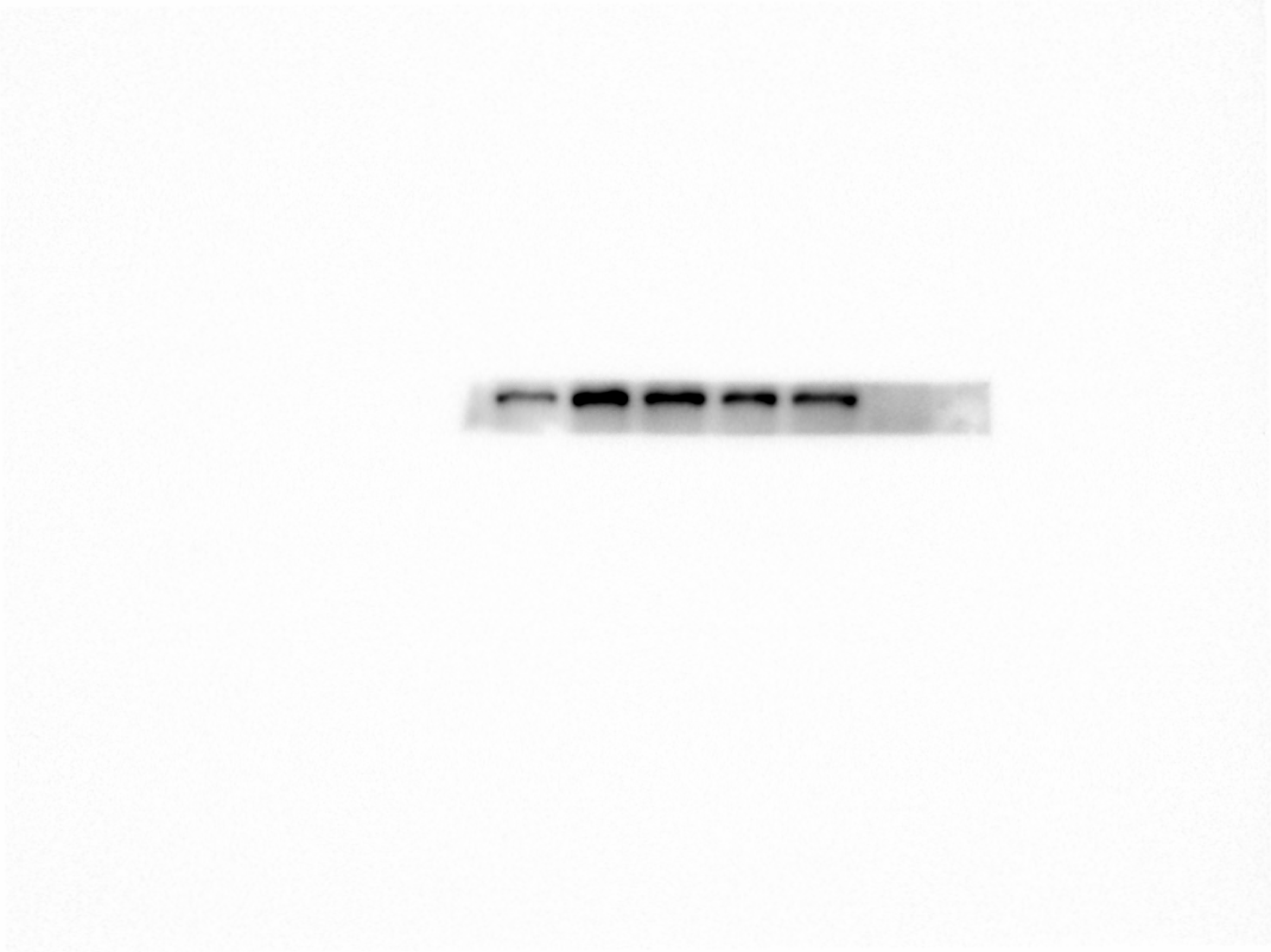

Supplement: Supplementary file 1 [file DataSheet1.zip › figure4/Fig4-A western bolt/P-P65.png]

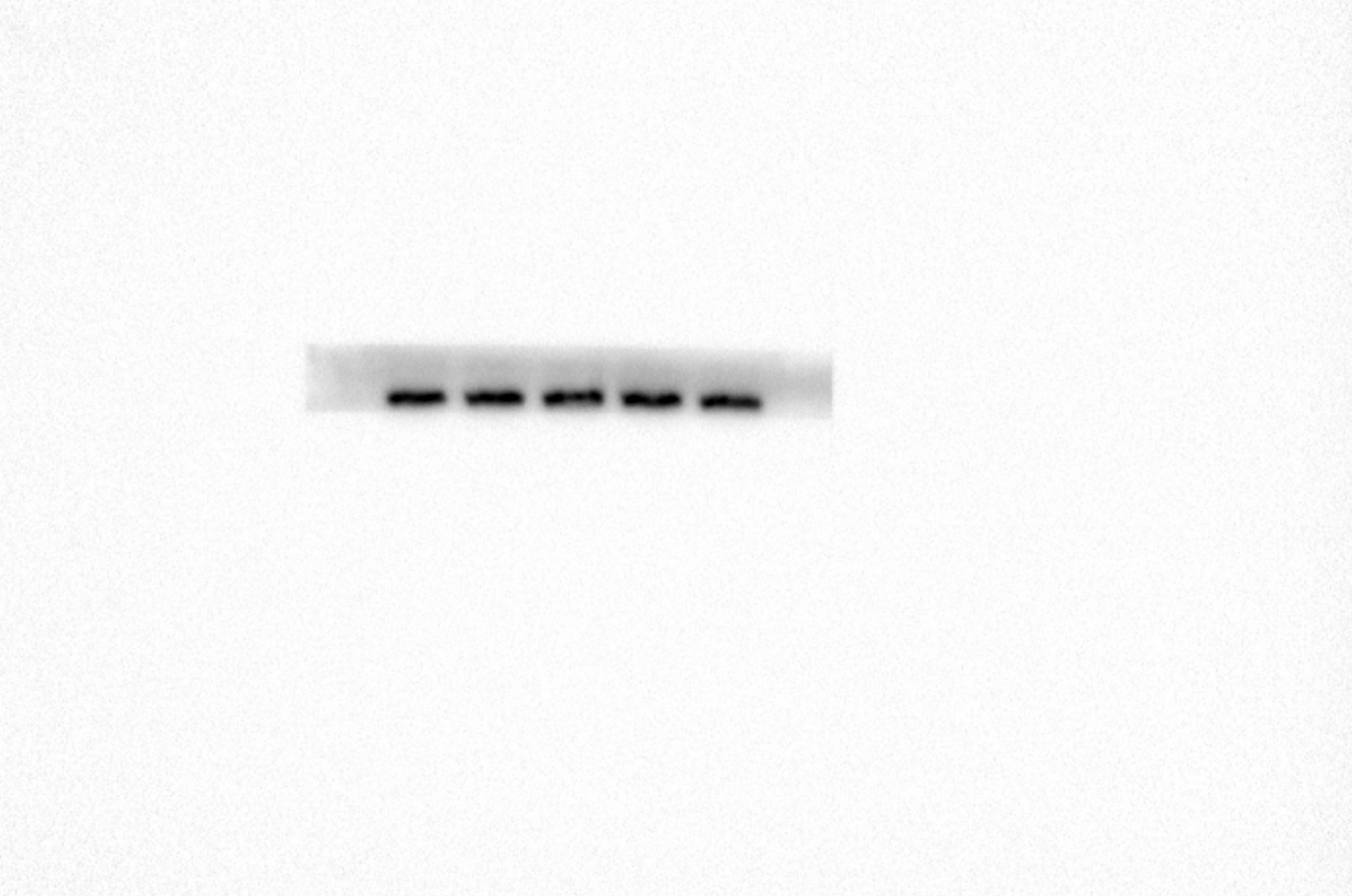

Supplement: Supplementary file 1 [file DataSheet1.zip › figure4/Fig4-A western bolt/P65.png]

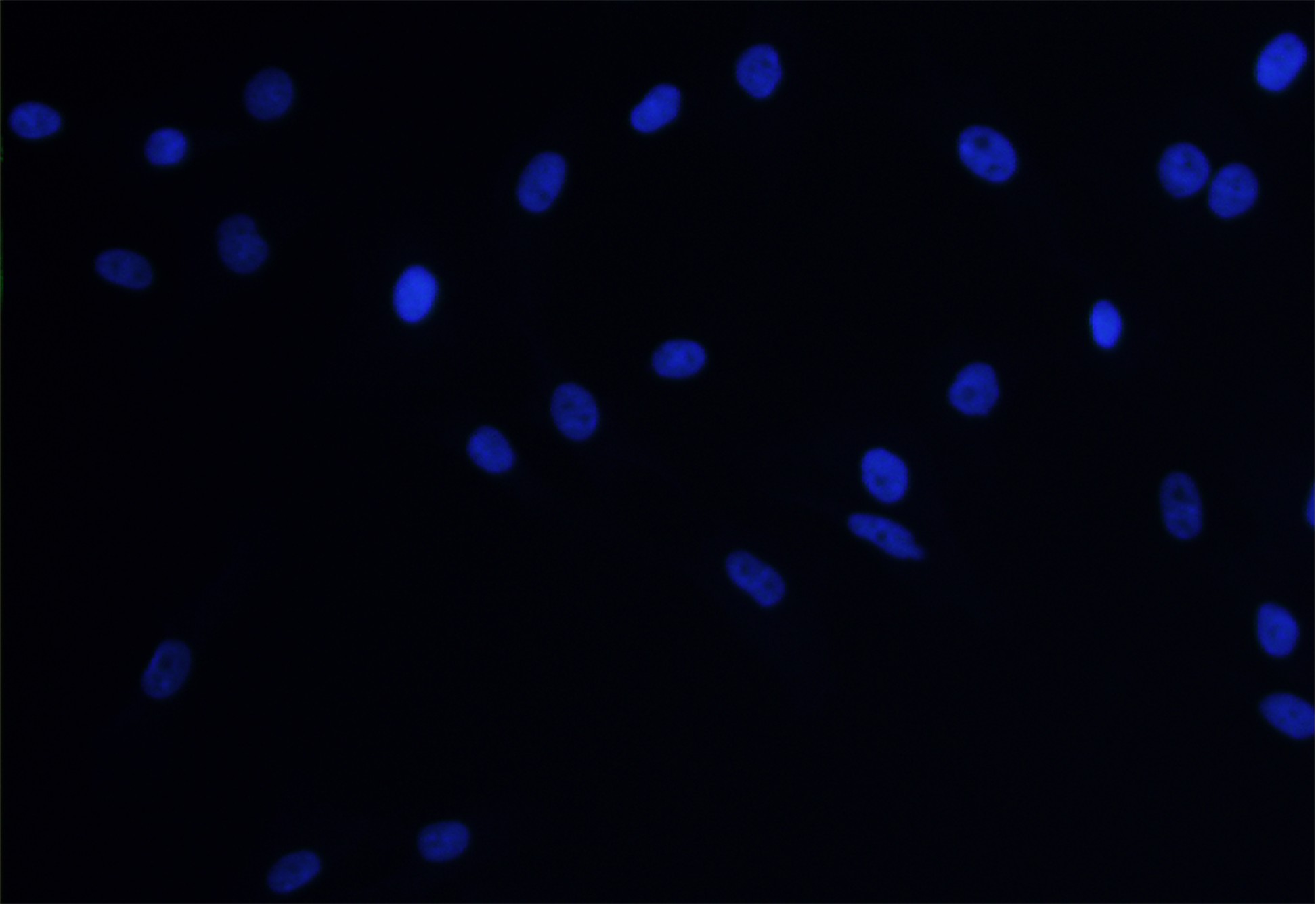

Supplement: Supplementary file 1 [file DataSheet1.zip › figure4/Fig4-D Immunofluorescence/Betulin+IL-1β -DAPI.jpg]

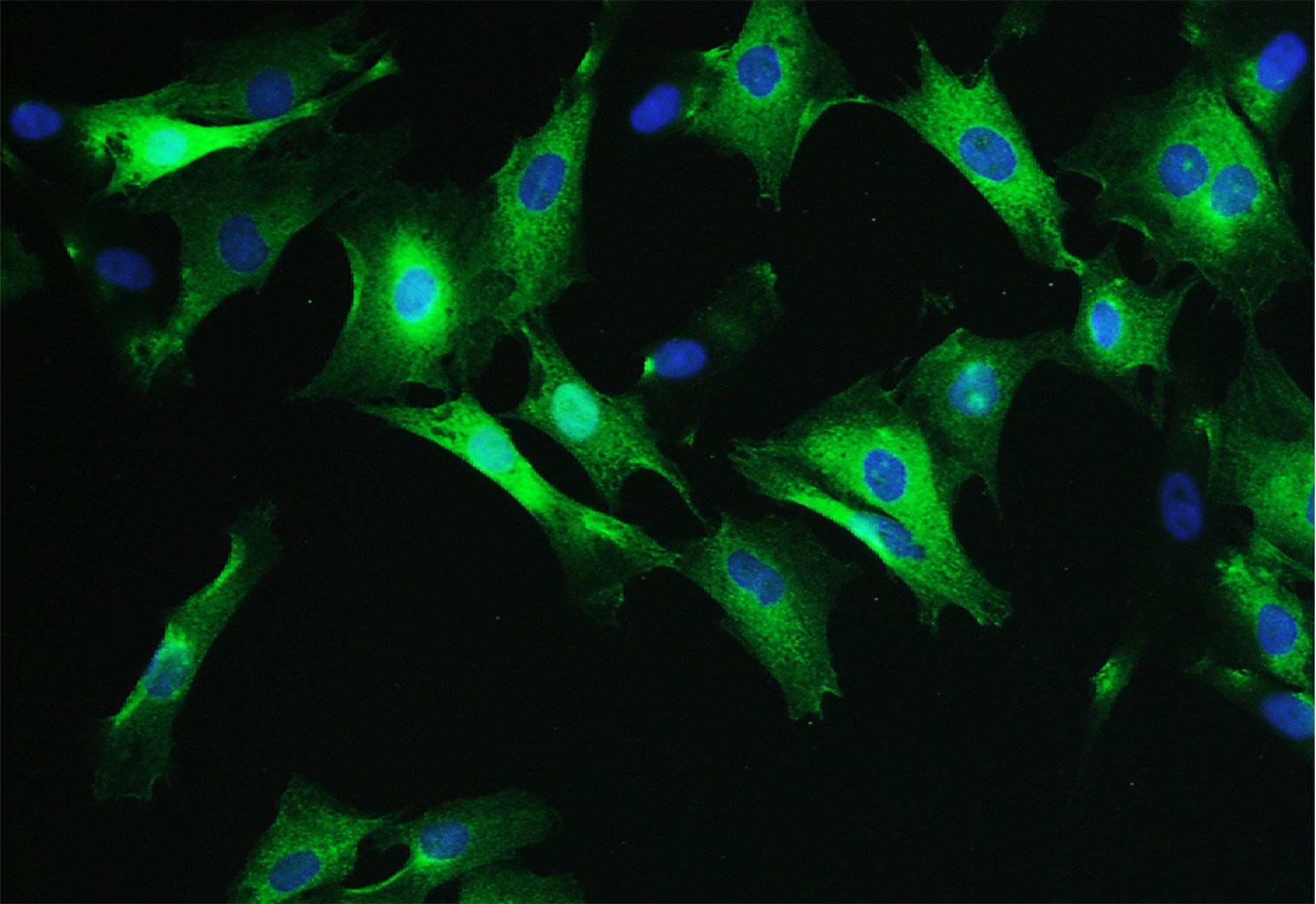

Supplement: Supplementary file 1 [file DataSheet1.zip › figure4/Fig4-D Immunofluorescence/Betulin+IL-1β-Merge.jpg]

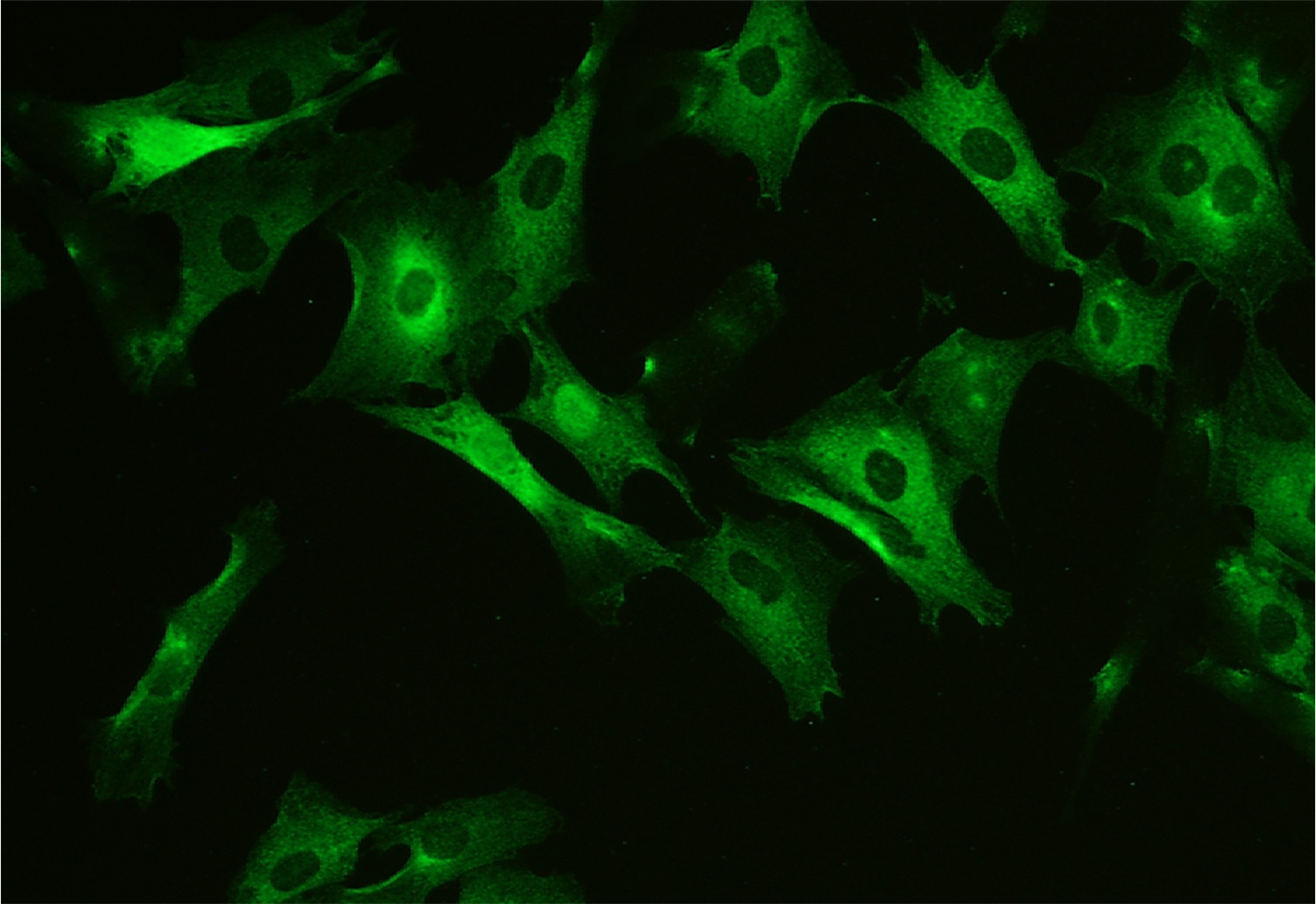

Supplement: Supplementary file 1 [file DataSheet1.zip › figure4/Fig4-D Immunofluorescence/Betulin+IL-1β-P65.jpg]

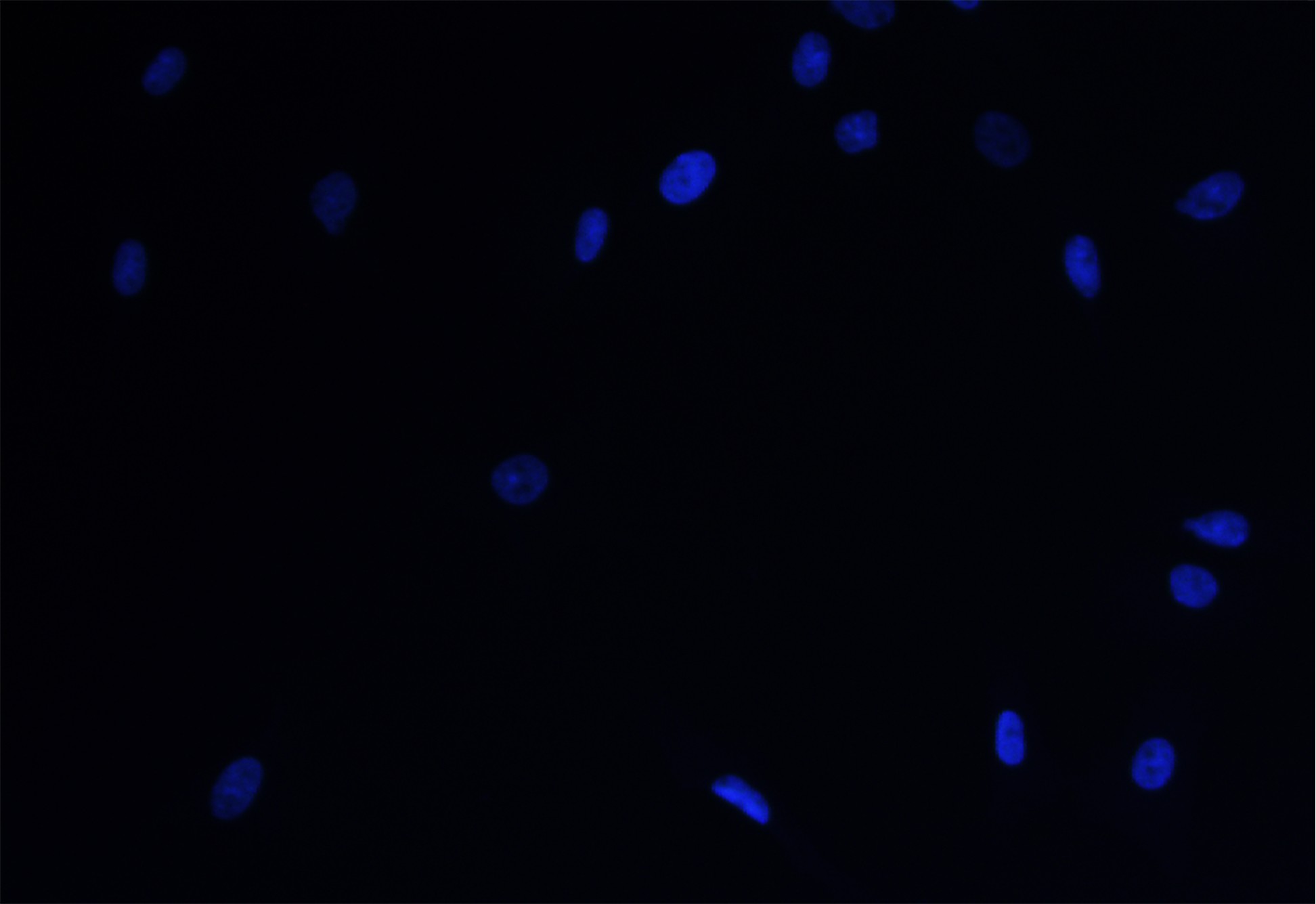

Supplement: Supplementary file 1 [file DataSheet1.zip › figure4/Fig4-D Immunofluorescence/control-DAPI.jpg]

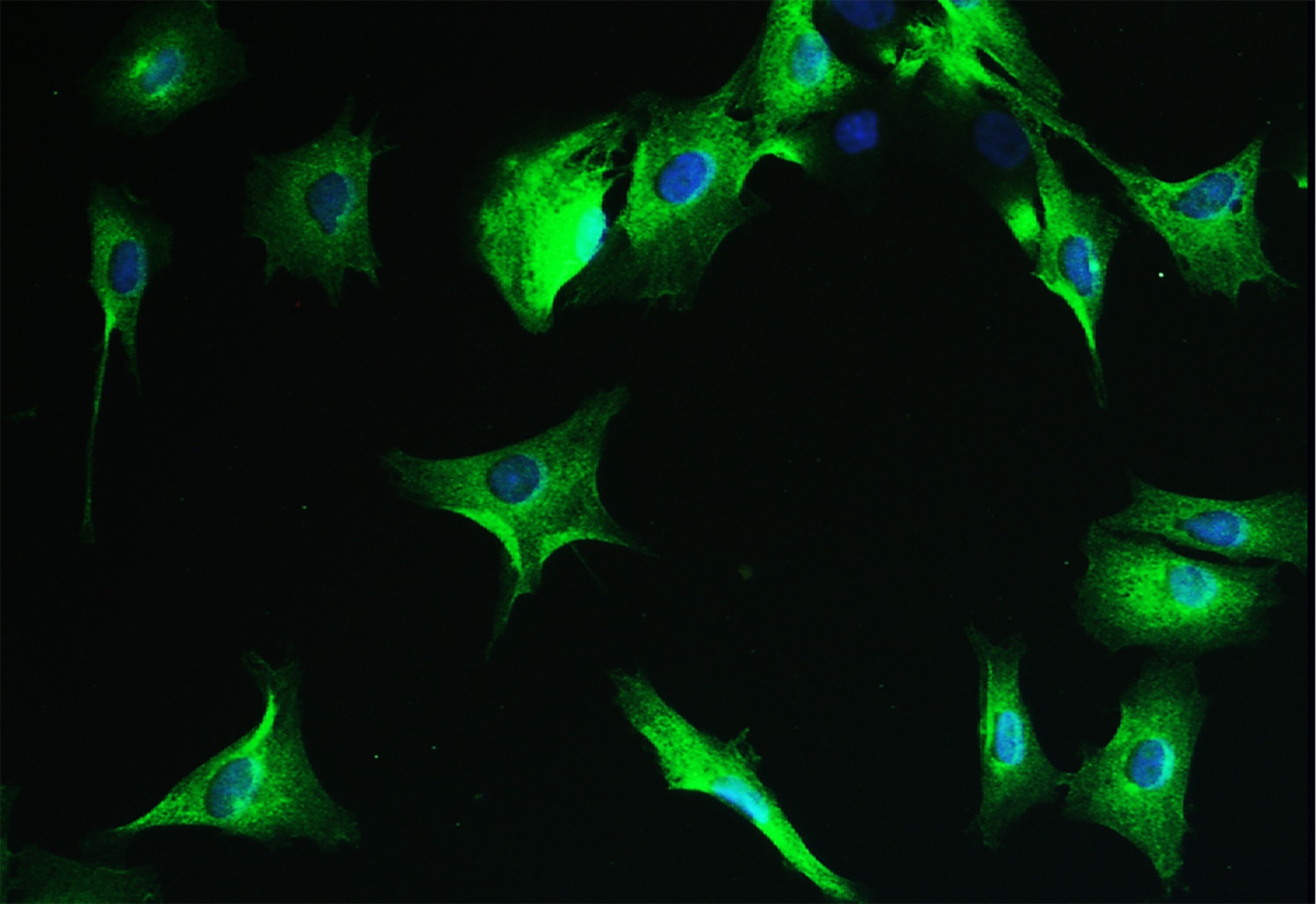

Supplement: Supplementary file 1 [file DataSheet1.zip › figure4/Fig4-D Immunofluorescence/control-Merge.jpg]

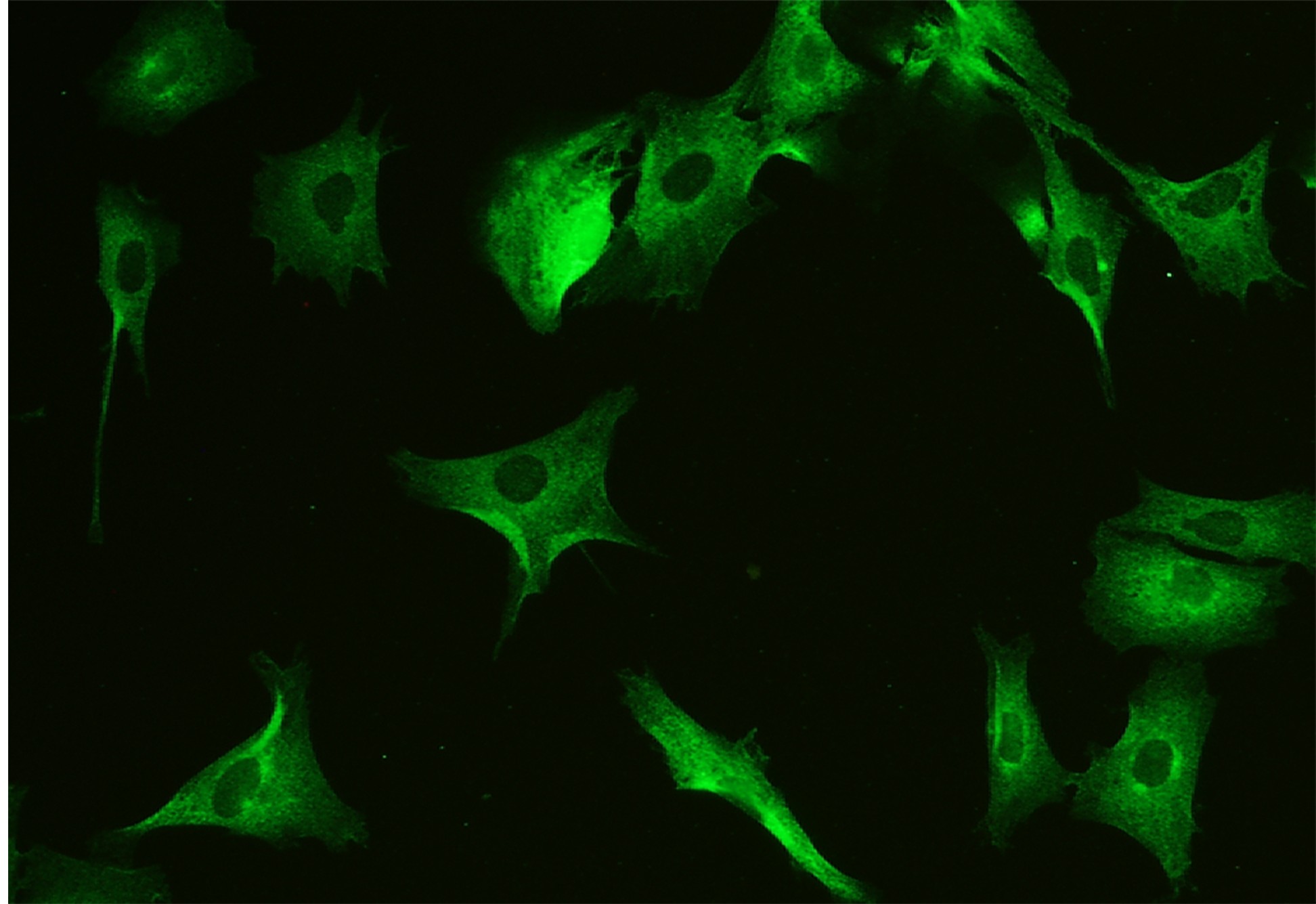

Supplement: Supplementary file 1 [file DataSheet1.zip › figure4/Fig4-D Immunofluorescence/control-P65.jpg]

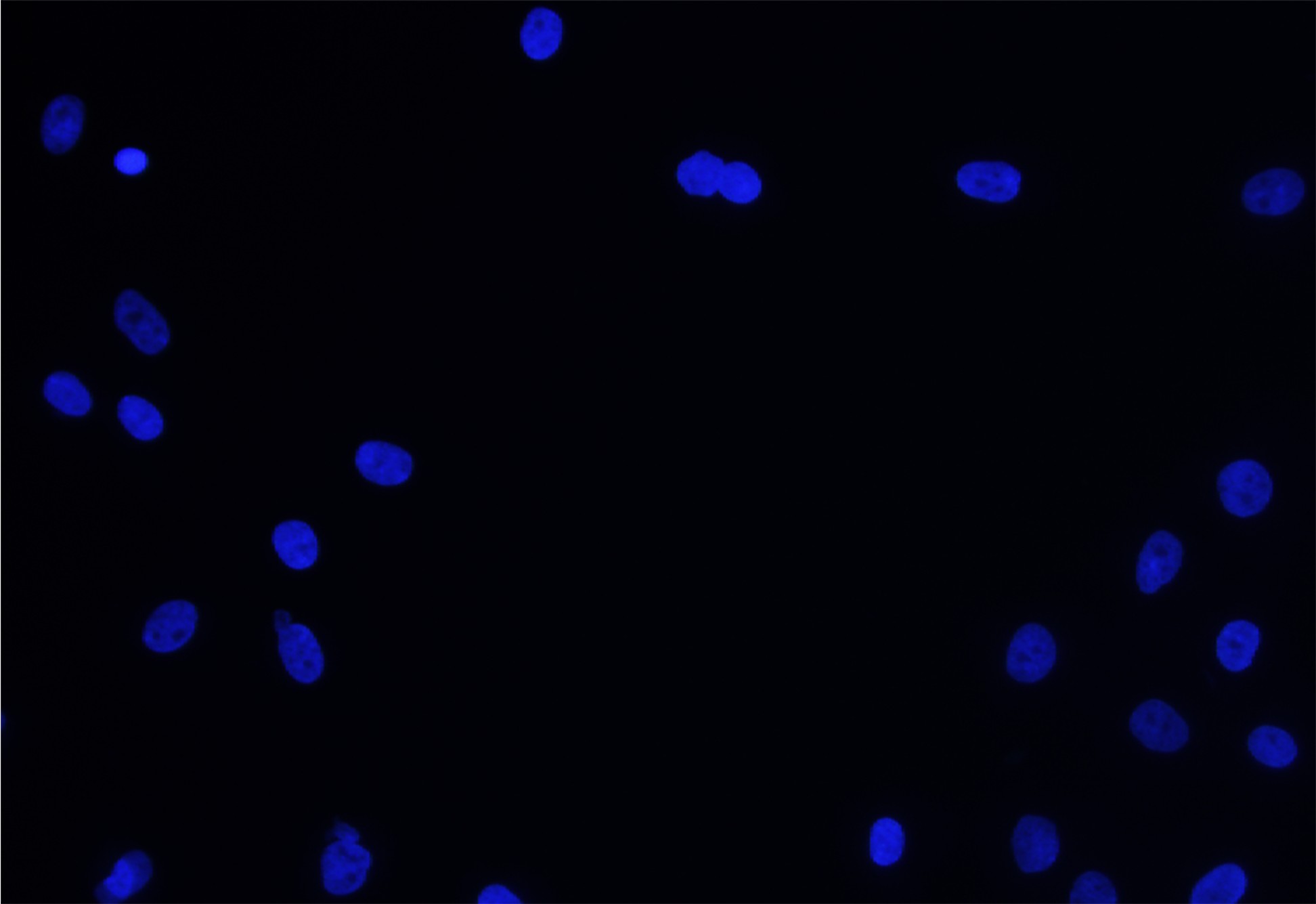

Supplement: Supplementary file 1 [file DataSheet1.zip › figure4/Fig4-D Immunofluorescence/IL-1β -DAPI.jpg]

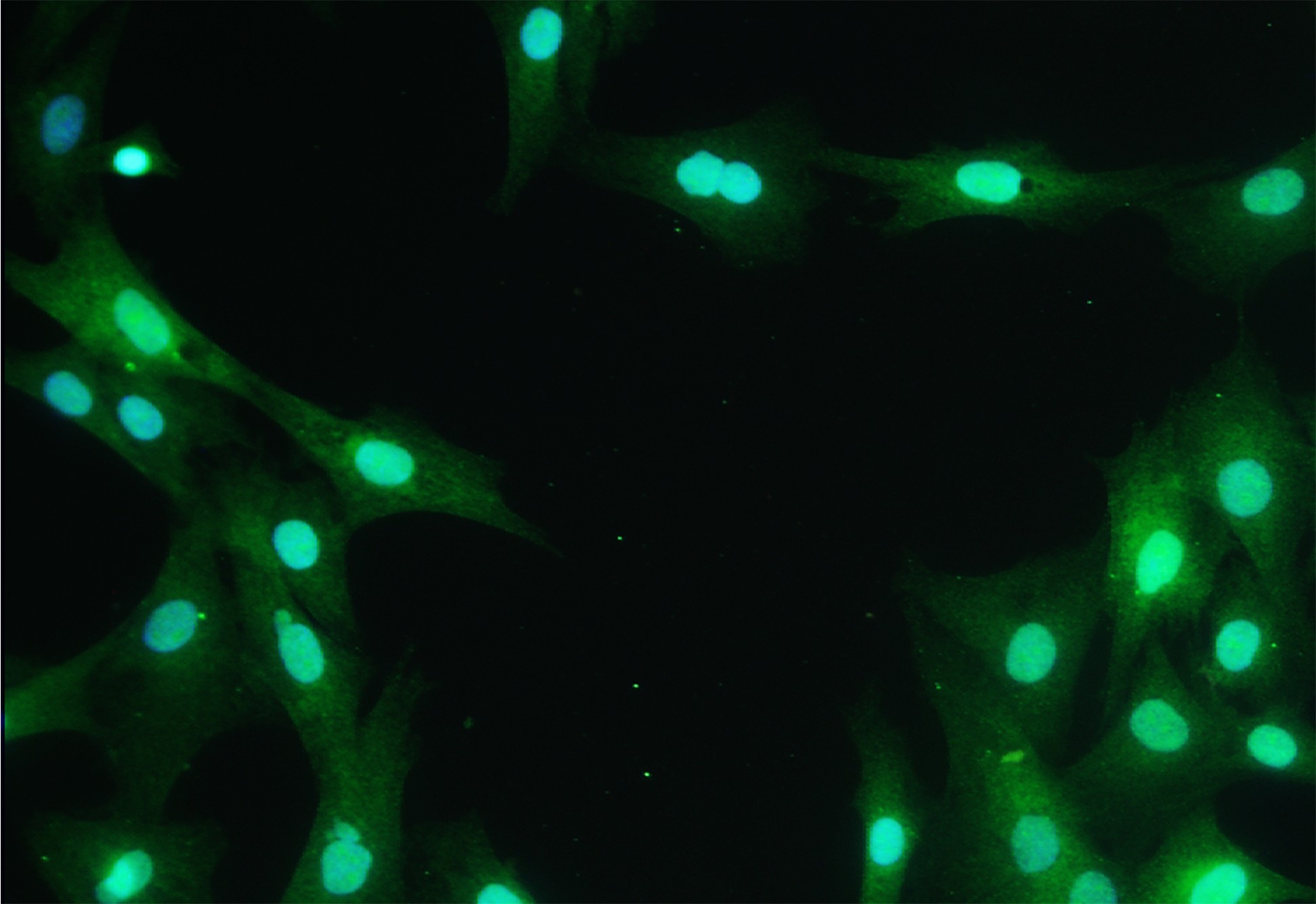

Supplement: Supplementary file 1 [file DataSheet1.zip › figure4/Fig4-D Immunofluorescence/IL-1β -Merge.jpg]

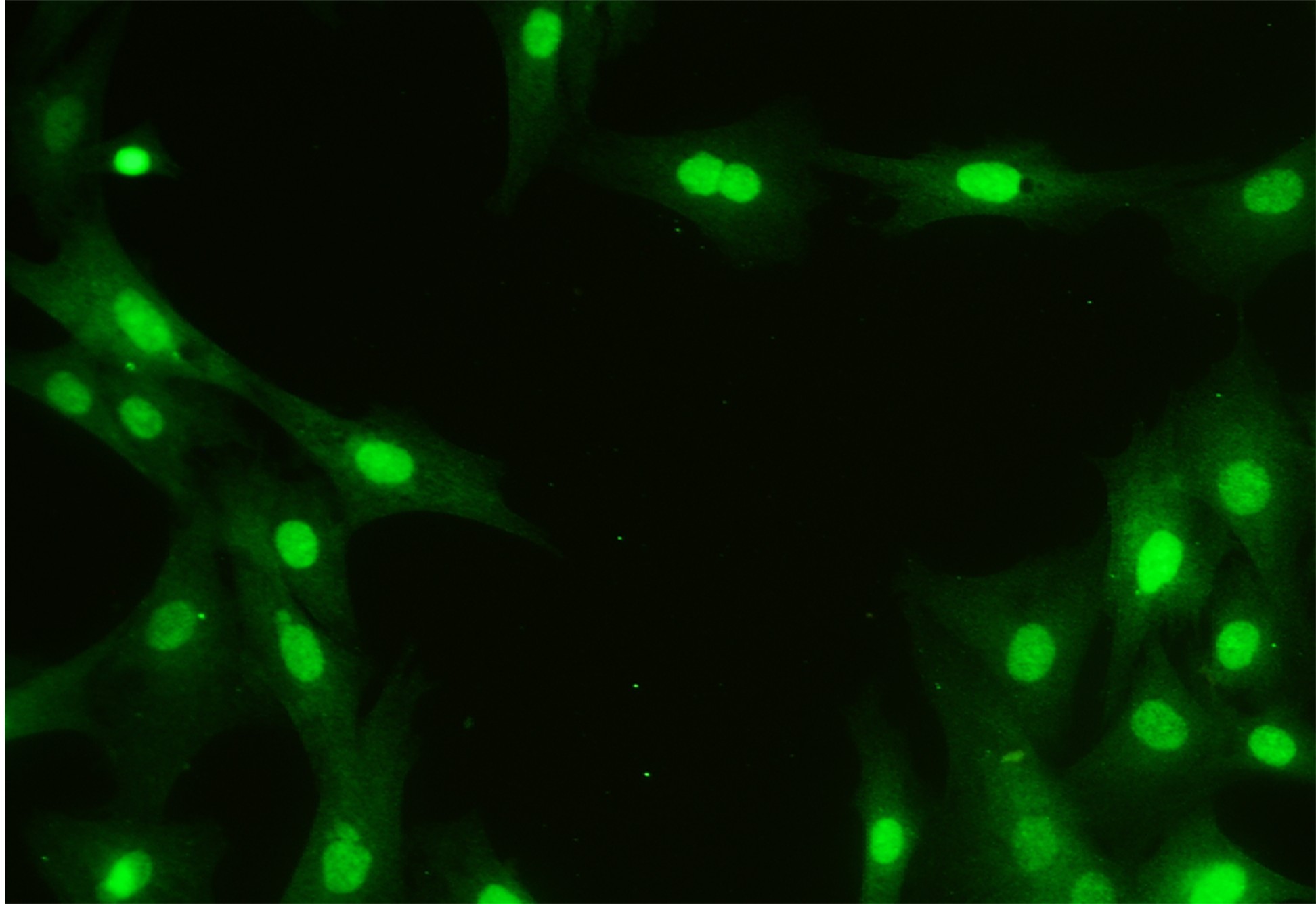

Supplement: Supplementary file 1 [file DataSheet1.zip › figure4/Fig4-D Immunofluorescence/IL-1β -P65.jpg]

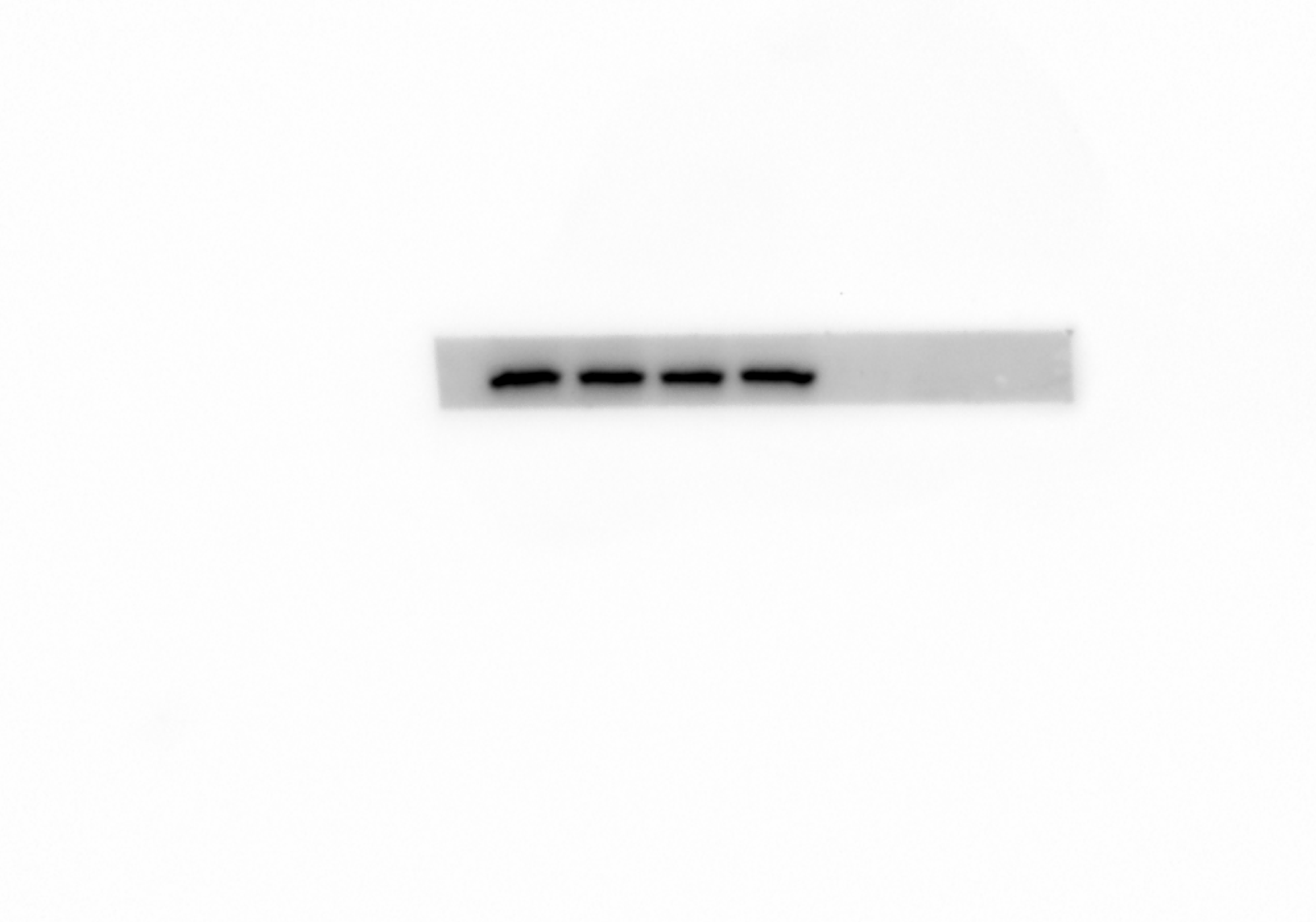

Supplement: Supplementary file 1 [file DataSheet1.zip › figure5/Fig5.A-western bolt/AKT.png]

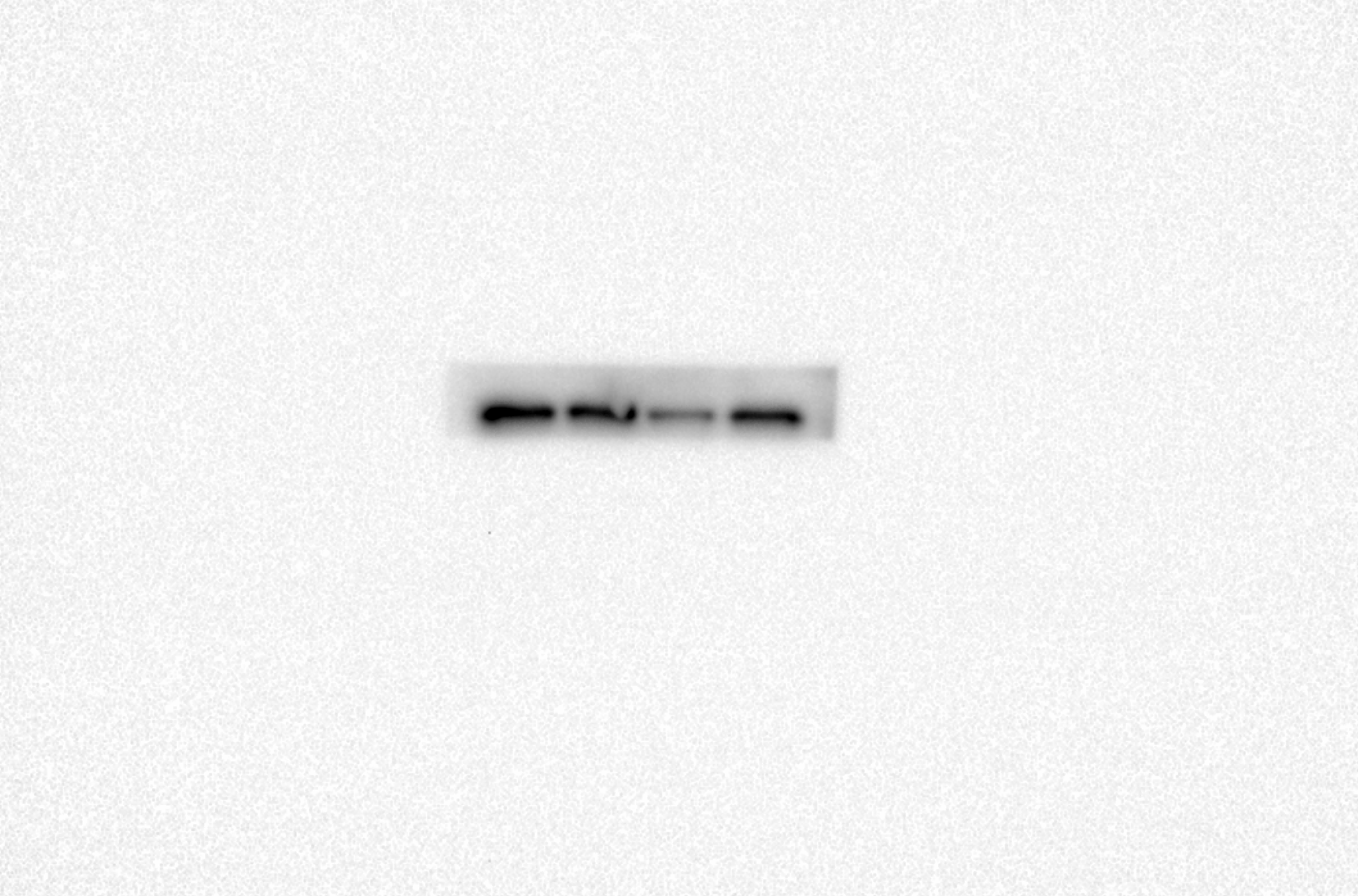

Supplement: Supplementary file 1 [file DataSheet1.zip › figure5/Fig5.A-western bolt/Cy-Nrf.png]

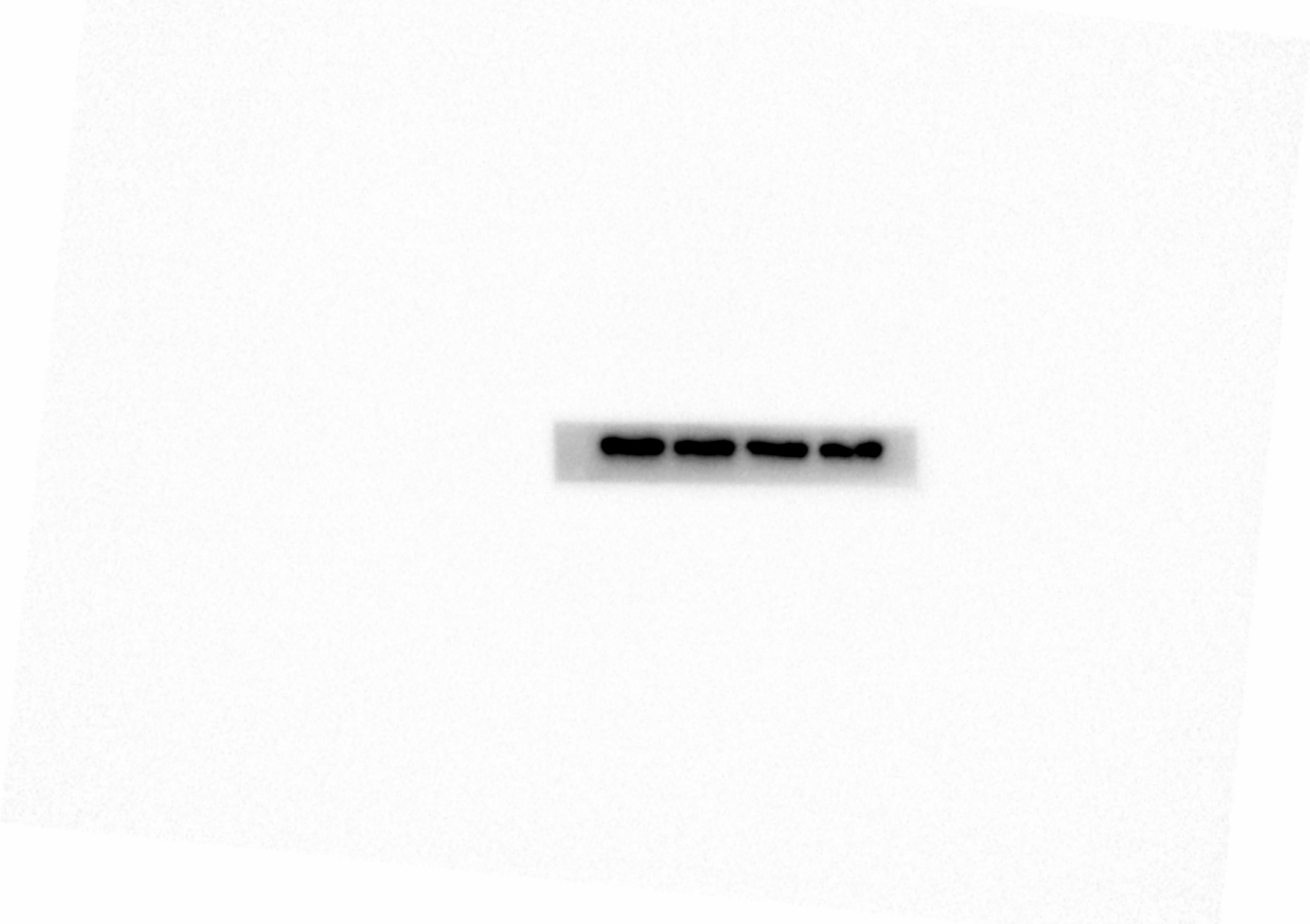

Supplement: Supplementary file 1 [file DataSheet1.zip › figure5/Fig5.A-western bolt/GAPDH.png]

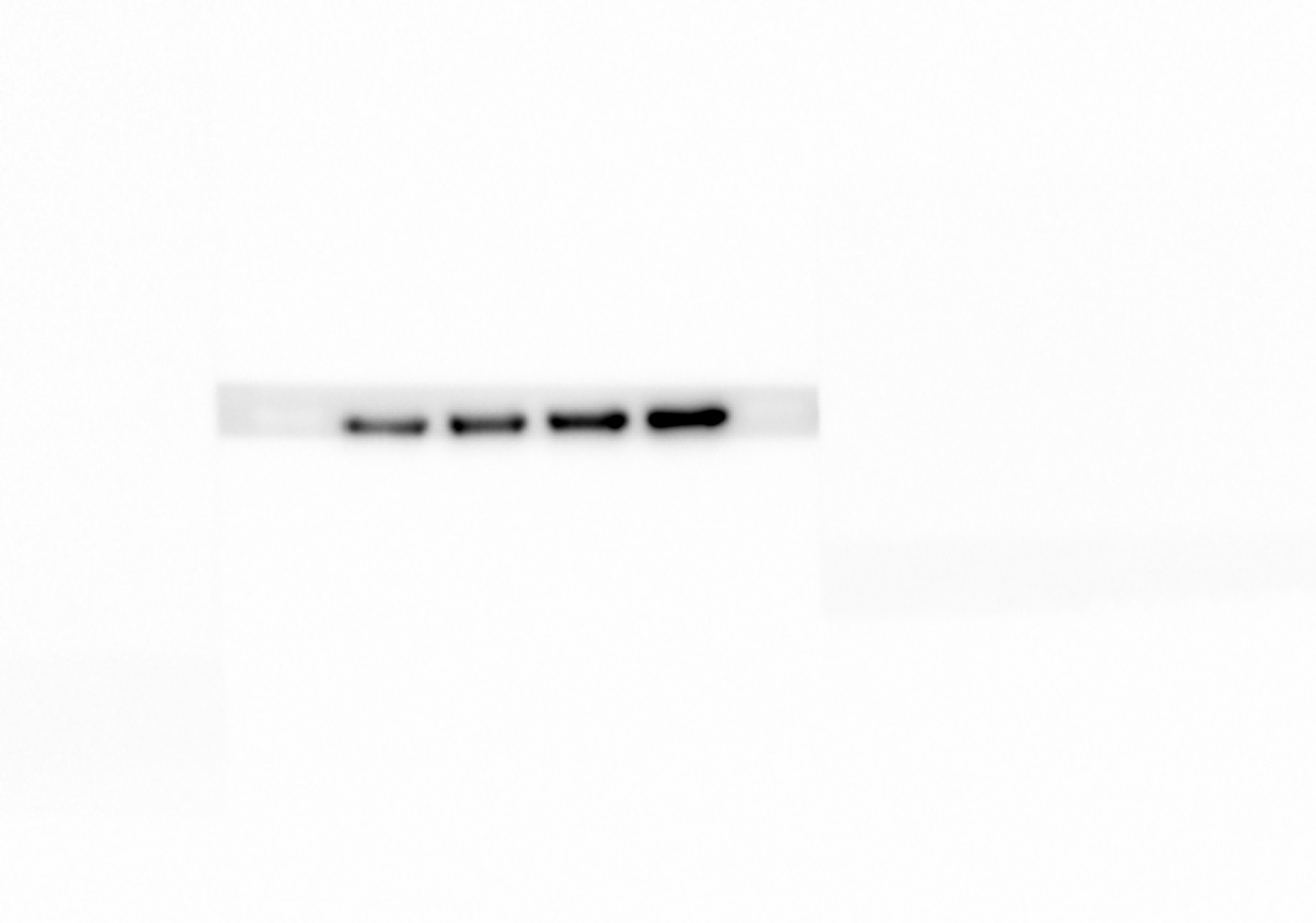

Supplement: Supplementary file 1 [file DataSheet1.zip › figure5/Fig5.A-western bolt/HO-1.png]

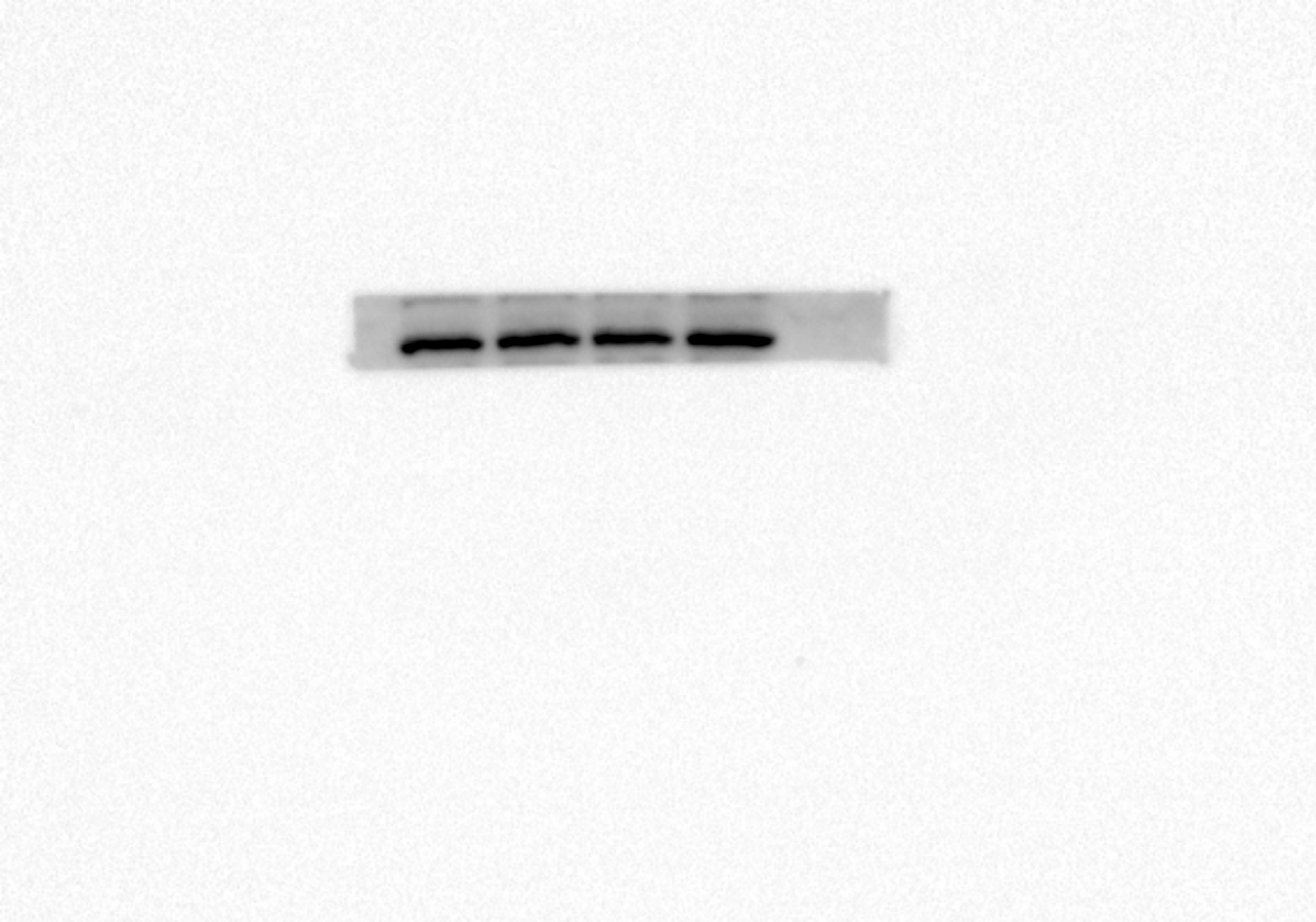

Supplement: Supplementary file 1 [file DataSheet1.zip › figure5/Fig5.A-western bolt/Lamin B.png]

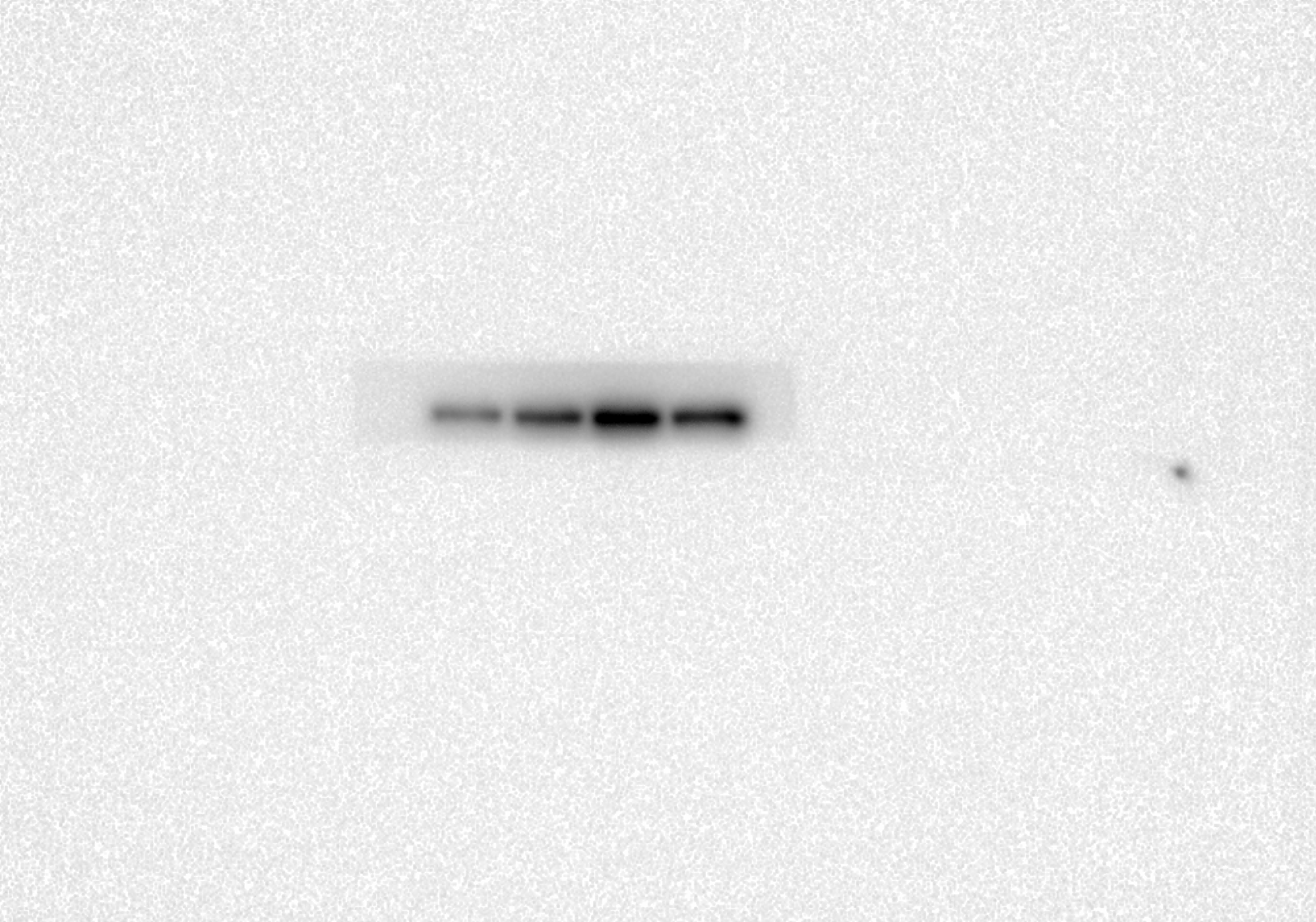

Supplement: Supplementary file 1 [file DataSheet1.zip › figure5/Fig5.A-western bolt/Nu-Nrf.png]

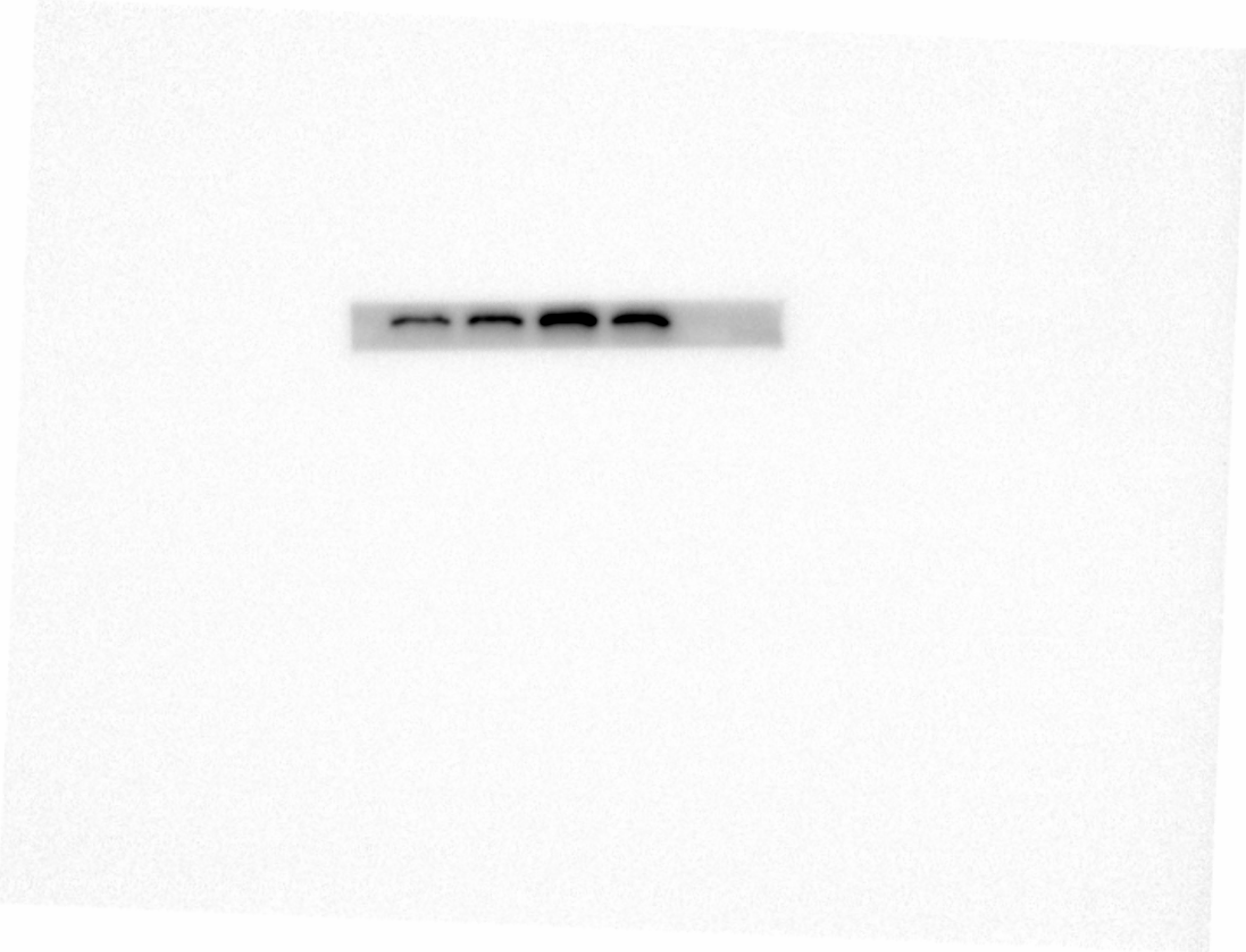

Supplement: Supplementary file 1 [file DataSheet1.zip › figure5/Fig5.A-western bolt/P-AKT.png]

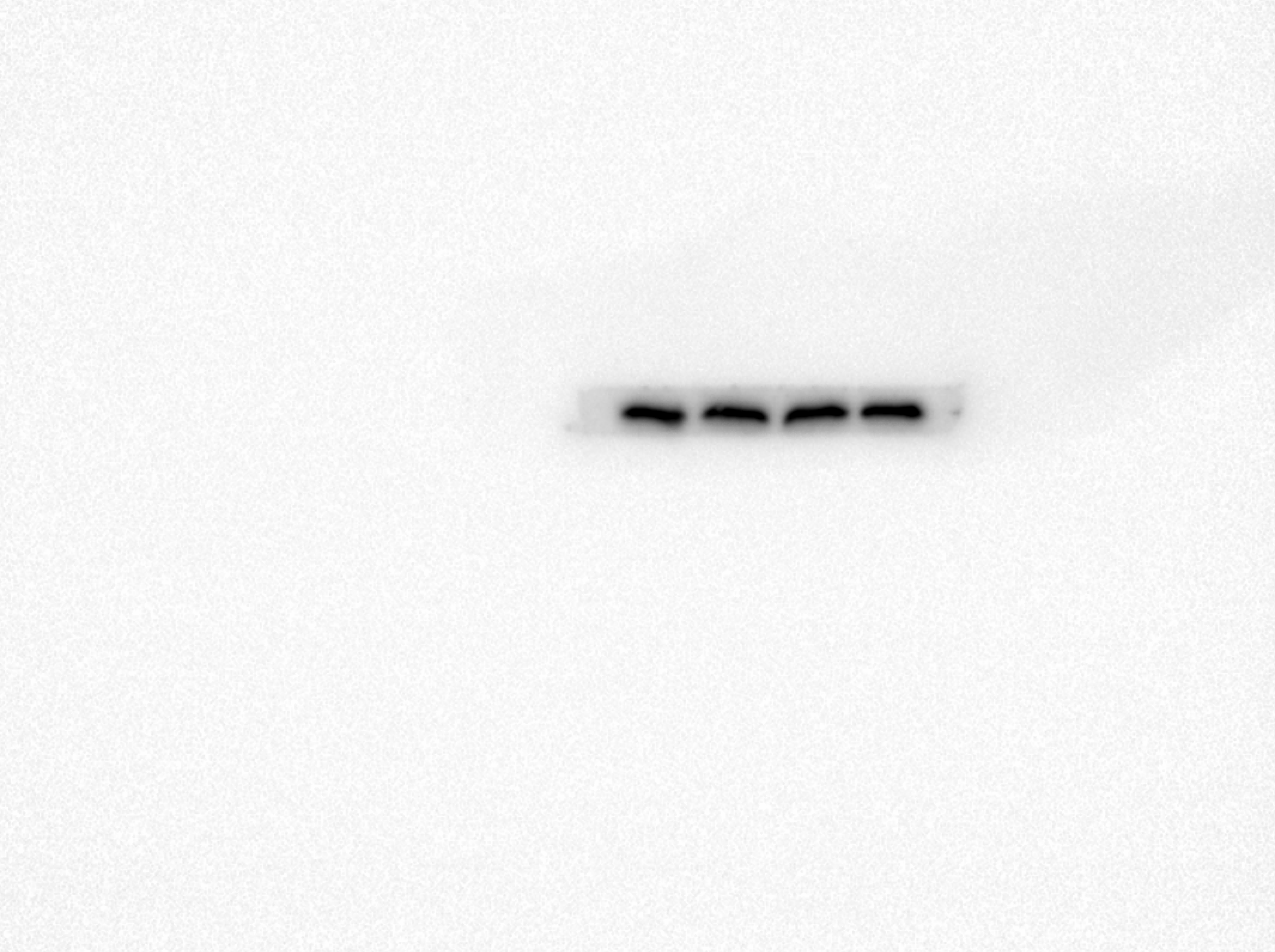

Supplement: Supplementary file 1 [file DataSheet1.zip › figure6/Fig6.A-western bolt/AKT.png]

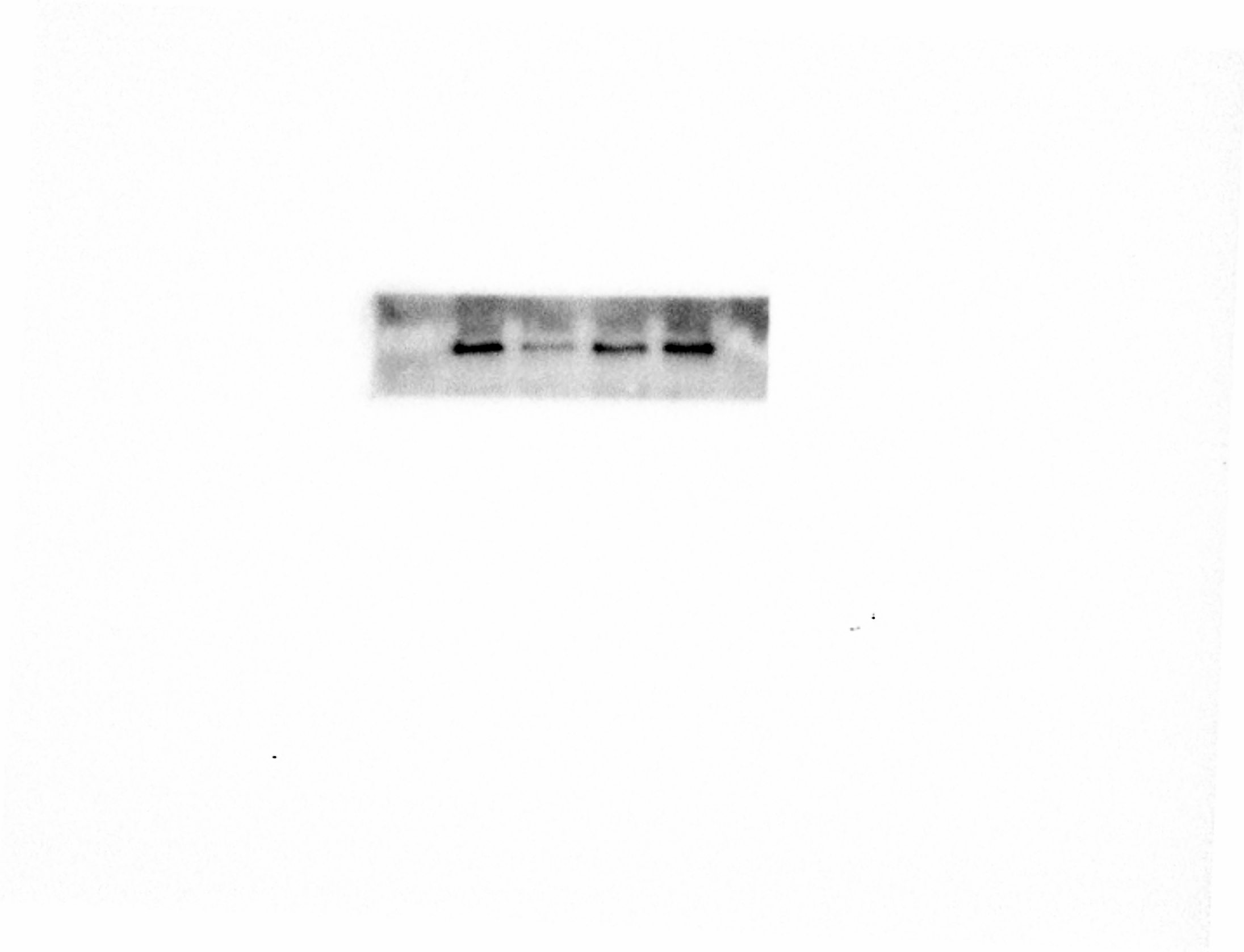

Supplement: Supplementary file 1 [file DataSheet1.zip › figure6/Fig6.A-western bolt/Cy-nrf.png]

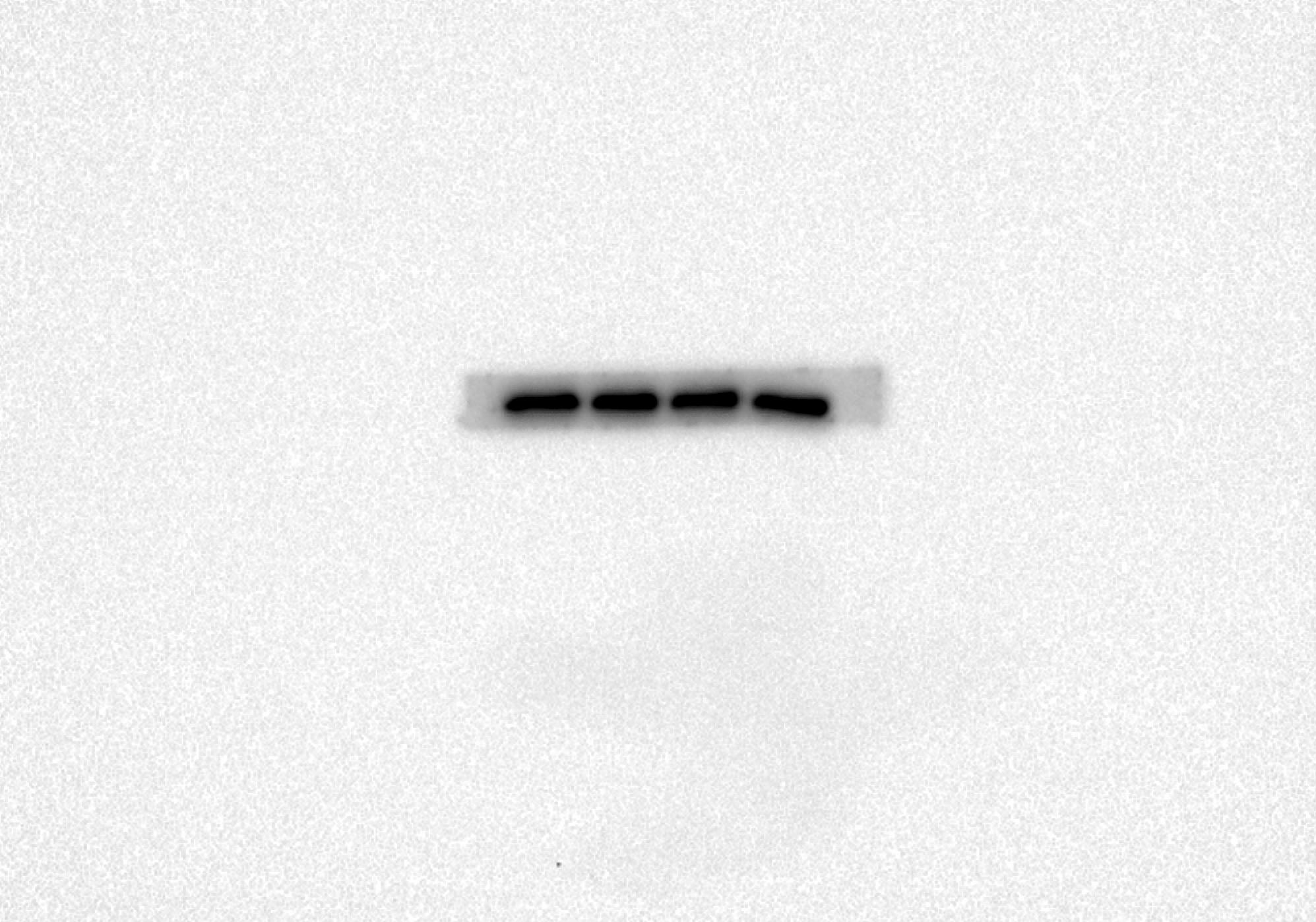

Supplement: Supplementary file 1 [file DataSheet1.zip › figure6/Fig6.A-western bolt/GAPDH.png]

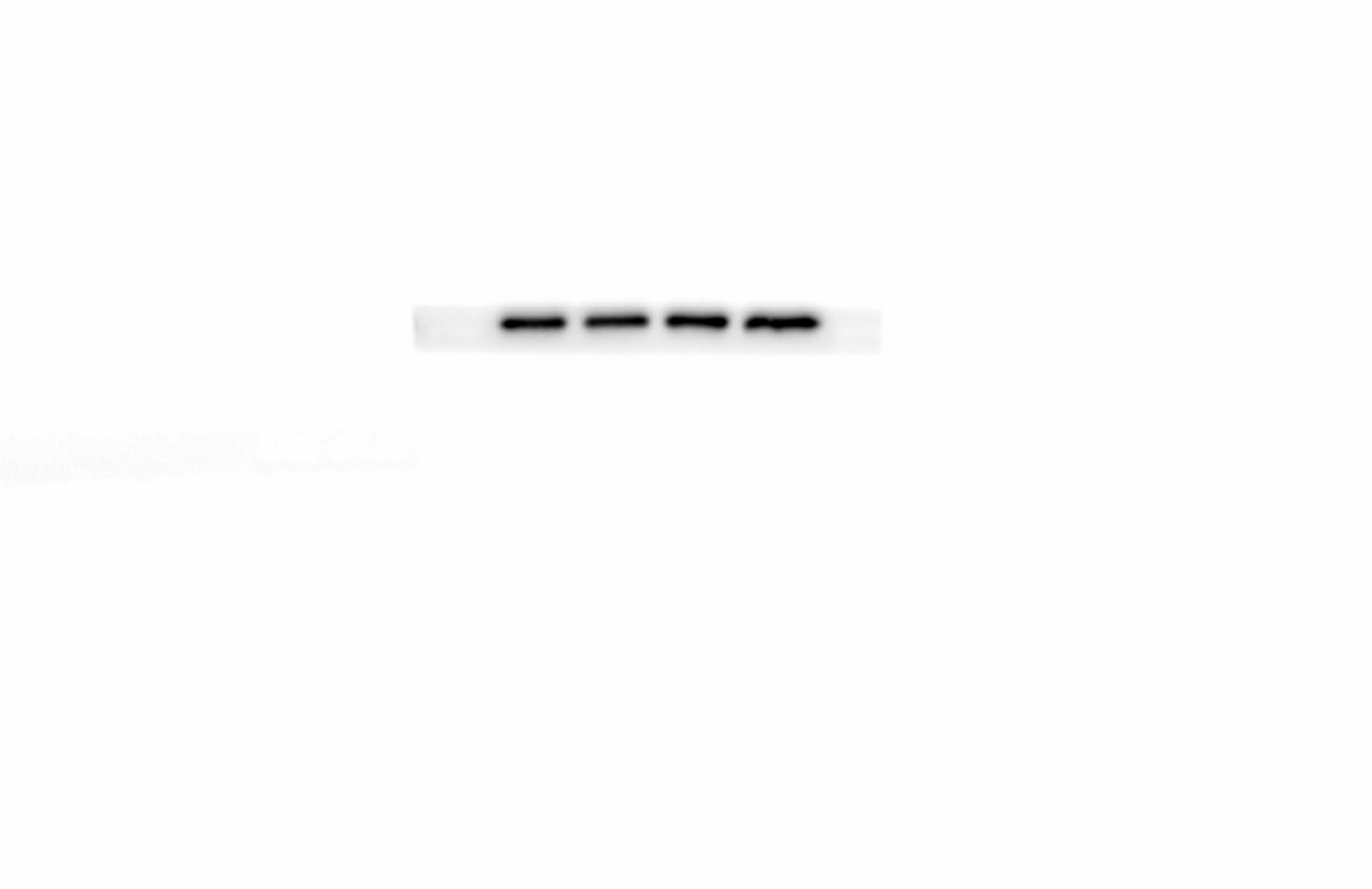

Supplement: Supplementary file 1 [file DataSheet1.zip › figure6/Fig6.A-western bolt/Lamin B.png]

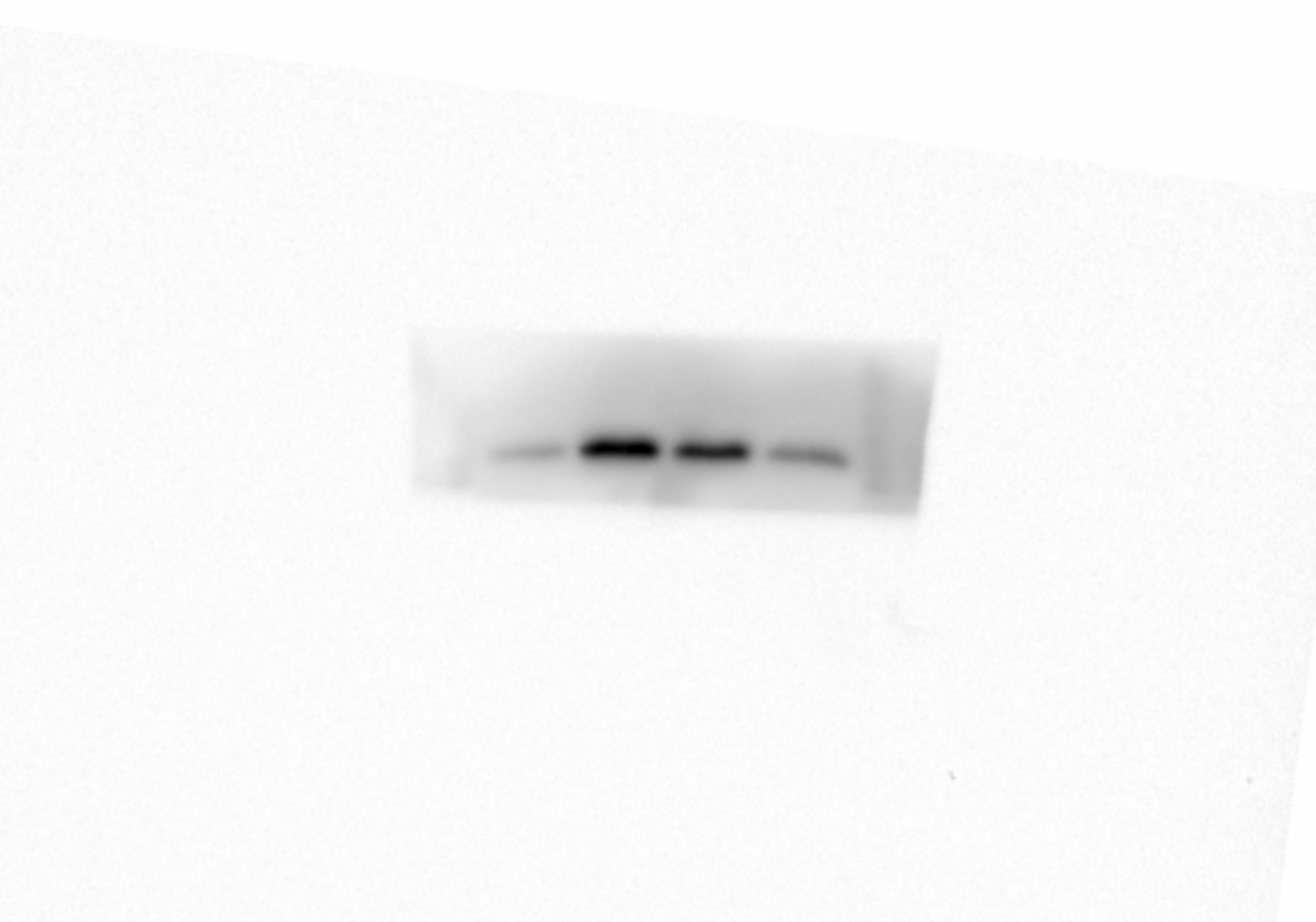

Supplement: Supplementary file 1 [file DataSheet1.zip › figure6/Fig6.A-western bolt/Nu-Nrf.png]

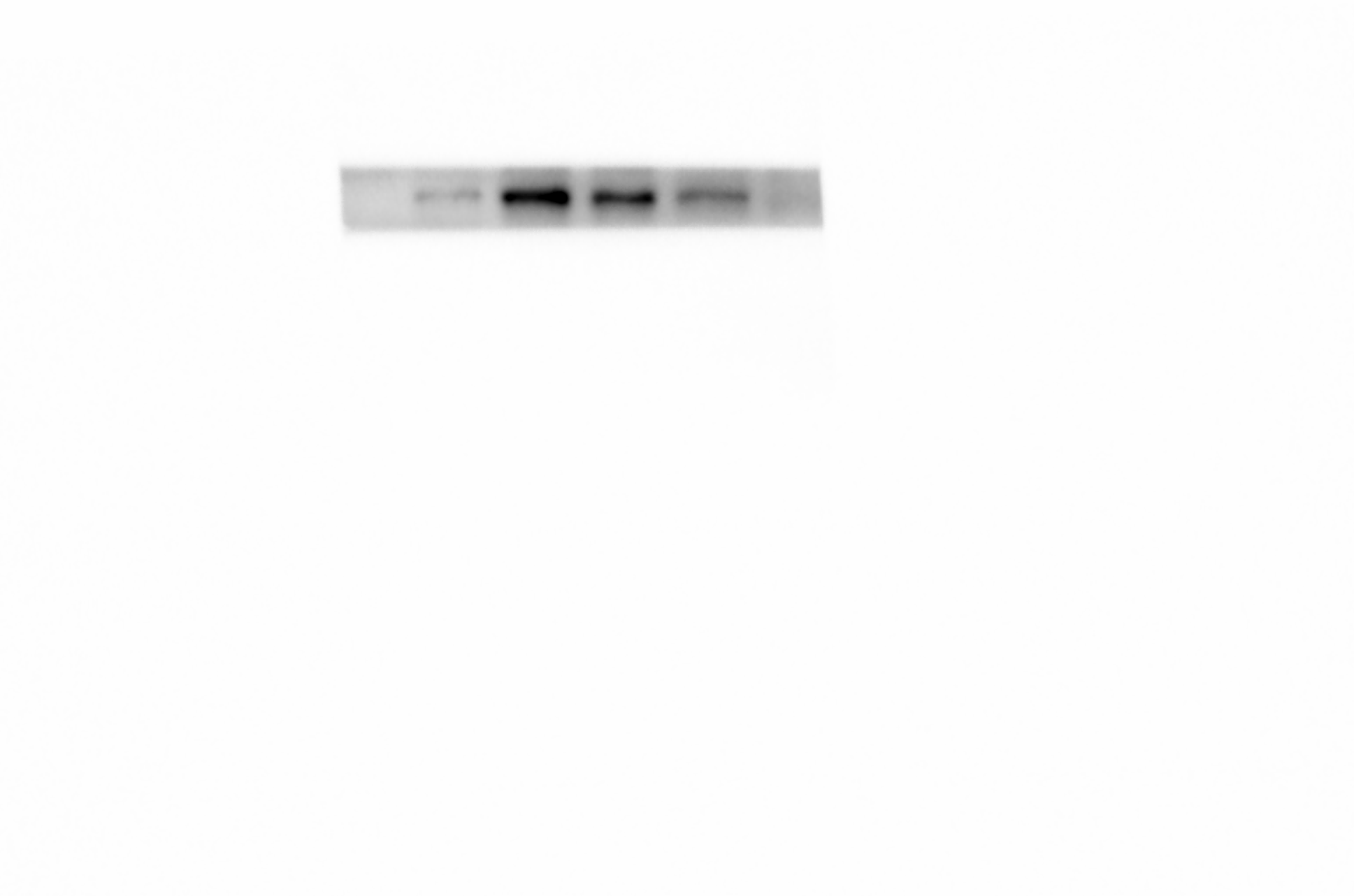

Supplement: Supplementary file 1 [file DataSheet1.zip › figure6/Fig6.A-western bolt/p-AKT.png]

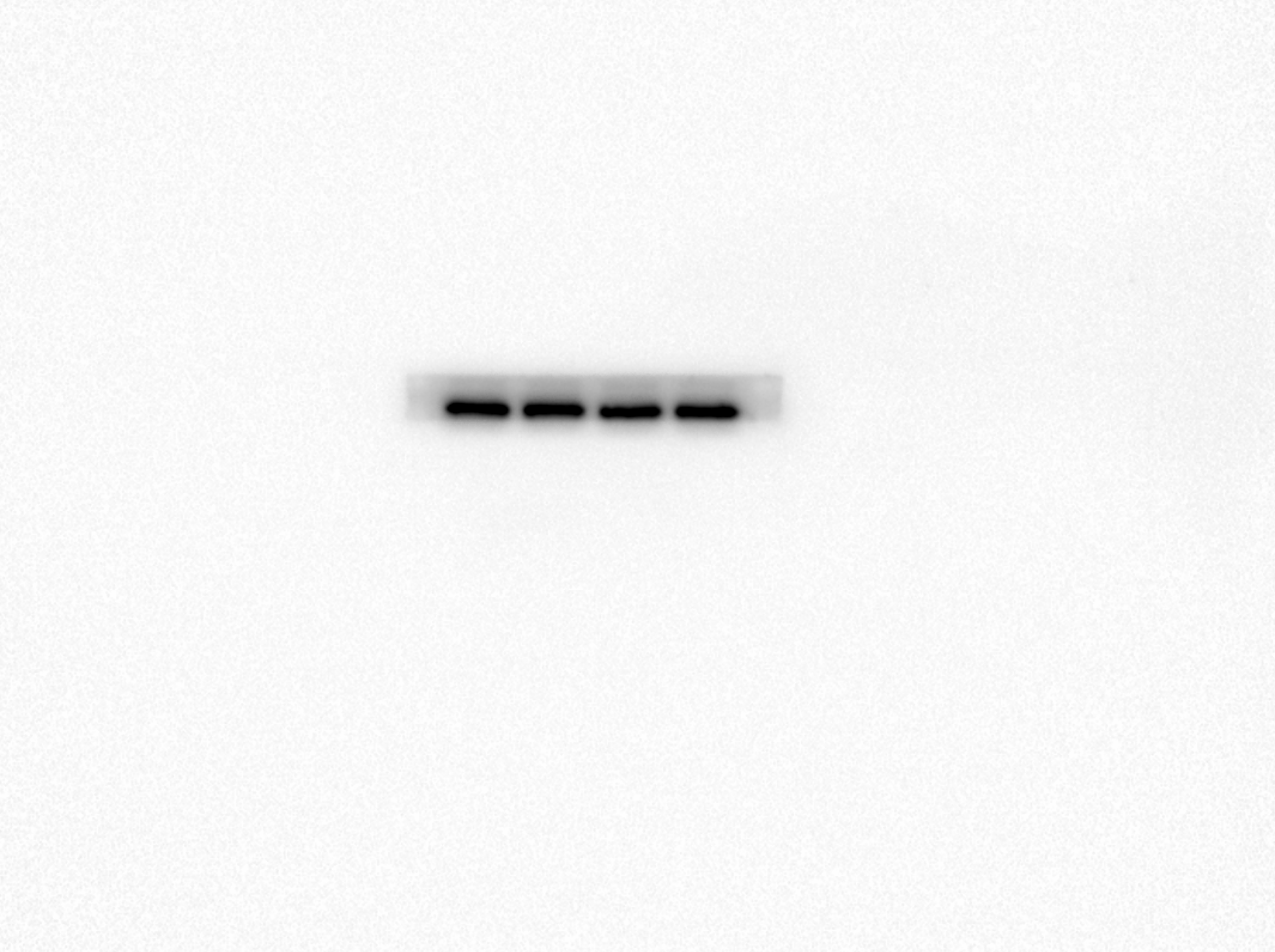

Supplement: Supplementary file 1 [file DataSheet1.zip › figure7/Fig7.A-western bolt/GAPDH.png]

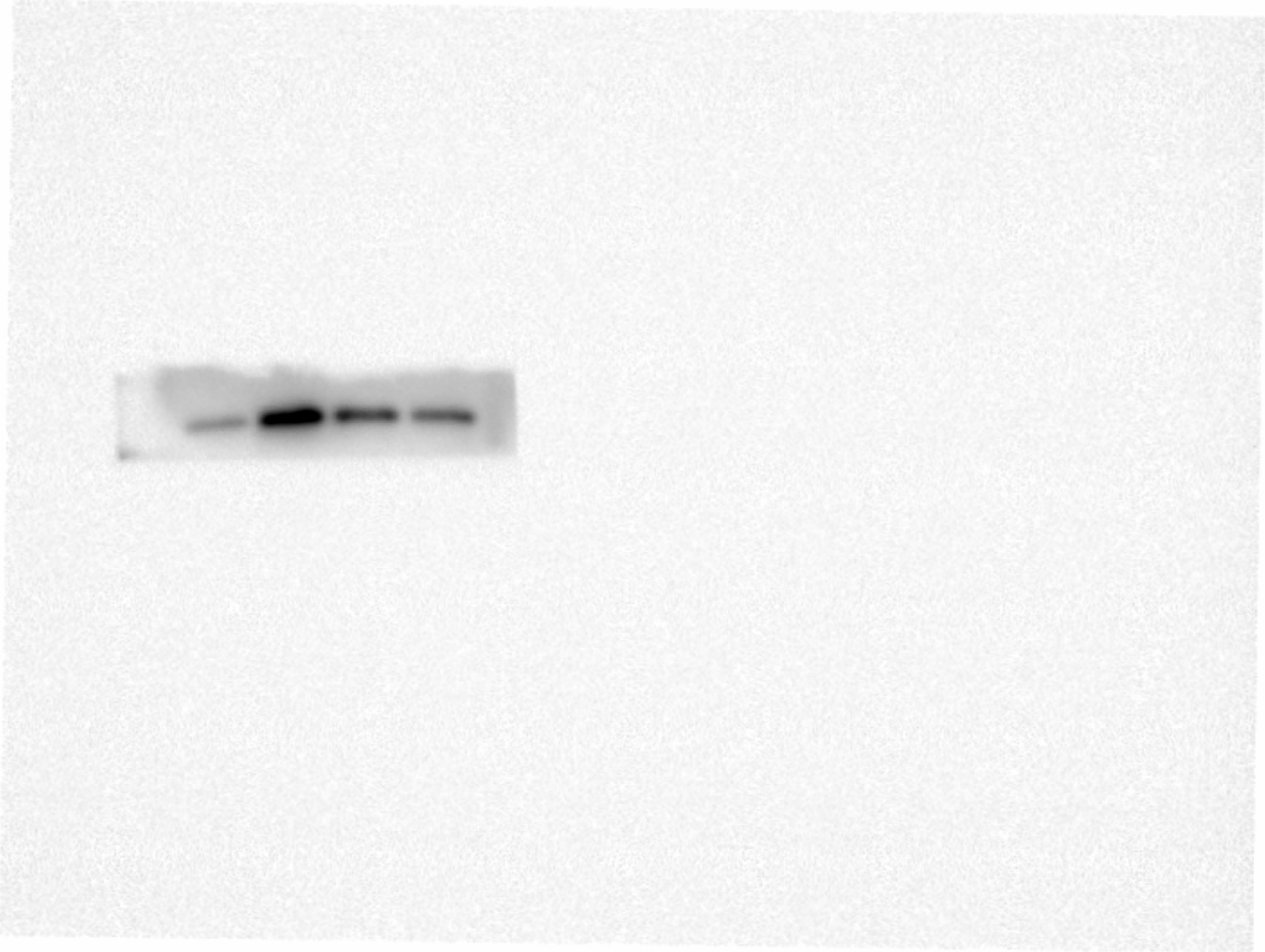

Supplement: Supplementary file 1 [file DataSheet1.zip › figure7/Fig7.A-western bolt/HO-1.png]

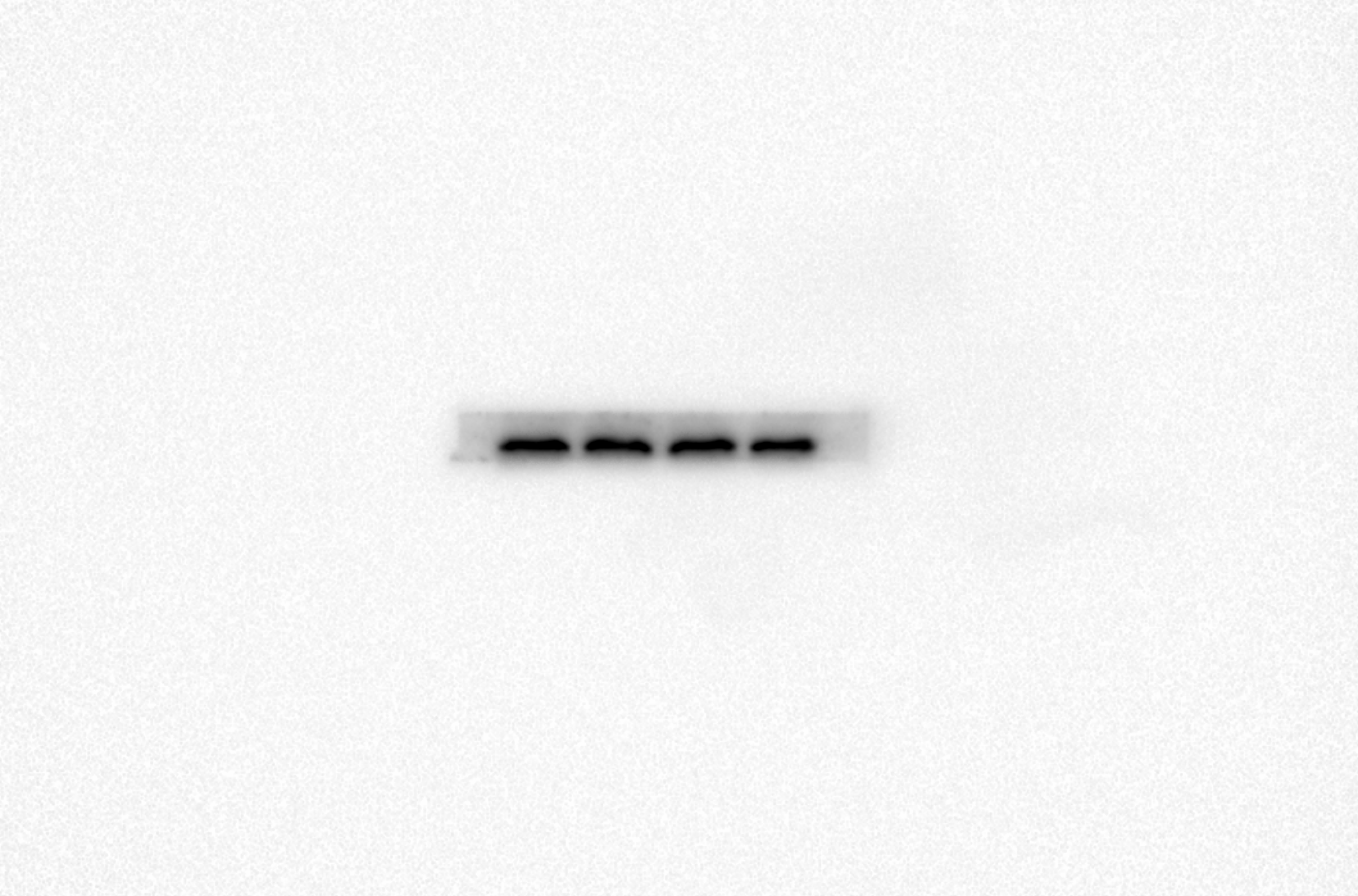

Supplement: Supplementary file 1 [file DataSheet1.zip › figure7/Fig7.A-western bolt/Lamin B.png]

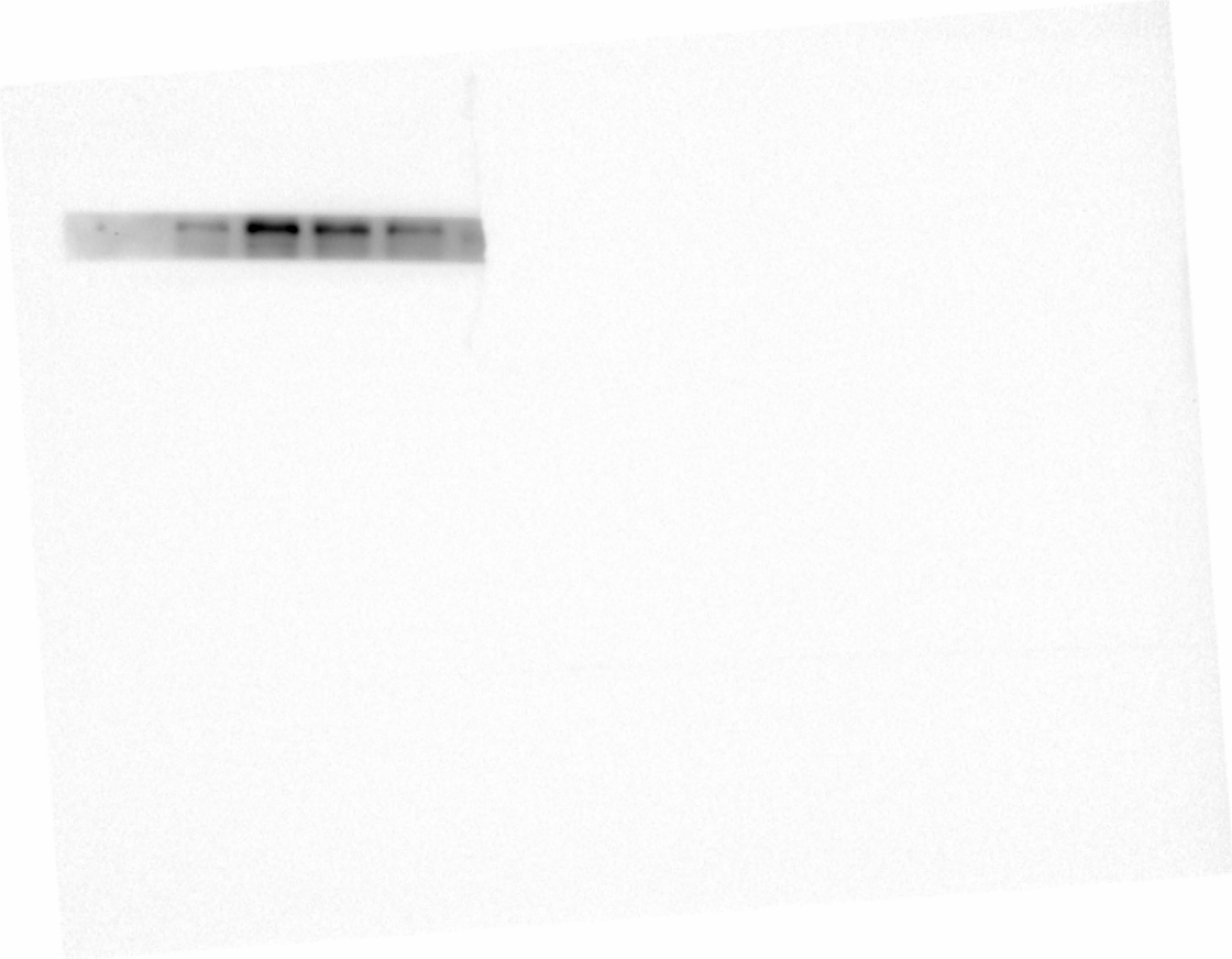

Supplement: Supplementary file 1 [file DataSheet1.zip › figure7/Fig7.A-western bolt/Nu-Nrf.png]

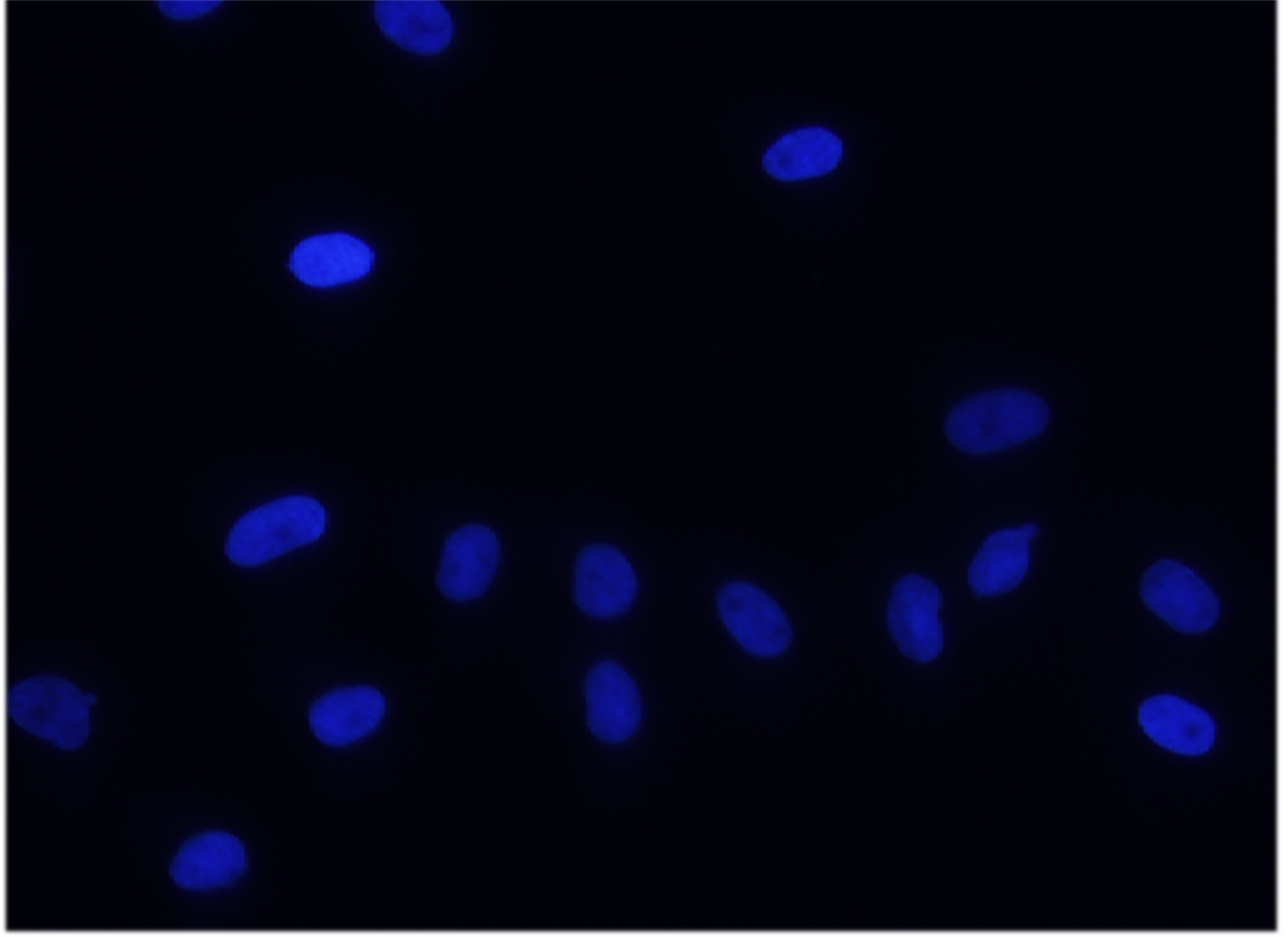

Supplement: Supplementary file 1 [file DataSheet1.zip › figure7/Fig7.C-Immunofluorescence/betulin -DAPI.jpg]

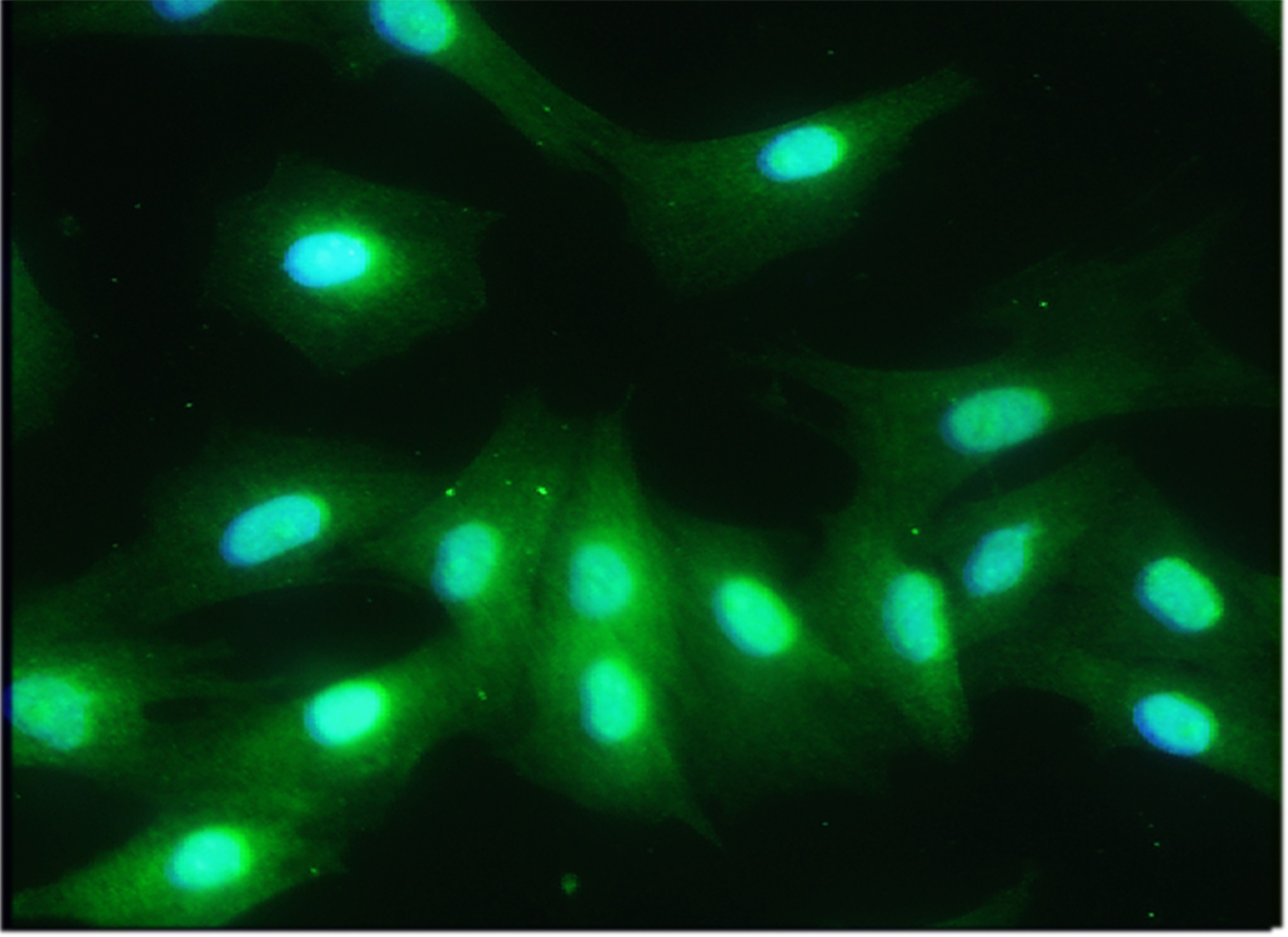

Supplement: Supplementary file 1 [file DataSheet1.zip › figure7/Fig7.C-Immunofluorescence/betulin -Merge.jpg]

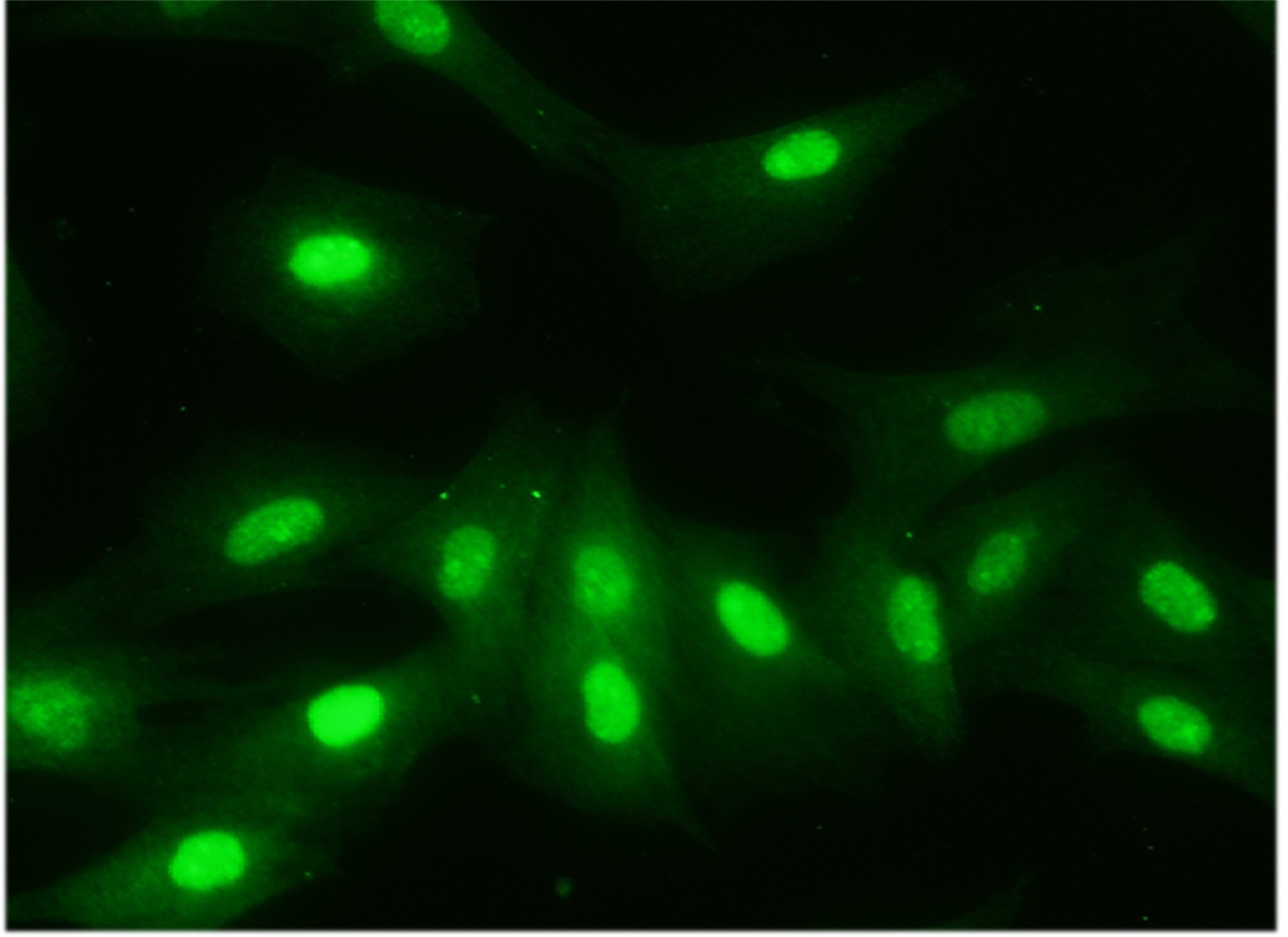

Supplement: Supplementary file 1 [file DataSheet1.zip › figure7/Fig7.C-Immunofluorescence/betulin -Nrf2.jpg]

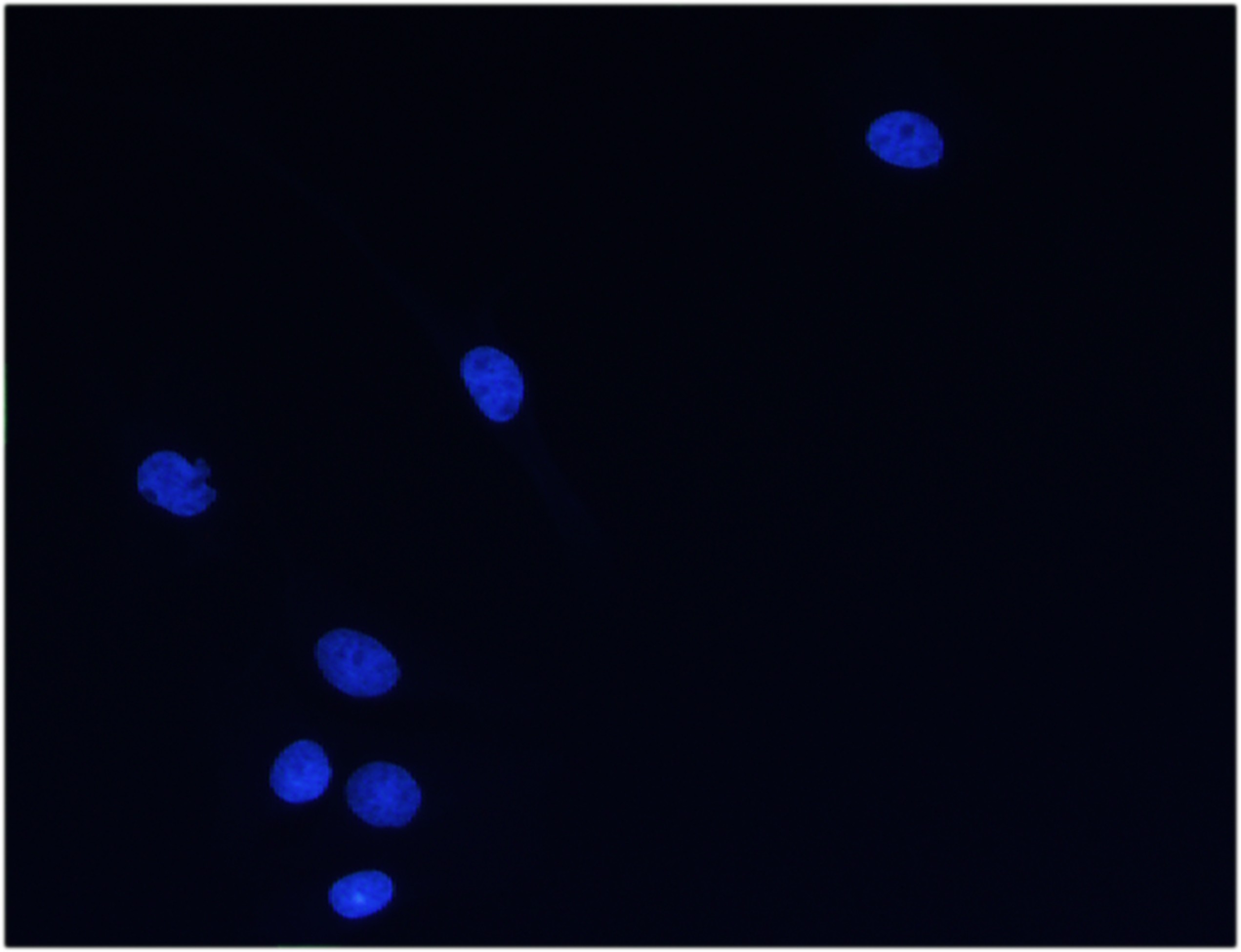

Supplement: Supplementary file 1 [file DataSheet1.zip › figure7/Fig7.C-Immunofluorescence/betulin+Ra -DAPI.jpg]

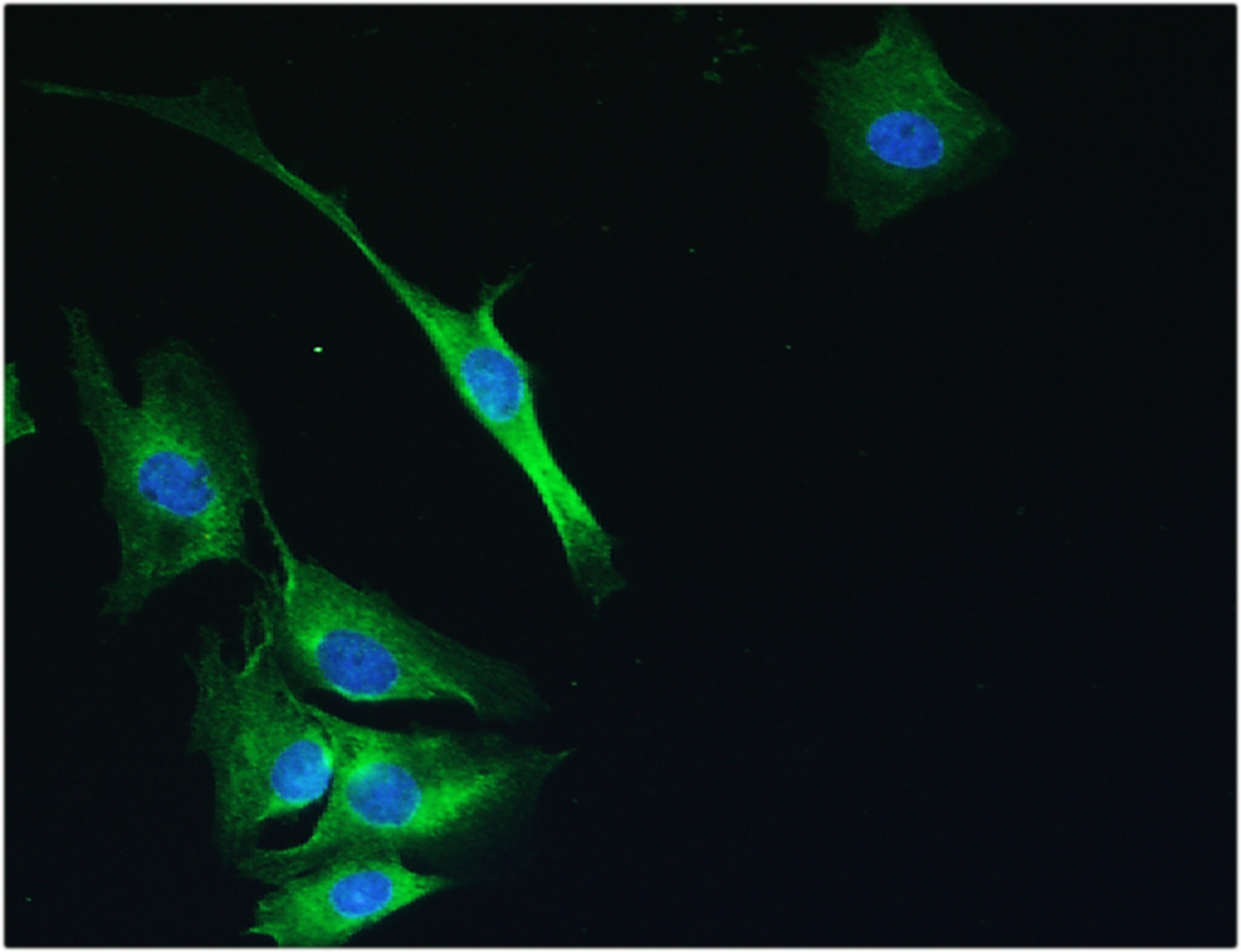

Supplement: Supplementary file 1 [file DataSheet1.zip › figure7/Fig7.C-Immunofluorescence/betulin+Ra -Merge.jpg]

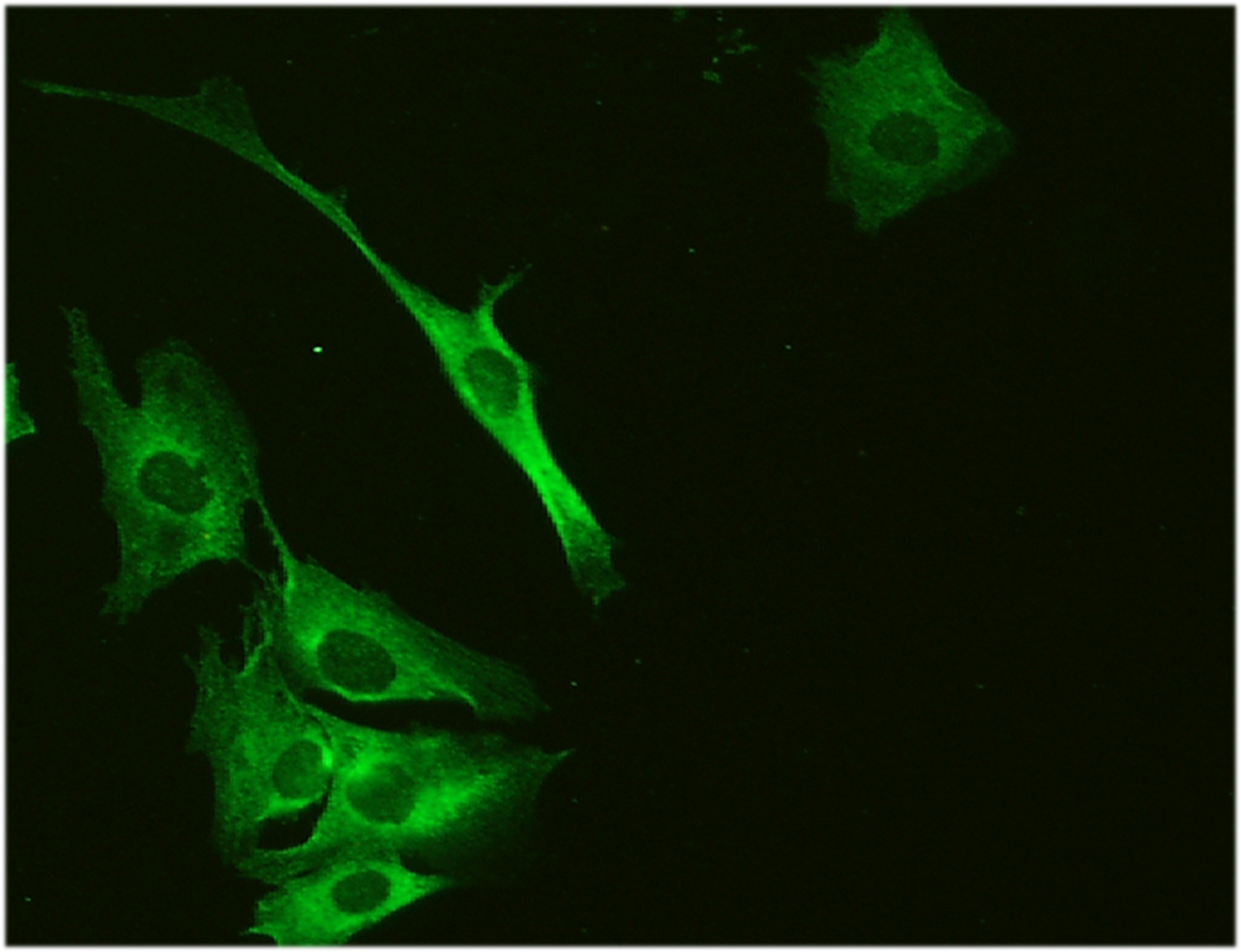

Supplement: Supplementary file 1 [file DataSheet1.zip › figure7/Fig7.C-Immunofluorescence/betulin+Ra -Nrf2.jpg]

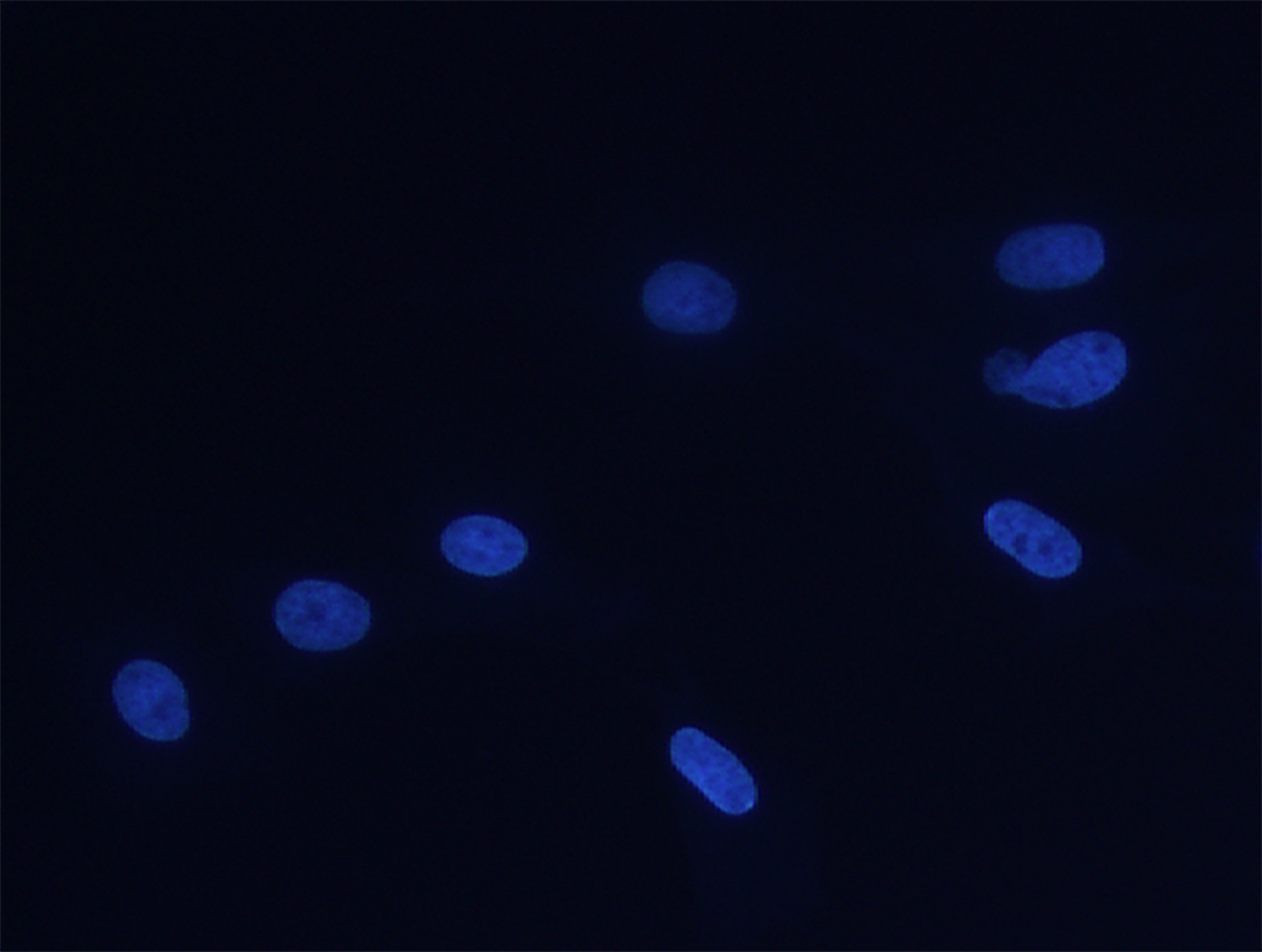

Supplement: Supplementary file 1 [file DataSheet1.zip › figure7/Fig7.C-Immunofluorescence/control -DAPI.jpg]

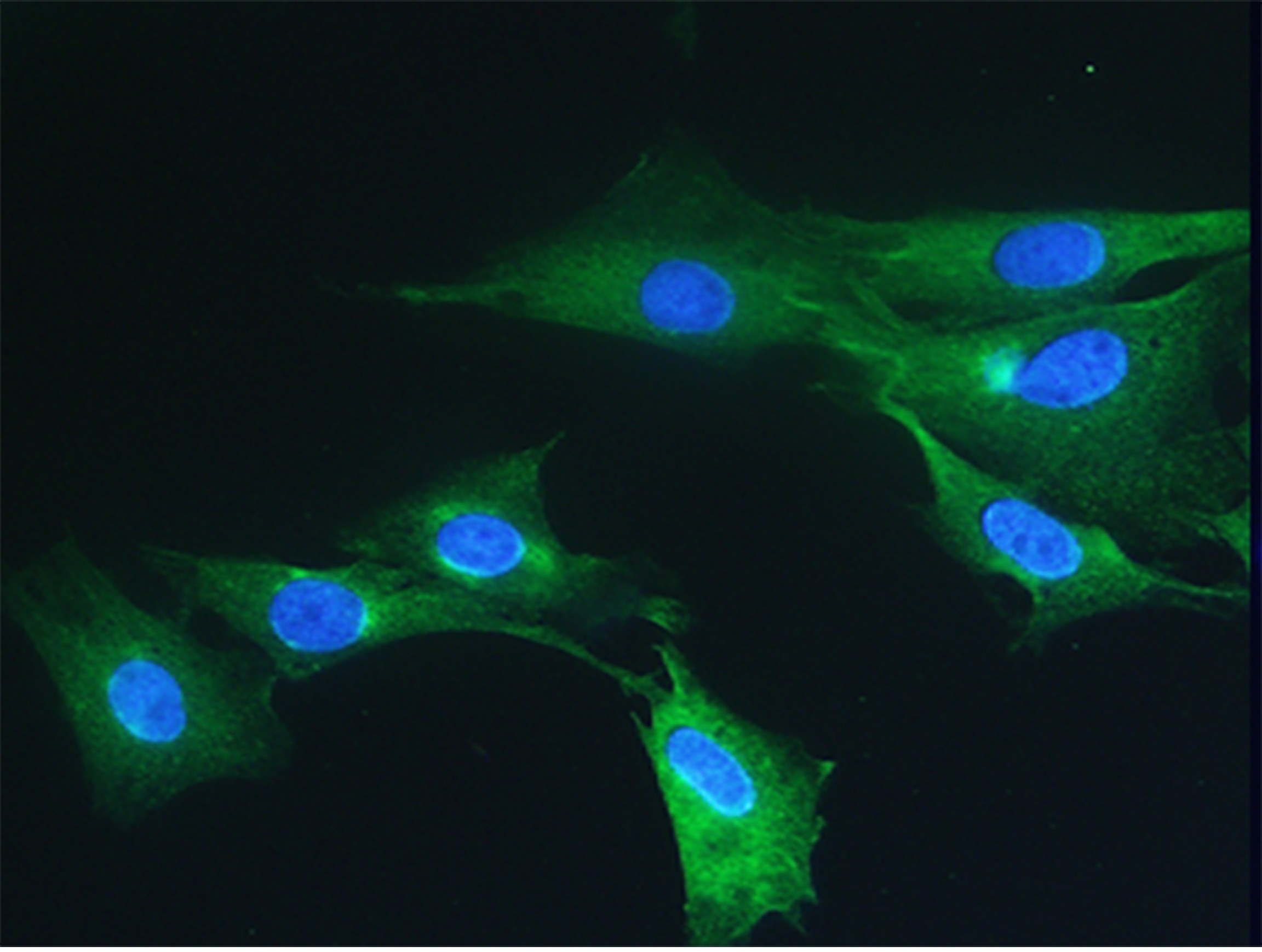

Supplement: Supplementary file 1 [file DataSheet1.zip › figure7/Fig7.C-Immunofluorescence/control -Merge.jpg]

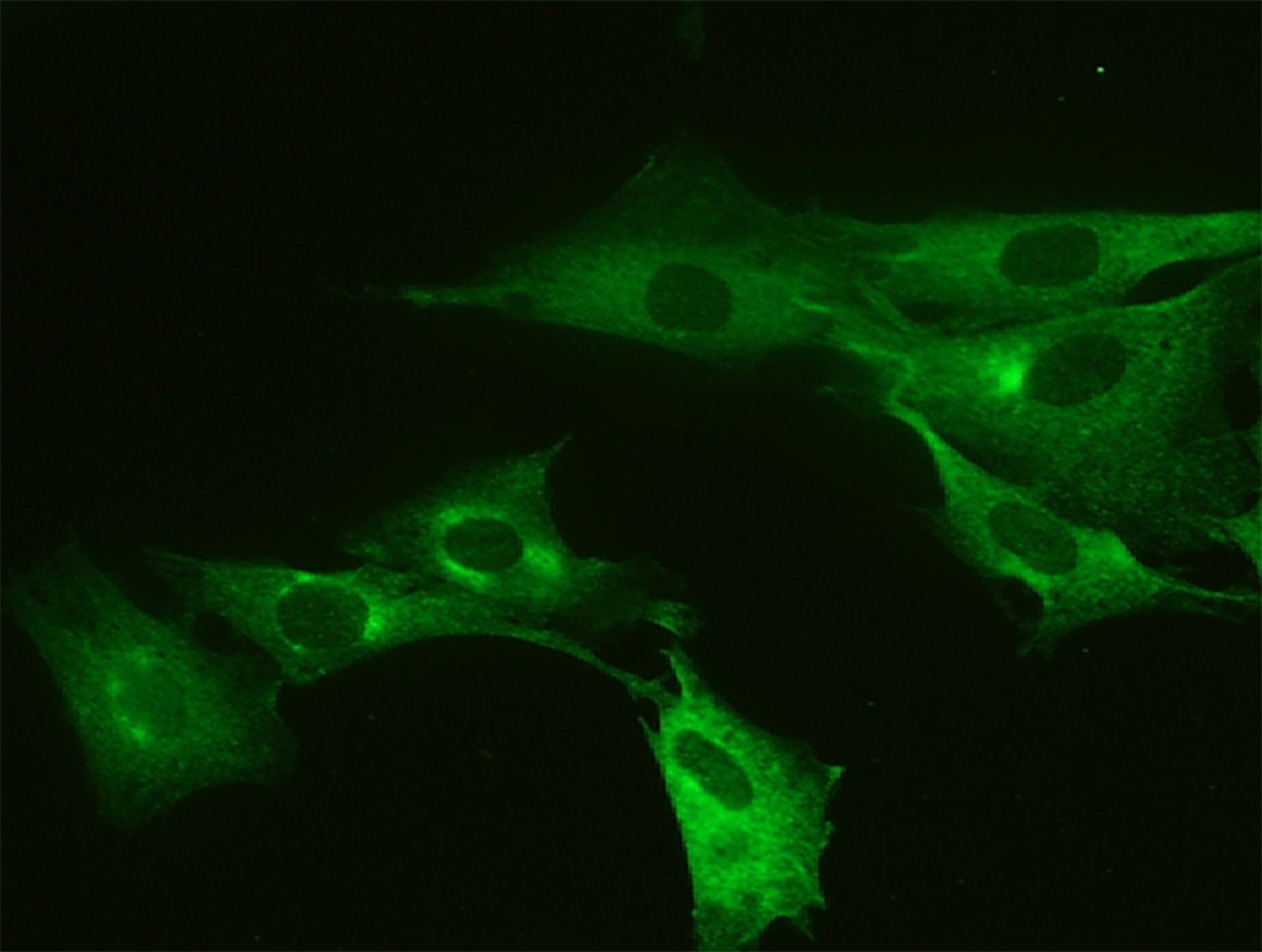

Supplement: Supplementary file 1 [file DataSheet1.zip › figure7/Fig7.C-Immunofluorescence/control -Nrf2.jpg]

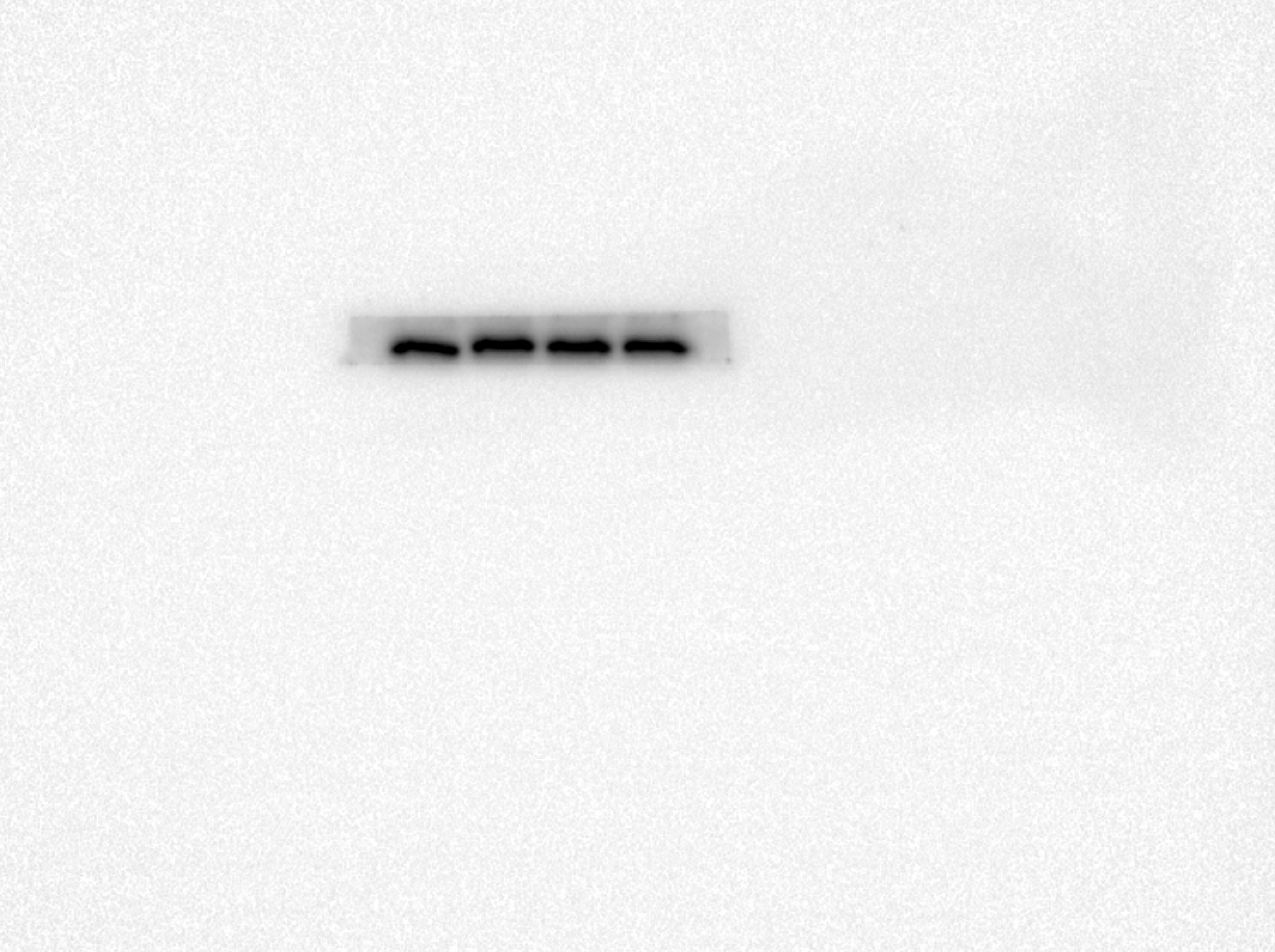

Supplement: Supplementary file 1 [file DataSheet1.zip › figure8/western bolt/A/GAPDH.png]

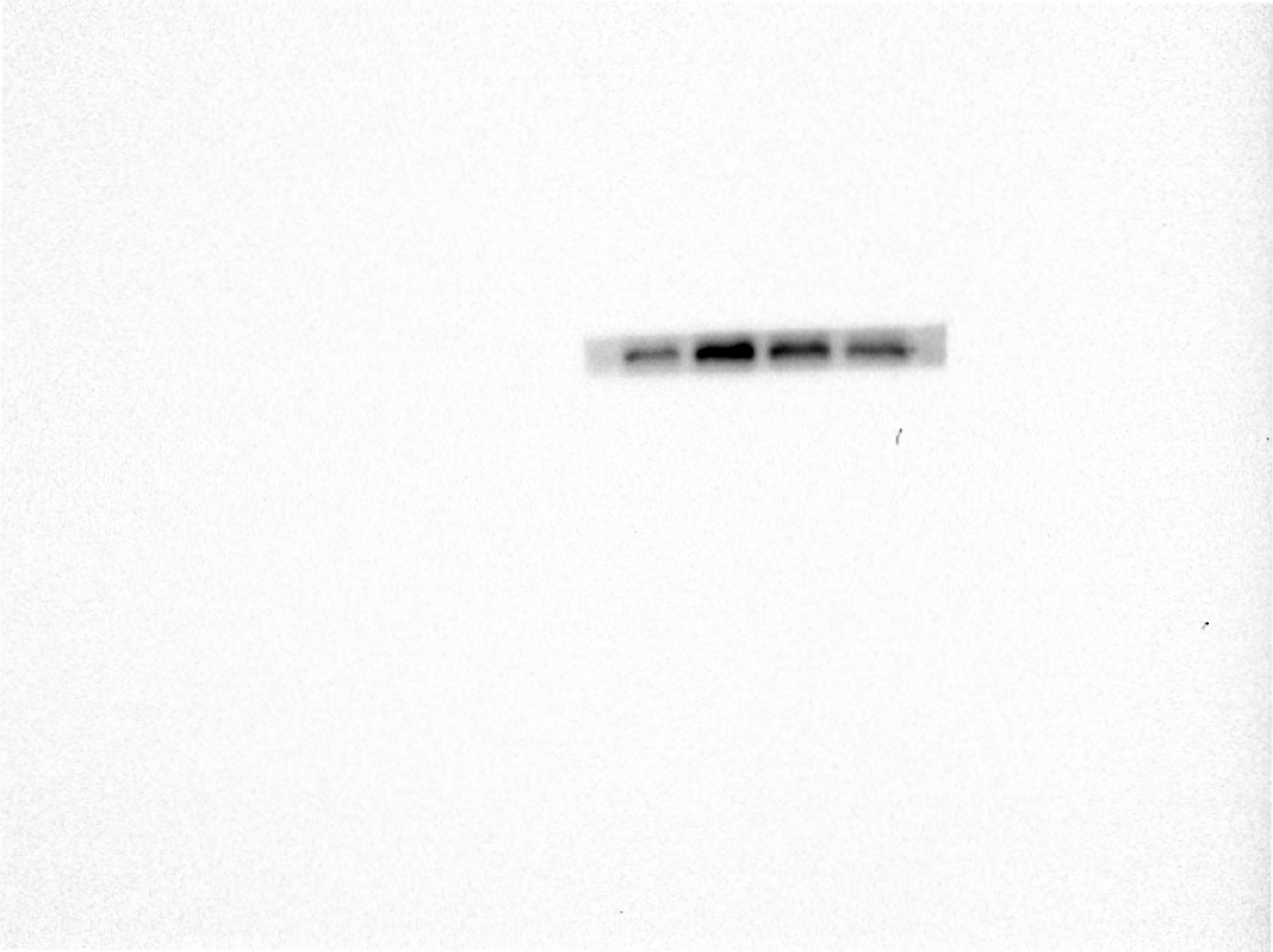

Supplement: Supplementary file 1 [file DataSheet1.zip › figure8/western bolt/A/HO-1.png]

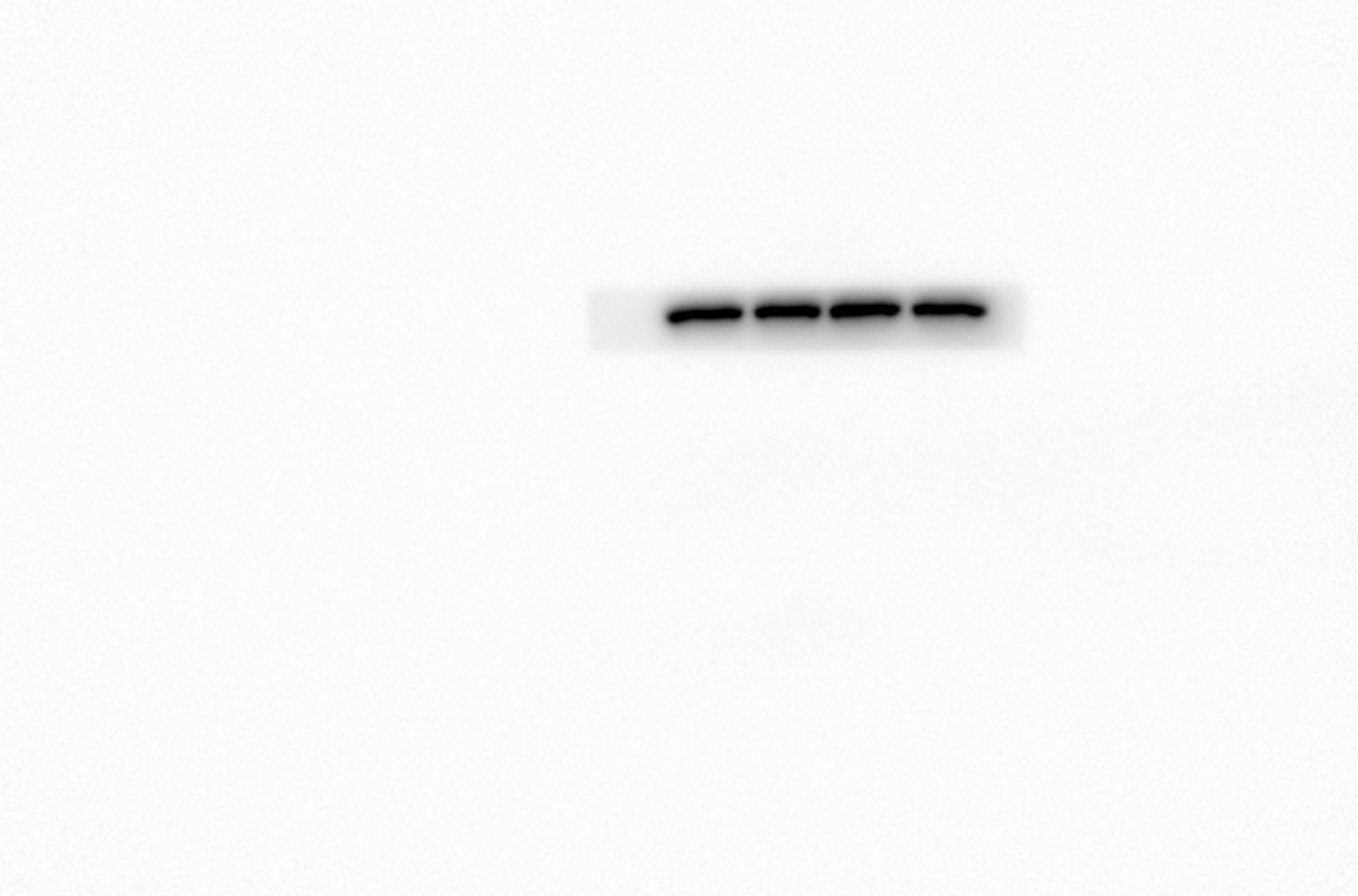

Supplement: Supplementary file 1 [file DataSheet1.zip › figure8/western bolt/C/GAPDH-2.png]

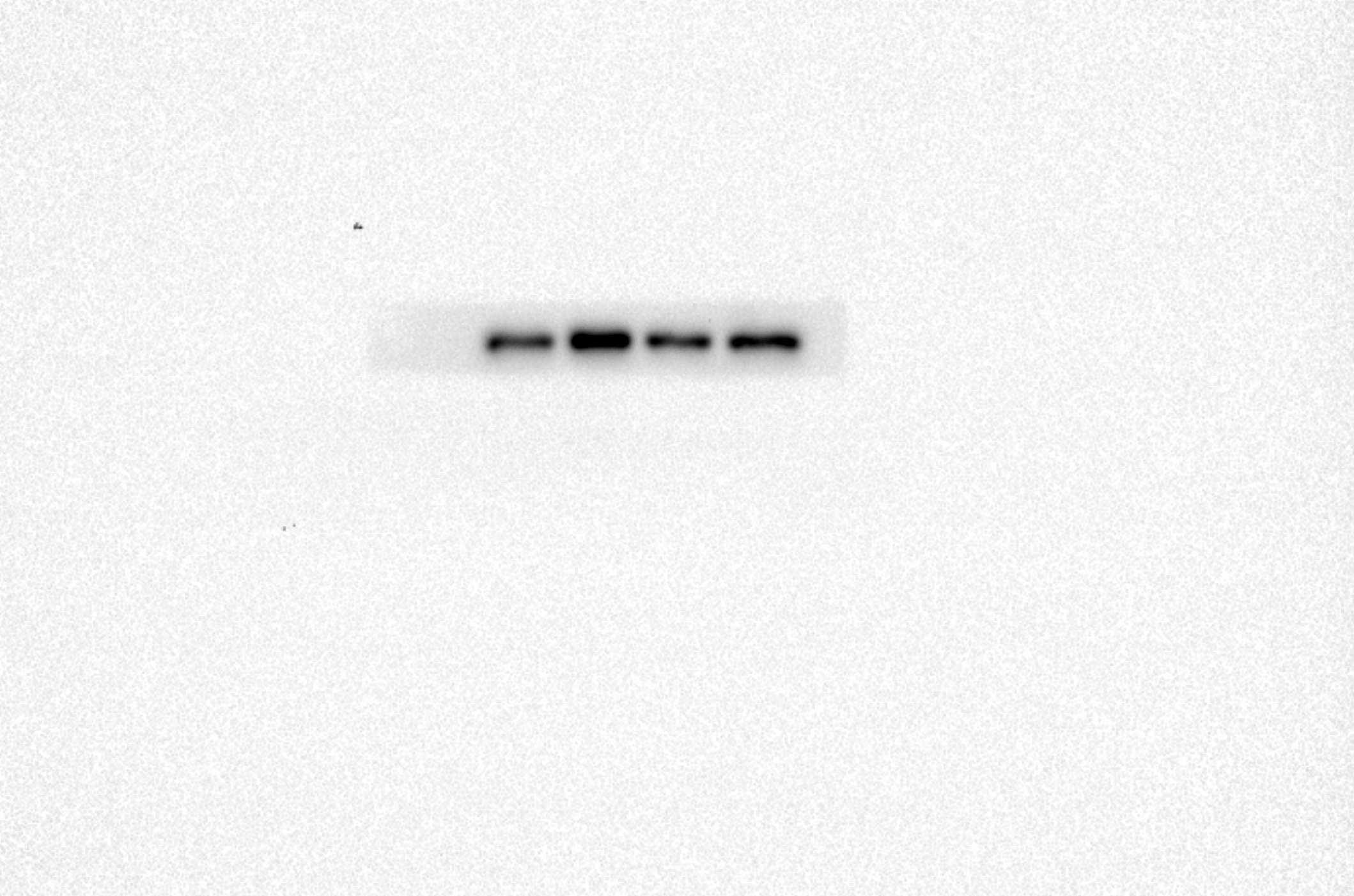

Supplement: Supplementary file 1 [file DataSheet1.zip › figure8/western bolt/C/P-P65.png]

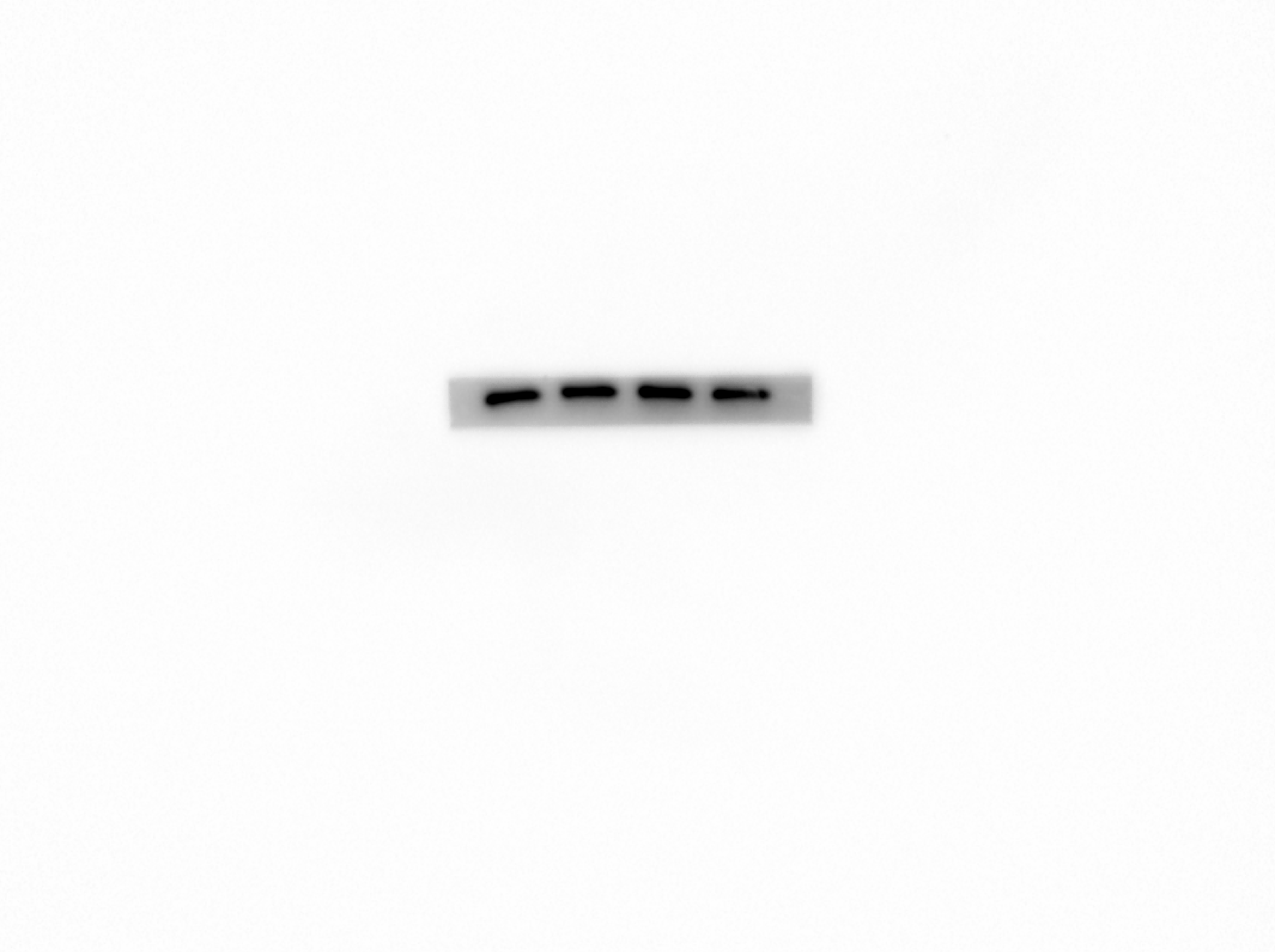

Supplement: Supplementary file 1 [file DataSheet1.zip › figure8/western bolt/C/P65.png]

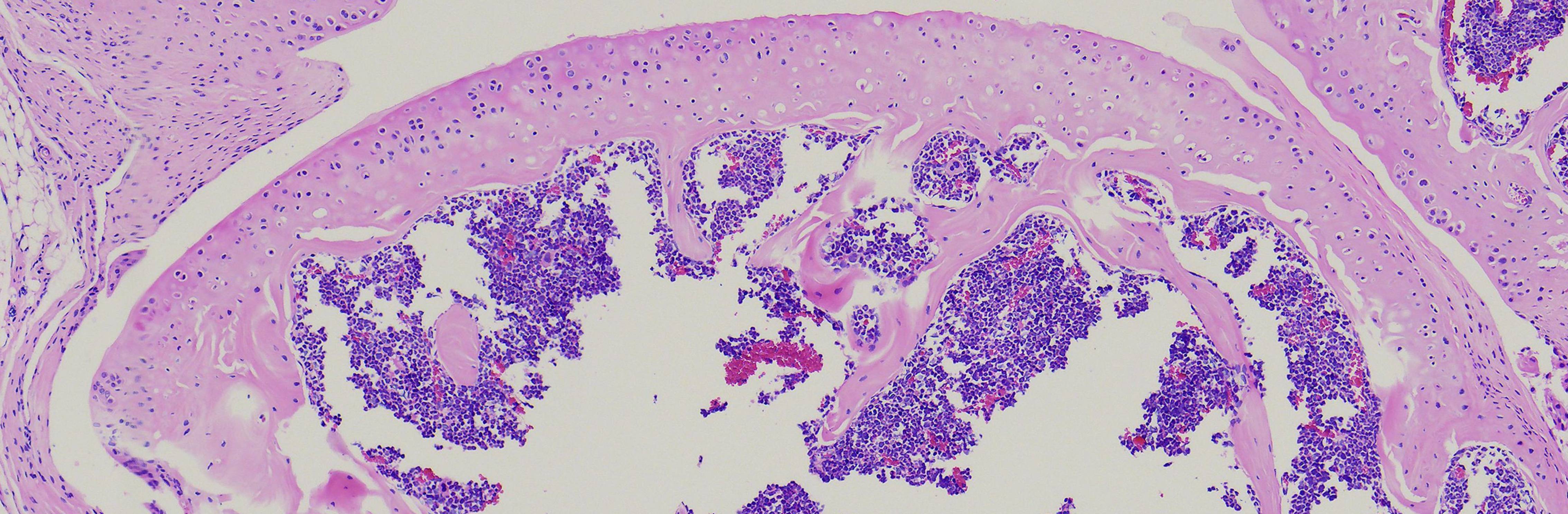

Supplement: Supplementary file 1 [file DataSheet1.zip › figure9/HE/DMM+Betulin.jpg]

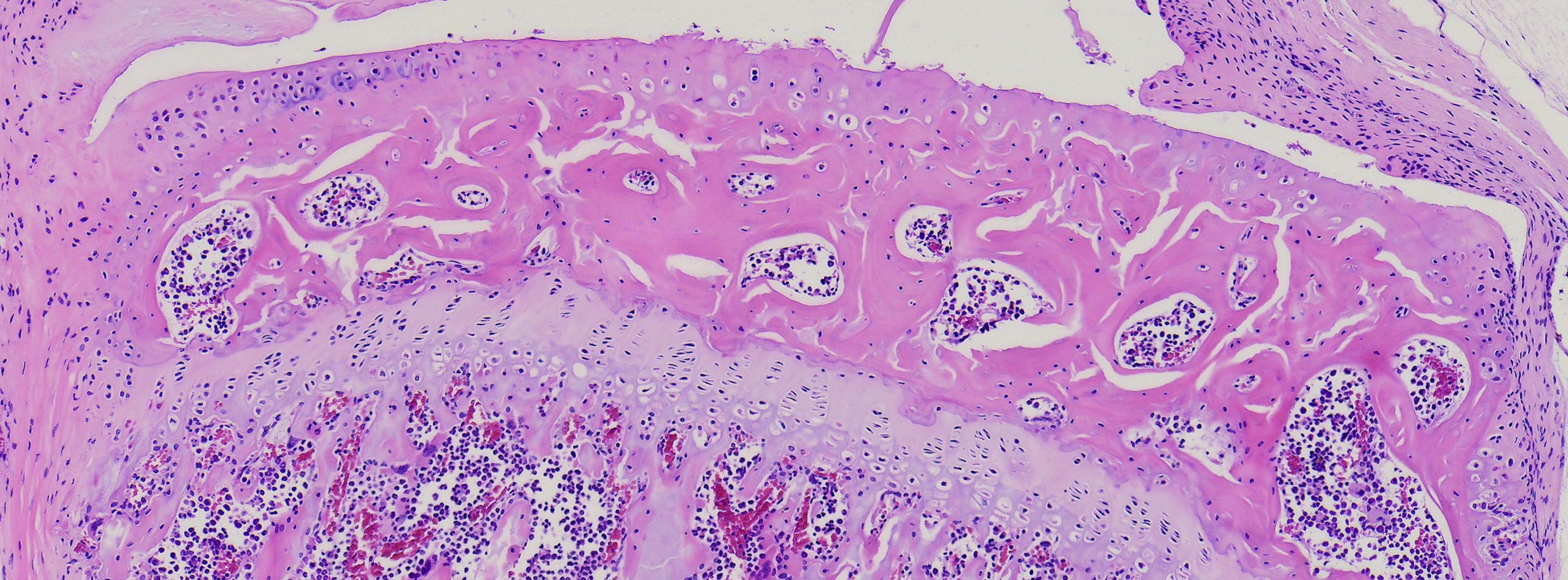

Supplement: Supplementary file 1 [file DataSheet1.zip › figure9/HE/DMM.jpg]

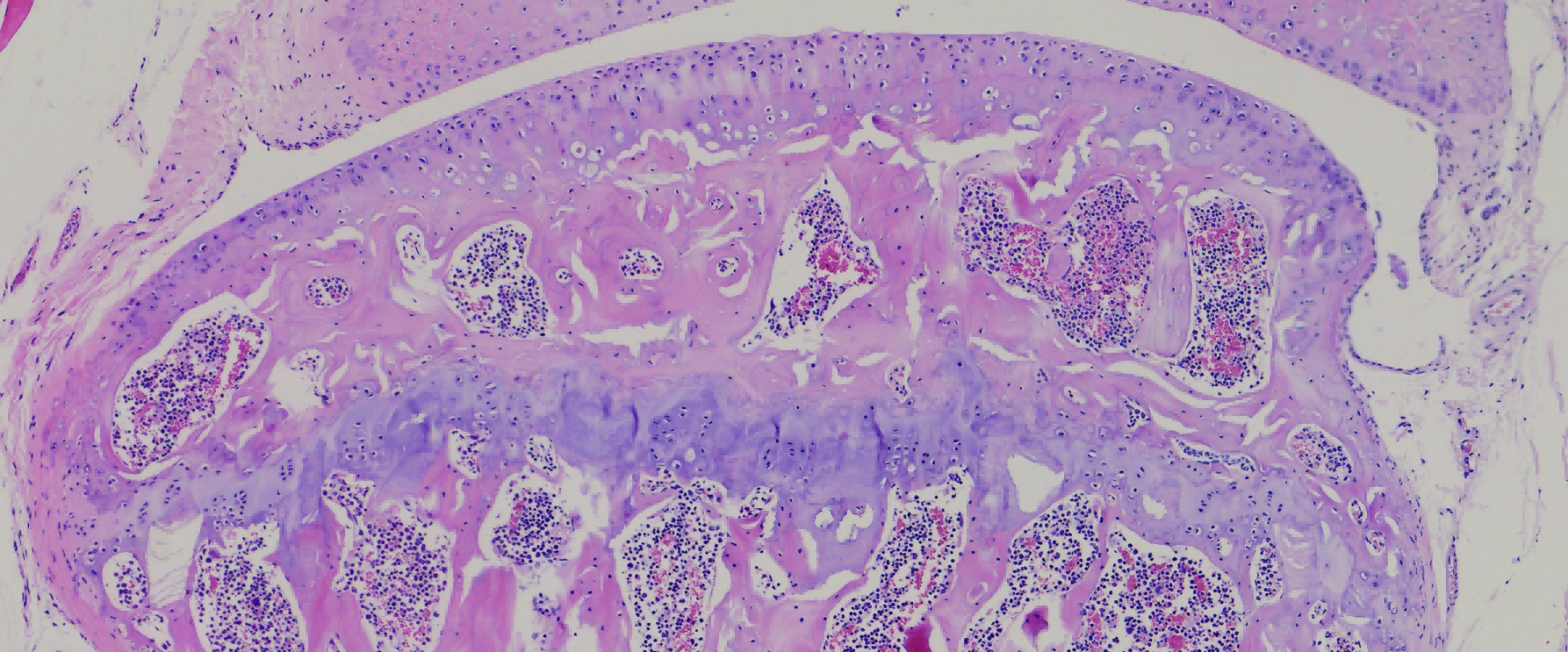

Supplement: Supplementary file 1 [file DataSheet1.zip › figure9/HE/Sham.png]

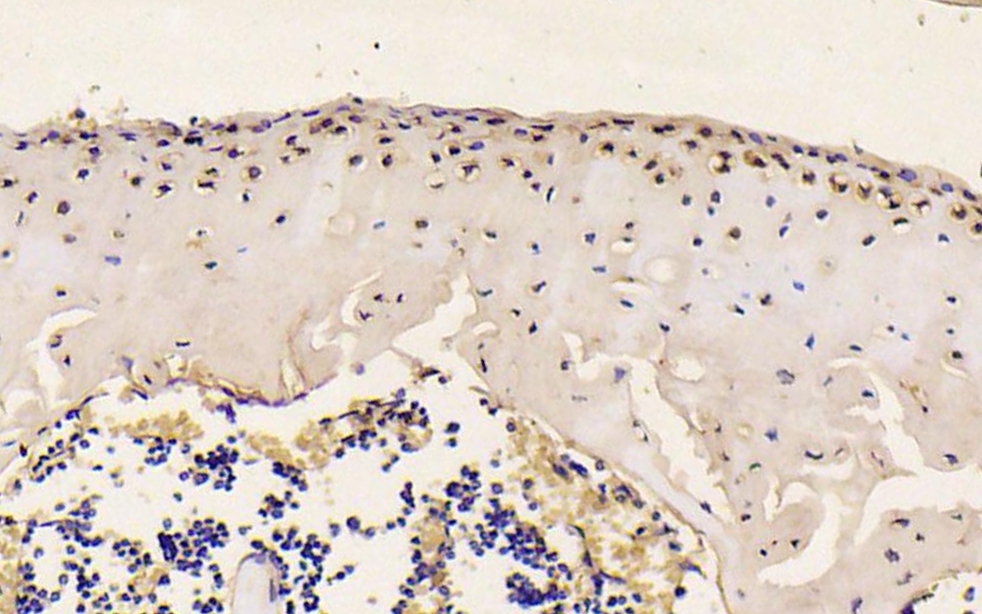

Supplement: Supplementary file 1 [file DataSheet1.zip › figure9/immunohischemistry/IL-6/IL-6-DMM+Betulin.jpg]

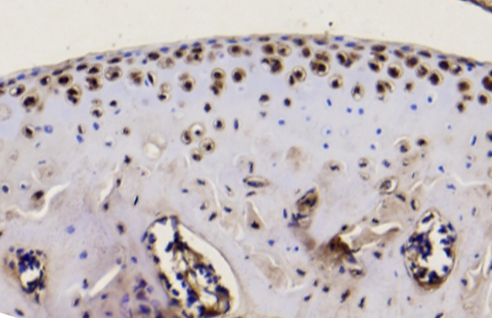

Supplement: Supplementary file 1 [file DataSheet1.zip › figure9/immunohischemistry/IL-6/IL-6-DMM.jpg]

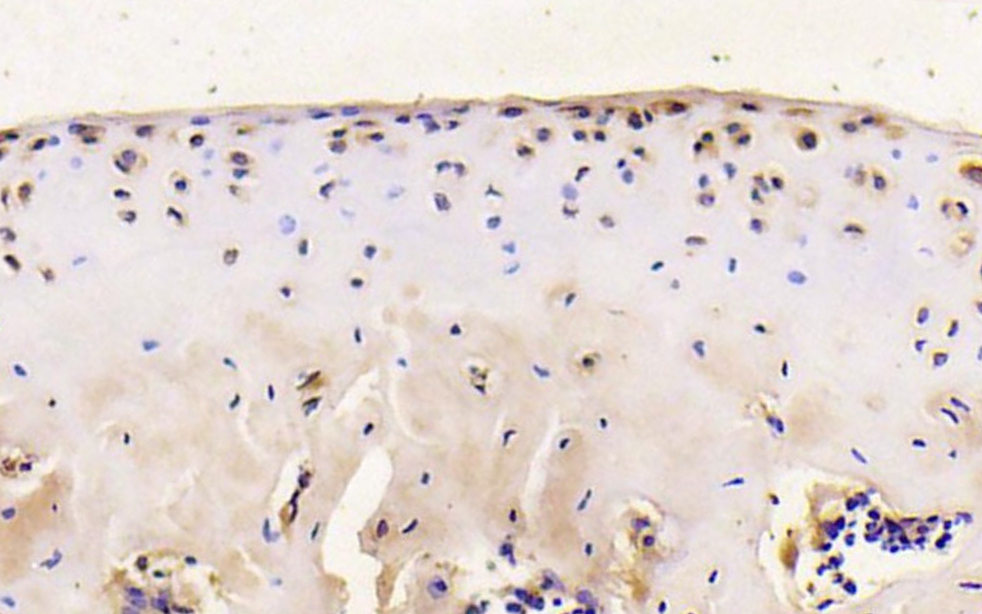

Supplement: Supplementary file 1 [file DataSheet1.zip › figure9/immunohischemistry/IL-6/IL-6-Sham.jpg]

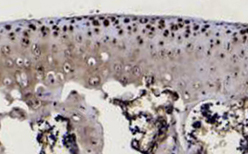

Supplement: Supplementary file 1 [file DataSheet1.zip › figure9/immunohischemistry/Nrf2/Nrf2-DMM+Betulin7.jpg]

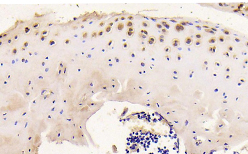

Supplement: Supplementary file 1 [file DataSheet1.zip › figure9/immunohischemistry/Nrf2/Nrf2-DMM.jpg]

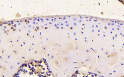

Supplement: Supplementary file 1 [file DataSheet1.zip › figure9/immunohischemistry/Nrf2/Nrf2-Sham.tif]

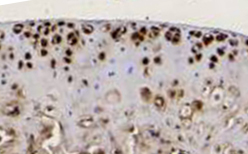

Supplement: Supplementary file 1 [file DataSheet1.zip › figure9/immunohischemistry/P-AKT/P-AKT-DMM+Betulin.jpg]

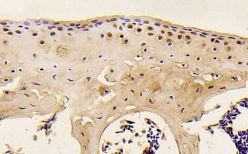

Supplement: Supplementary file 1 [file DataSheet1.zip › figure9/immunohischemistry/P-AKT/P-AKT-DMM.jpg]

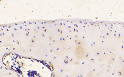

Supplement: Supplementary file 1 [file DataSheet1.zip › figure9/immunohischemistry/P-AKT/P-AKT-Sham.tif]

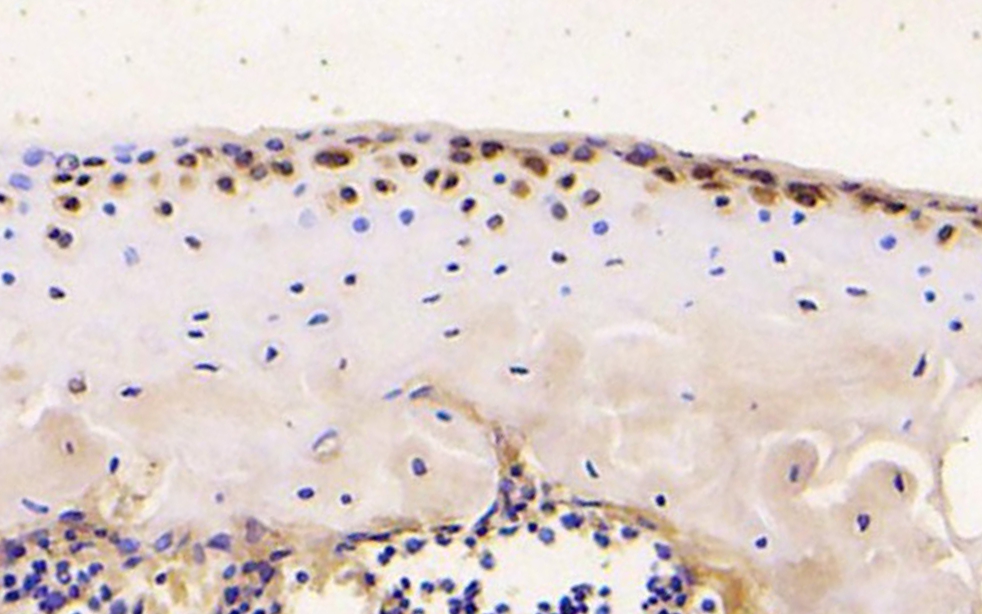

Supplement: Supplementary file 1 [file DataSheet1.zip › figure9/immunohischemistry/TNF-a/TNF-a-DMM+Betulin.jpg]

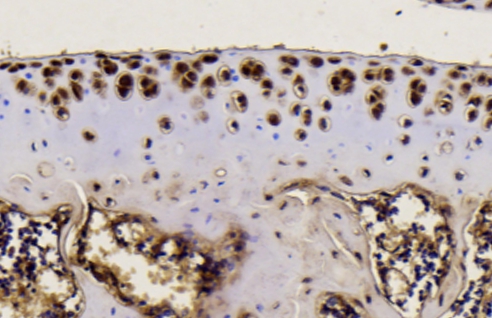

Supplement: Supplementary file 1 [file DataSheet1.zip › figure9/immunohischemistry/TNF-a/TNF-a-DMM.jpg]

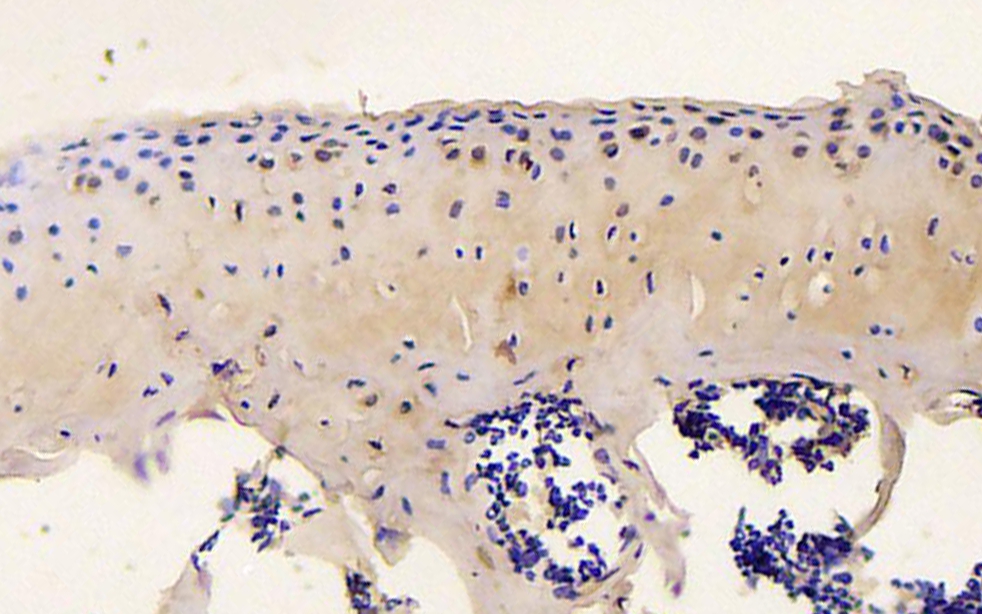

Supplement: Supplementary file 1 [file DataSheet1.zip › figure9/immunohischemistry/TNF-a/TNF-a-Sham.jpg]

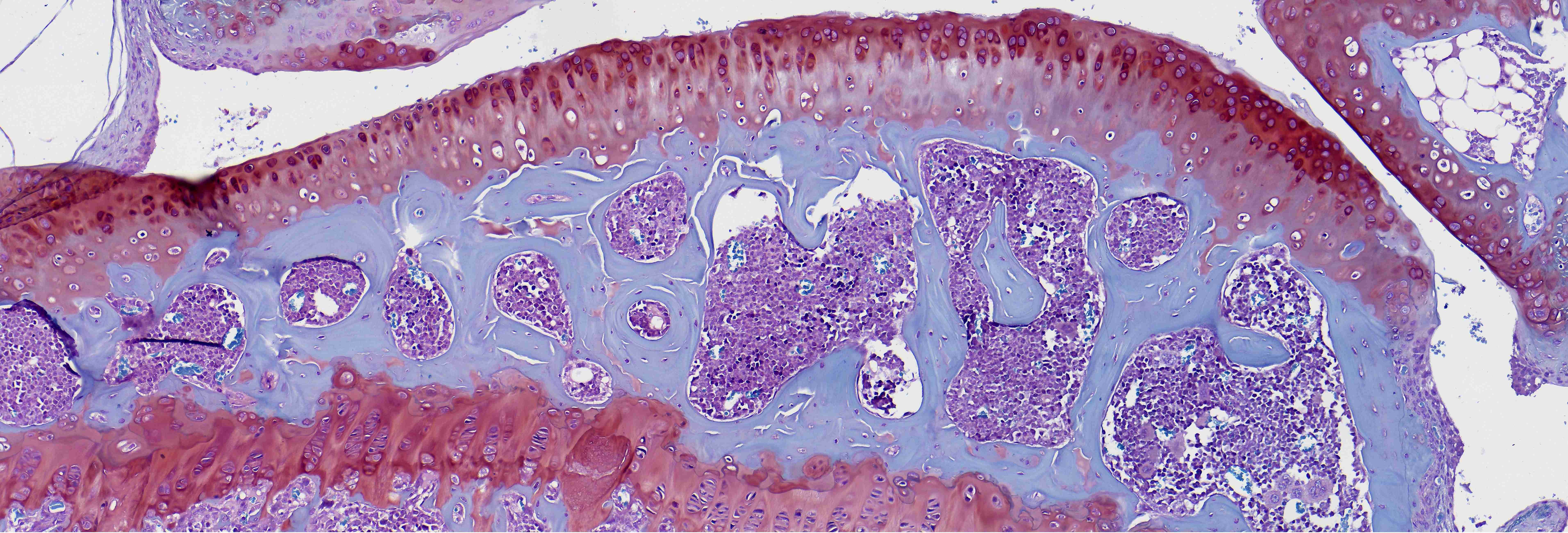

Supplement: Supplementary file 1 [file DataSheet1.zip › figure9/SO/DMM+Betulin.jpg]

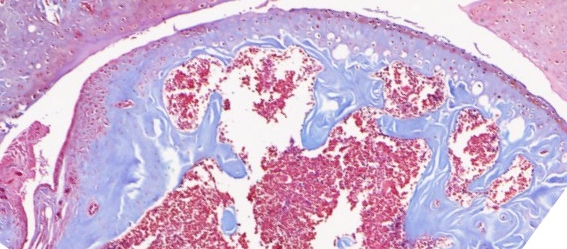

Supplement: Supplementary file 1 [file DataSheet1.zip › figure9/SO/DMM.jpg]

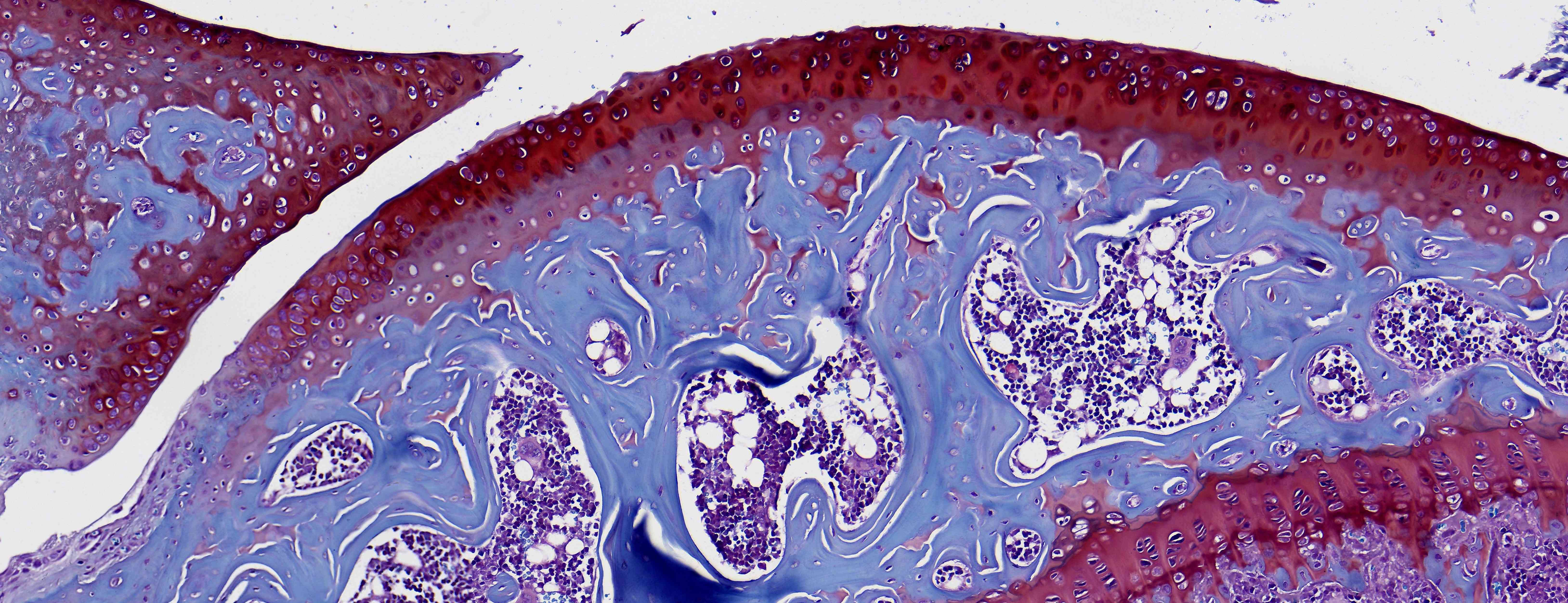

Supplement: Supplementary file 1 [file DataSheet1.zip › figure9/SO/Sham.jpg]

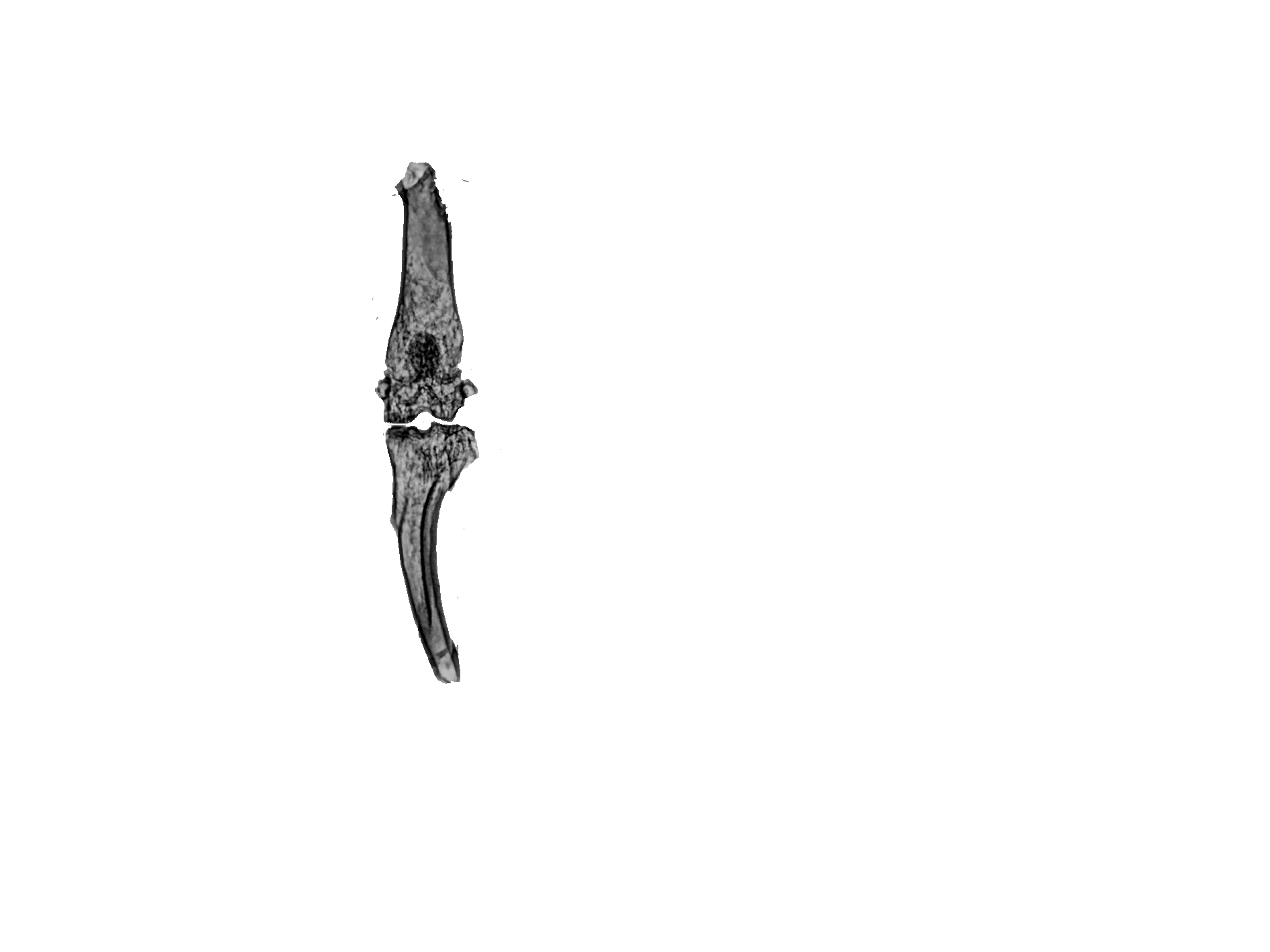

Supplement: Supplementary file 1 [file DataSheet1.zip › figure9/X-ray/DMM+Betulin.png]

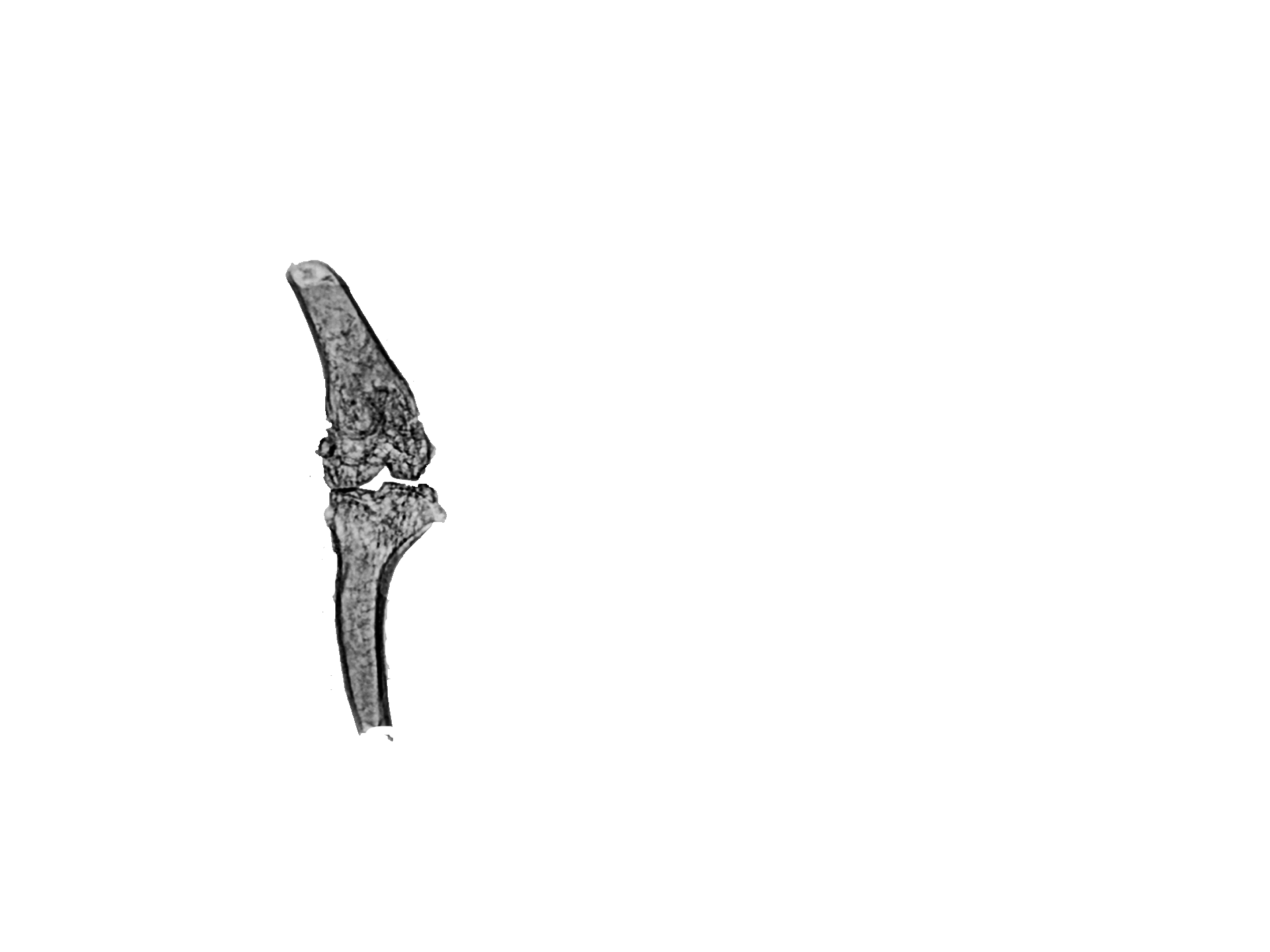

Supplement: Supplementary file 1 [file DataSheet1.zip › figure9/X-ray/DMM.png]

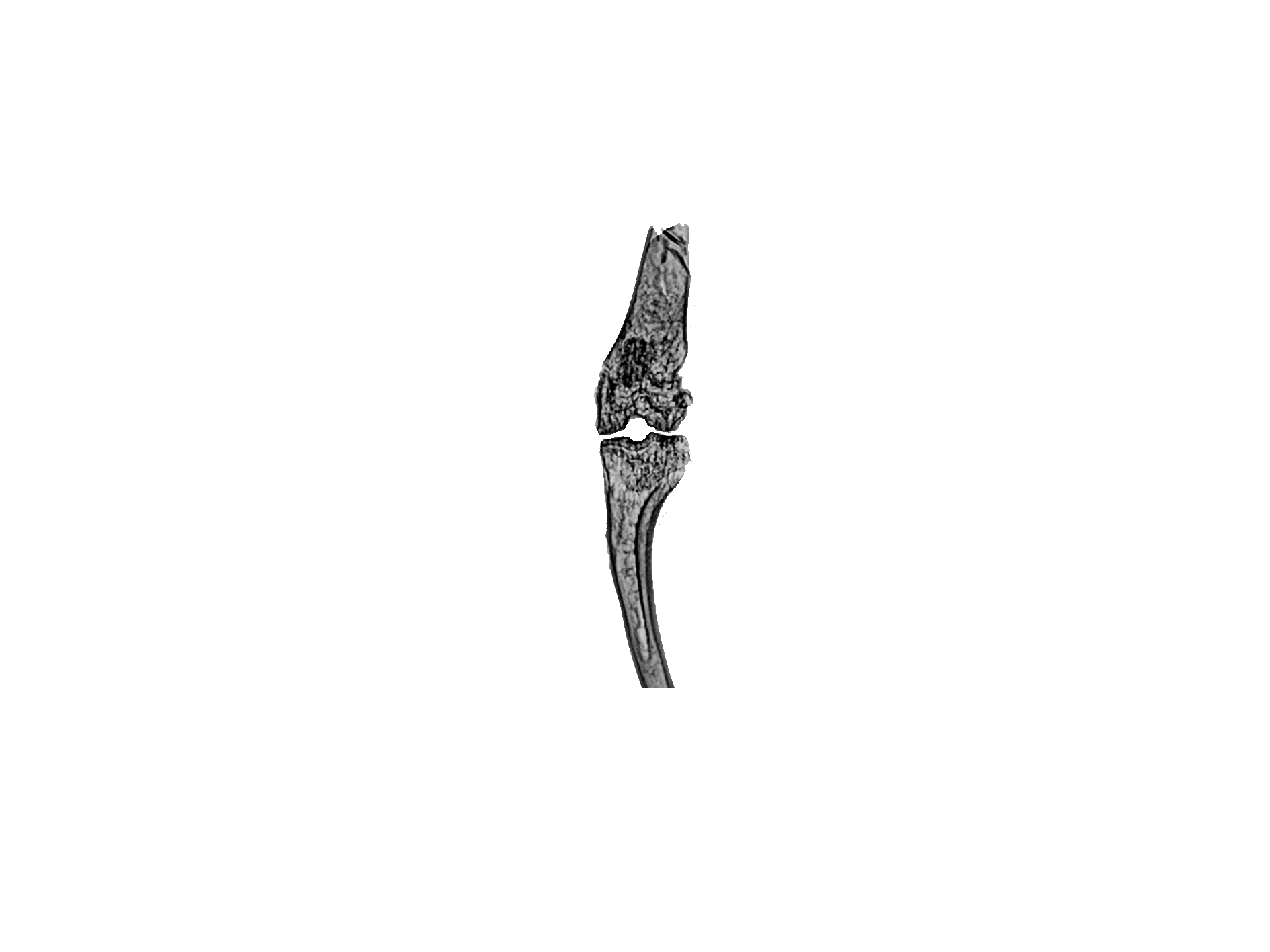

Supplement: Supplementary file 1 [file DataSheet1.zip › figure9/X-ray/Sham.png]
